# The Impact of the First and Second Wave of the COVID-19 Pandemic on Eating Symptoms and Dysfunctional Eating Behaviours in the General Population: A Systematic Review and Meta-Analysis

**DOI:** 10.3390/nu15163607

**Published:** 2023-08-17

**Authors:** Rubinia Celeste Bonfanti, Lucia Sideli, Arianna Teti, Alessandro Musetti, Stefania Cella, Nadia Barberis, Bianca Borsarini, Lucia Fortunato, Cristina Sechi, Nadia Micali, Gianluca Lo Coco

**Affiliations:** 1 Faculty of Human and Social Sciences, Kore University of Enna, 94100 Enna, Italy; rubiniaceleste.bonfanti@unikore.it; 2 Department of Human Science, LUMSA University, 00193 Rome, Italy; l.sideli@lumsa.it; 3 Department of Psychology, Educational Science and Human Movement, University of Palermo, 90128 Palermo, Italy; arianna.teti@unipa.it (A.T.); lucia.fortunato@unipa.it (L.F.); gianluca.lococo@unipa.it (G.L.C.); 4 Department of Humanities, Social Sciences and Cultural Industries, University of Parma, 43121 Parma, Italy; 5 Observatory on Eating Disorders, Department of Psychology, University of Campania “Luigi Vanvitelli”, 81100 Caserta, Italy; stefania.cella@unicampania.it; 6 Department of Health Sciences, Magna Graecia University of Catanzaro, 88100 Catanzaro, Italy; nbarberis@unicz.it; 7 Mental Health Services in the Capital Region of Denmark, Eating Disorders Research Unit, Psychiatric Centre Ballerup, 2750 Ballerup, Denmark; bianca.borsarini@regionh.dk (B.B.); nadia.micali@regionh.dk (N.M.); 8 Department of Pedagogy, Psychology, Philosophy, University of Cagliari, 09123 Cagliari, Italy; cristina.sechi@unica.it

**Keywords:** COVID-19, general population, eating disorders, dysfunctional eating behaviours, meta-analysis, systematic review

## Abstract

Background: The aim of this systematic review and meta-analysis was to examine the prevalence of feeding and eating disorder (FED) symptoms or dysfunctional eating behaviours (DEB) in the general population during the COVID-19 outbreak. Method: We searched eligible articles in biomedical databases from 1 January 2020 to 31 March 2022. Prevalence rates of FED or DEB changes between pre-pandemic and pandemic time and correlation with psychological distress were pooled with a meta-analysis using a random-effects model. Heterogeneity was tested using I-squared (*I*^2^) statistics. A total of 186 studies with 406,076 participants met the inclusion criteria. Results: The more prevalent FED or DEB during the COVID-19 outbreak were: body image concerns (52%, 95% CI 0.38, 0.66), binge eating (40%, 95% CI 0.25, 0.55), and overeating (40%, 95% CI = 0.32–0.48). Pooled data of longitudinal studies (k = 8) only showed a significant difference in the prevalence of weight gain from pre-pandemic to the pandemic time. Finally, increased levels of psychological distress (k = 35) positively correlated with some ED symptoms. Conclusion: This meta-analysis evidenced a negative impact of the pandemic on eating symptoms and DEB in the general population.

## 1. Introduction

The COVID-19 pandemic was an unprecedented event with many negative health consequences. There is substantial evidence that the pandemic has had an impact on mental health due to social restrictions, health anxiety, and fear of contagion, as well as worries about the economy [1,2,3]. The exacerbation of pre-exiting mental disorders has been documented, as well as the onset of novel mental distress conditions for the most vulnerable [2,4,5]. Both retrospective and longitudinal studies have reported mental health deterioration, with heightened symptoms of depression, anxiety, sleep problems, post-traumatic stress disorder, and psychological distress [1,6,7,8].

The negative effects of the pandemic for individuals with pre-existing mental health conditions, such as feeding and eating disorders (FEDs) [9], were particularly profound. Detrimental effects of COVID-19 related lockdowns for individuals with FEDs have been described in both adult populations [10,11,12,13,14,15] and, less extensively, adolescent populations [16,17,18,19,20]. Potential risk factors were identified in restriction to daily activities and movements, changes in food availability, limited exercise, excessive exposure to dysfunctional eating models on social media, pandemic-related emotional distress, and reduced access to treatment and care [21,22,23,24].

However, the negative effects of the COVID-19 pandemic on dysfunctional eating behaviours among the general population received less research attention. It was argued that the limited access to food stores, the greater time spent at home, and the feelings of boredom and anxiety triggered by the pandemic could have had effects on dietary styles and eating patterns, leading to irregular and emotional eating and more frequent snacking [25,26]. It is likely that the abrupt, traumatic lifestyle changes imposed by the pandemic have exacerbated some distinctive, eventually subthreshold, FED symptoms in relation to weight status, eating behaviours, physical activity, body dissatisfaction, and food relationship in individuals without any pre-existing FED [27]. Additionally, it was argued that the widespread increase and/or worsening of FED-like symptoms among the general population might have been a fertile ground for an increased risk of FED onset worldwide [27]. There was some initial support to this hypothesis from findings of several studies highlighting an alarming spread of FED behaviours in the general population [13,28,29,30,31,32,33,34,35]. More specifically, the negative impact of the pandemic on feeding and eating behaviours included weight gain and weight loss [28,29,30,31,33,35], snacking and increase in food intake [28,29,32,34,35], sedentarism and reduced physical activity [28,29,30], dietary changes [28,29,34,35], and problems with body image and eating concerns [13]. However, it is unclear whether the level of FED symptoms in the general population has changed across the different waves of the pandemic and across countries, as well as their association with psychological correlates. To date, five meta-analyses have examined the impact of the COVID-19 pandemic on eating symptoms among individuals with FEDs [14,36,37,38,39], but only two included data from the general population. Specifically, Haghshomar and colleagues [38] included studies on FED patients, individuals with other mental disorders, and non-clinical populations, reporting an overall prevalence of 9.37% (95% CI: 3.92%; 16.57%) of ED symptoms during the pandemic. Güzel and colleagues [37] included some non-clinical samples reporting frequencies only of binge eating and emotional eating outcomes. Finally, a meta-analysis on weight change during the pandemic [40] showed that the prevalence of obesity increased by 1%. The aim of this systematic review and meta-analysis is to estimate the pooled prevalence of several FED symptoms or dysfunctional eating behaviours (DEB) in the general population without any prior clinical diagnosis. Moreover, the current study aims to summarise the evidence on the levels of change in FED symptoms and DEB before and during the pandemic, and to examine the relationship between FED/DEB and psychological distress to identify potential correlates of worsening. Our aim was to add to the previous studies, none of which has comprehensively analysed the course of FED core symptoms [9] among the general population during the COVID-19 pandemic.

## 2. Methods

The review and meta-analysis were conducted according to the preferred reporting items for systematic reviews and meta-analyses (PRISMA) statement The protocol was registered in PROSPERO (CRD42022322532) “URL: https://www.crd.york.ac.uk/prospero (accessed on 1 April 2022)”.

### 2.1. Search Strategy

A systematic and comprehensive search was performed using the following databases: PubMed/Medline, ISI Web of Science, PsychInfo, EMBASE, and SCOPUS. Moreover, online searches on pre-print servers for unpublished papers were conducted. Searches were limited to studies published from January 2020 to March 2022. Search terms employed are described in the Supplementary Table S1.

### 2.2. Study Selection

All empirical studies that investigated the prevalence and course of FED core symptoms [9] (see also Supplementary Materials Data) among individuals in the general population during the COVID-19 pandemic were eligible, with the following inclusion criteria: (1) original articles, (2) written in English, (3) had empirical data on the prevalence of FED symptoms and DEb collected during the COVID-19 pandemic, (4) using cohort, case-control, cross-sectional study design. The outcomes of this review were the FED core symptoms [9]: overweight, weight gain, feeding/food restriction, undereating, food avoidance, appetite loss, fear of weight gain, feeling fat, body shape concern, body dissatisfaction, body misperception, weight loss, excessive physical activity, excessive exercise, compulsive exercise, caloric compensation, compensatory behaviours, vomit, binge eating, overeating, food cravings, snacking, night eating, and night feeding) mixed with specific DEb (e.g., emotional eating).

Publications were excluded if (1) they were not original articles (e.g., conference paper, proceeding, review, opinion paper, dissertation, case series, or case report), and (2) were carried out on clinical or mixed samples, including individuals with FED. Search results from each database were initially exported to EndNote, provided by Clarivate Analytics, and duplicates were identified and rejected. Thereafter, records were manually screened for titles and abstracts, and noncompliant titles were excluded. Finally, full-text articles were checked for eligibility criteria, and references of included studies were manually screened to obtain eventual additional articles.

### 2.3. Data Extraction and Analyses

R.B.C., B.B., L.F., C.S., and A.T. extracted data from the eligible studies into a customised Excel spreadsheet. The following information from studies was extracted: authors, year of publication, country, recruitment time and sample characteristics (sample size, mean age, percentage female, BMI, data collection method, type of outcome measure, follow-up period, and study findings). In cases of missing data, authors of the included studies were contacted for additional information. In this phase, quality data checks were conducted, attentive cross-checking of the extracted data, and unanimity decisions about methodology.

A meta-analysis was conducted to assess the overall prevalence of FED symptoms and DEB among the general population, changes of FED symptoms, and DEB from pre-pandemic to pandemic time, and correlation with psychological distress. A random-effect model (RE) with 95% confidence interval (95% CI) was used to estimate the pooled effect of the prevalence of FED and DEB. Only outcomes with data available from at least k = 3 studies or samples were included for meta-analysis. Heterogeneity was assessed using I-squared (*I*^2^) statistics, assuming 0–25%, 25–50%, and 50–75% *I*^2^ values corresponding to low, moderate, and high heterogeneity, respectively. Meta-regression and subgroup analyses were used to investigate the influence of the following continuous and categorical moderators: percentage of female participants, mean age, mean BMI, robustness of the studies (low, medium, and high), country (all continents), time of assessment (first, second, and third wave of pandemic), type of sample (adult, women, athletes, healthcare professionals, older adults, overweight and obese, and students), and outcome assessment (single item vs. standardised questionnaire). Funnel plot interpretation and Egger’s regression intercept [41] were used to assess publication bias.

All analyses were performed using Jamovi version 2.2.5 and JASP version 0.11.1.

### 2.4. Quality Assessment

The methodological quality of the included papers was assessed with a modified version of the Newcastle–Ottawa Scale [42] for observational studies (see Supplementary Table S2). The modified version of the NOS checklist assesses the methodological quality of papers with eight items. A maximum of nine points were attributed. Studies were evaluated to be at high risk of bias if scored equal or lower than four, at moderate risk of bias if scored five or six, and at low risk of bias if scored seven to nine. Quality assessment was conducted by R.B.C., B.B., L.F., C.S., and A.T. Any divergence between reviewers was discussed until an agreement was reached, and if needed, the senior authors were consulted (L.S., N.M., and G.L.C.). No studies in the present review were excluded based on poor methodological quality.

## 3. Results

**Figure 1:**
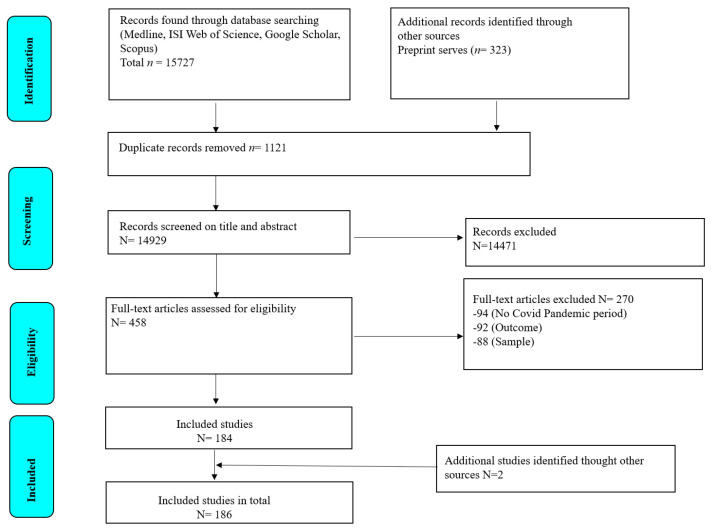
Preferred reporting items for systematic reviews and meta-analyses (PRISMA) flowchart of study selection.

A total of 16,050 records were identified through databases, pre-print servers, and manual search. After removing 1121 duplicates, 14,929 remaining titles and abstracts were screened, and 458 full-text articles were assessed for eligibility. A total of 186 articles met the inclusion criteria and were included in quantitative analyses (Figure 1).

### 3.1. Quality Appraisal

Supplementary Table S2 presents the quality ratings of included studies. In the sample, 52% of the studies were cross sectional descriptive studies, 26% of the studies were cross sectional analytic studies, 13% of the studies were longitudinal perspective studies (reporting a change in symptoms during the pandemic), and 9% longitudinal retrospective studies (reporting change in symptoms before and during the pandemic). Overall, 11% of the studies (N = 19; 33% longitudinal studies, 29% cross sectional descriptive studies, 29% cross sectional analytic studies, and 9% retrospective studies) fully satisfied the criteria for robustness, showing a low risk of bias. In addition, 35% (N = 65; 44% cross sectional descriptive studies, 28% cross sectional analytic studies, 19% longitudinal studies, and 9% retrospective studies) were evaluated at medium risk of bias, and a further 54% (N = 102; 61% cross sectional descriptive studies, 24% cross sectional analytic studies, 9% retrospective studies, and 6% longitudinal studies) had a high risk of bias.

Most studies reported a high selection bias, with 76% presenting inadequate recruitment strategies (e.g., snowballing recruitment strategy and use of convenience samples) and 42% reporting inadequate participation rate of the sample. The majority of studies (58%) used validated tools to assess the outcome (such as the Eating Disorder Examination Questionnaire—EDE-Q) [43], documented evidence (e.g., medical records or BMI), or an appropriate ad hoc tool (e.g., single question about weight gain). The majority of studies (93%) provided appropriate and complete statistical findings (64% controlled the analysis for basic socio-demographic variables and another 5% for additional potential confounders (e.g., BMI)).

### 3.2. Characteristics of the Studies

The characteristics of included articles are described in Supplementary Table S3. Results are divided into three sections, according to the type of outcome (i.e., (a) studies examining the prevalence of FED symptoms and DEB in the general population, followed by (b) studies examining FED symptoms and DEB change from pre-pandemic to pandemic time, or during the pandemic, and (c) studies examining the correlation between psychological distress and FED symptoms and DEB.

The total sample included 406,076 participants (females % = 65.2%; mean age = 33.54, range 18.7–74), and mean BMI = 25.25 (range 22.3–31.2). A total of 144 studies (78%) collected data between March and August 2020 during the first wave of the pandemic from both European and non-European countries (e.g., USA, China, Brazil, and others). Furthermore, 25 studies (14%) were conducted between September and December 2020 during the second wave of the pandemic, whereas the remaining 17 studies (8%) were conducted after January 2021 during the third wave of the pandemic. In total, 95 studies are cross sectional descriptive studies, 50 are cross sectional analytic studies, 25 are longitudinal studies, and 16 are retrospective studies.

Most of the studies (79.1%) involved adult participants belonging to the general population; the remaining studies included specific subgroups. Of these, 16 studies included students [44,45,46,47,48,49,50,51,52,53,54,55,56,57,58,59,60], 6 studies included healthcare professionals [61,62,63,64,65,66], 3 studies included elderly people [67,68,69], 2 studies included athletes [70,71], and 1 study included overweight and obese people not seeking treatment [72].

### 3.3. Prevalence of Eating Disorders Symptoms and Dysfunctional Eating Behaviours during the Pandemic

During the overall pandemic period, pooled prevalences of EDs and DEB were estimated as follows: binge eating behaviours (ES = 0.40, 95% CI = 0.25–0.55); overeating behaviours (ES = 0.40, 95% CI = 0.32–0.48); food craving (ES = 0.36, 95% CI = 0.13–0.60); body image concerns (ES = 0.52, 95% CI = 0.38–0.66); emotional eating behaviours (ES = 0.33, 95% CI = 0.26–0.41); overweight and weight gain (ES = 0.33, 95% CI = 0.31–0.36); snacking (ES = 0.31, 95% CI = 0.26–0.36); feeding or food restriction (ES = 0.28, 95% CI = 0.16–0.41); excessive physical activity (ES = 0.25, 95% CI = 0.20–0.31); weight loss (ES = 0.20, 95% CI = 0.18–0.22); and night eating behaviours (ES = 0.8, 95% CI = 0.05–0.11) (see Table 1 for details). As suggested by the funnel plots (see Supplementary Figures S1–S11) and Egger’s test, some estimates were associated with possible publication bias, specifically: weight gain (Egger’s test z = 2.76, *p* = 0.006), food restriction (z = 5.63, *p* ≤ 0.001), excessive physical activity (z = 3.99, *p* ≤ 0.001), binge eating (z = 2.04, *p* = 0.041), food craving (z = 2.90, *p* = 0.004), snacking (z = 2.70, *p* = 0.007), and night eating (z = −7.00, *p* ≤ 0.001).

Only two studies examined the prevalence of vomiting [60,73], with inconsistent results (symptoms ranging from 1% to 17%). A single study on caloric compensation and compensatory behaviours found that 3% experienced symptoms increasing during the confinement [74]. No studies estimated the prevalence of fear of weight gain.

The results of the meta-regression analysis showed that the following moderators had a significant influence (*p* < *0*.05 and 95% CI not including the null value) on the prevalence of FED and DEB during COVID-19 (see Tables S4–S13): risk of bias, time of assessment, country, outcome assessment, mean age of the sample, and percentage of female participants. A medium robustness of the study (k = 3, ES = −0.42, 95% CI = −0.76; −0.08, *p* ≤ 0.05) was associated with a lower prevalence of food cravings. The second wave of the pandemic (k = 3, ES = 0.30, 95% CI = 0.12; 0.49, *p* ≤ 0.001) was associated with a greater prevalence of excessive physical activity. Studies conducted in North America (k = 8, ES = 0.29, 95% CI = 0.19; 0.39, *p* ≤ 0.001) were associated with a greater prevalence of excessive physical activity. Finally, studies employing standardised questionnaires were associated with a lower prevalence of body shape concerns (k = 5, ES = −.24, 95% CI = −.48; −0.01, *p* ≤ 0.05) and a greater prevalence of overeating (k = 10, ES = 0.19, 95% CI = 0.04; 0.34, *p* ≤ 0.05).

### 3.4. Change in Symptoms before and during the Pandemic

Changes in the prevalence of FED symptoms and DEB from pre-pandemic to pandemic time or during the different waves of the COVID-19 pandemic were assessed in 35 studies, showing non-significant results (see Table 2), except for weight gain.

Retrospective studies assessing self-reported change in FED symptoms and DEB from pre-pandemic to the pandemic period suggested a significant change in weight gain (k = 8, SMD = 0.052, 95% CI = 0.03–0.08, *p* ≤ 0.001). No significant change was found for food restriction behaviours (k = 4, SMD = 0.496, 95% CI = −0.15; −1.17, *p* = 0.117) or for excessive exercising (k = 6, SMD = 0.599, 95% CI = −0.33; 1.53, *p* = 0.207). Due to the limited number of prospective studies assessing symptoms’ change during the pandemic, only weight gain was meta-analysed, with no evidence of a significant effect (k = 9, SMD = −0.002, 95% CI = −0.04; −0.00, *p* = 0.051). Retrospective studies showed significant heterogeneity (*I*^2^ range 37 to 96%). However, none of the examined moderators showed a significant effect. No evidence of publication bias was observed (see Table 2).

Some studies, which were not meta-analysed due to their limited number, revealed increased levels of overeating from pre-pandemic to pandemic time [75,76], and snacking [75]. No change in body shape concerns [46,77], body weight reduction [44,78], and binge eating was observed [79,80]. Only one study [81] reported decreased levels of binge eating during the second wave of the pandemic compared to the first wave. Studies on food craving [82,83] and emotional eating [76,81,82] reported mixed findings.

### 3.5. Correlates of ED Symptoms

A total of 35 studies examined the relationship between FEDs and domains of psychosocial distress during the COVID-19 outbreak (e.g., depression, anxiety, and post-traumatic stress symptoms) (Table 3). Specifically, five studies assessed COVID-related distress [53,84,85,86,87], whereas the majority assessed different domains of psychological distress in a short time lag (last 24 h or the “past week”), during the COVID-19 restrictions [50,76,83,88,89,90,91,92,93,94,95,96,97,98,99,100,101,102,103]. These psychopathological outcomes were grouped in the same meta-analysis because of the low number of studies. Evidence of positive correlation with psychosocial distress was found for weight gain (r = 0.28, 95% CI = 0.11–0.44) [52,53,76,86,87,89,91,99,104,105], body image concerns (r = 0.20, 95% CI = 0.00–0.40) [50,100,101], overeating (r = 0.36, 95% CI = 0.08–0.06), and emotional eating (r = 0.30, 95% CI = 0.22–0.38) [36,85,86,87,88,90,93,96,97,98,100,102,103].

The analyses suggested that many effect sizes were highly heterogeneous (*I*^2^ range 89.07 to 99.08%, see Table 3). Moderation analyses indicated that only female gender marginally reduced the association between FEDs and psychosocial distress.

Among studies not included in the meta-analysis, De Pasquale and colleagues (2021) reported a positive correlation between depression, compensatory behaviours, and binge eating. Ramalho [100] reported significant positive correlations between binge eating and depression, and between stress and anxiety.

## 4. Discussion

The current meta-analysis examined the prevalence of FED symptoms and DEB in the general population during the first and second waves of the COVID-19 pandemic. Generally, the results revealed a high prevalence of symptoms related to eating disorders. The COVID-19 pandemic had considerable impact on eating behaviours such as binge eating, overeating, emotional eating, food cravings, and feeding or food restriction. The pooled prevalence of body image outcomes, such as shape or weight concerns, was high. Overall, these results are in line with those from previous reviews, which supported an increase in eating disorder symptoms associated with the COVID-19 pandemic [13,106,107,108]. However, previous studies have mainly focused on individuals with pre-existing FED symptoms or some heterogeneous population consisting of patients with FED, patients with other mental disorders, and non-clinical participants [37,38]. The current study was the first to report pooled data on specific eating outcome types in the general population, and the findings add to the literature showing that the prevalence of FED and DEB was high during the pandemic time.

Regarding weight change, the current results suggest that the pooled prevalence of weight gain was 33% (95% CI = 0.31–0.36), whereas the prevalence of weight loss was 20% (0.18; 0.22). However, the few retrospective studies which evaluated weight change from pre- to post-pandemic showed an increase in this symptom over time. Our findings are in line with recent reviews, which reported an increase in weight among adults during the pandemic [33,109]. Other longitudinal studies focusing on other symptoms, both retrospective and prospective, showed inconsistent results. It is worth noting that most of these studies adopted a retrospective design and evidence was of low to moderate quality. This suggests that more longitudinal studies are needed [30] to achieve a good level of evidence. Moreover, it is worth noting that most of the studies captured the first year of the pandemic, and it is unclear whether this length of time is sufficient to capture weight change fluctuations after lockdown.

Our moderation analyses showed that females and individuals of younger age were more at risk of weight loss during the pandemic. Moreover, females were more at risk for food restriction and excessive exercise. Previous reviews showed an increased food restriction during the pandemic in five studies [38]. In our meta-analysis, we located 13 articles reporting a pooled prevalence of food restriction of 29%, and this finding may suggest a risk of exacerbation of symptoms related to anorexia nervosa in the general population [110]. However, the high prevalence of food restriction may also reflect the impact of home-confinement measures on a reduction of dietary intake, including dietary restrictions. Thus, further research efforts are needed to examine the link between food restriction behaviours, dietary changes, and irregular eating patterns during the pandemic [33,95].

The findings of the current meta-analysis also highlighted a high prevalence of overeating and snacking behaviours during the pandemic. These results are similar to those reported by previous reviews, which suggested that individuals tended to eat more during home confinement, with a high number of unhealthy snacks [29,34,35], mainly due to mood fluctuations or negative emotions experienced during the first wave of the pandemic [111].

We also found a pooled correlation between psychological distress levels (e.g., anxiety, depression) and overeating, emotional eating and weight gain, by suggesting that negative emotions may have played a role in deteriorating eating behaviours and eating styles during the pandemic. Excessive daily exercising has been highlighted as a concern during the pandemic, especially for people with FED [14,110,112]. Our results showed a pooled prevalence of 25% (95% C.I. 0.20–0.31%) for excessive physical activity, especially in studies conducted in USA, and during the second wave of the pandemic. This latter finding may be due to the ease of social restrictions and confinement in 2021 and the consequent re-opening of both outdoor and indoor sports centres, which impacted on individuals’ abilities to stay physically active. However, previous evidence showed that increased anxiety about exercise was a main concern for individuals with FED during the pandemic [113,114] and that excessive exercising was triggered by several messages shared online during the pandemic [115,116]. Overall, the current findings further highlight how the COVID-19-related restrictions may have negatively impacted on problematic eating habits during daily routines by influencing exercising practice [28]. However, it is worth noting that we found significant publication bias for food restriction, snacking behaviours, and excessive exercising, hence these findings should be interpreted with caution. Moreover, these findings suggest that the relationship between FEDs and domains of psychosocial distress holds across age and BMI, which strengthens the generalisability of the findings.

This meta-analysis found no significant change for any eating symptoms during the pandemic, except for weight gain from pre- to post-pandemic, which showed a slight increase. This could be explained by the diversity of assessment time, population characteristics, and lifestyles. Prior reviews focusing on individuals with eating disorders suggested a worsening in symptoms as a result of the pandemic [37,95], which has been mainly due to decreased access to care and treatment and to the negative influence of media and social isolation [13,117]. However, this negative pattern of deterioration appeared to be related to the stress related to the lockdown experience [14,106,118], whereas the impact of the COVID-19 outbreak on eating symptoms across the different pandemic waves remains elusive, with both negative and positive influences on individuals’ symptoms [13,106]. A large study of data from electronic health records in the USA showed an increased incidence of eating disorders in 2020 only for female adolescents and for anorexia nervosa [119]. Moreover, the authors found a decrease of eating disorders in the early part of 2020 which was followed by a steady increase throughout the rest of 2020. Accordingly, previous reviews on the worsening of mental health symptoms among the general population showed mixed findings, with some reviews suggesting a small overall increase in distress, which was larger among studies that assessed participants in the early stages of the pandemic (March–April 2020) [120], whereas others reported a slight decrease in anxiety and depression during the pandemic or no statistical change among the general population [1,5]. Overall, these results may suggest that after an acute distress response to the pandemic stress, there was a period of resilience and psychological adaptation after the first pandemic wave [74,121,122].

In our review, symptoms of FED were more commonly examined at the beginning of the outbreak, when individuals were challenged by mandatory lockdown or severe social restrictions. Follow-up studies after the first two years of the pandemic are strongly needed to assess the long-term impact of the pandemic on eating behaviours.

The major strength of the current meta-analysis is the high number of eligible studies and the large sample size of 406,076 participants, which estimated the impact of the COVID-19 pandemic on eating behaviours in the general population. However, there are several potential limitations to this meta-analysis. First, most of our observational data were collected through online surveys with a cross-sectional design. Although prevalence rates based on cross-sectional data are limited, in the current paper we examined 42 longitudinal or retrospective studies with data collected from pre-pandemic to pandemic time or during the different waves of the pandemic, reporting mixed evidence on the evolution of FED symptoms during the pandemic. Second, the majority of included studies were conducted during the first wave of the pandemic in 2020, and we cannot examine the long-term impact of the pandemic on eating symptoms. Although the current review focused on the prevalence of eating symptoms during the pandemic time, only 22% of the included studies reported data collected during the second and third wave of the pandemic. However, this was a common problem in prior meta-analyses during the pandemic, which showed that many publications addressing mental health issues were published at the beginning of the pandemic, whereas fewer studies were published from September 2020 to 2021 [1,5,120]. Further population-based longitudinal studies are needed to identify the evolution of FED symptoms across the pandemic waves, in order to identify the most vulnerable groups. Third, the generalisability of findings can be biased given the large percentage of women, which prevents gender-balanced outcome estimates. Similarly, the lack of data regarding pre-pandemic eating habits as well as ethnicity, race, and weight status, prevented considering these issues as potential risk factors or moderators for individual eating symptoms during the pandemic. There is previous evidence that individuals with transgender, sexual minority, and racial/ethnic minority identities, or those reporting higher levels of BMI, tend to exhibit greater ED-risk than heterosexual and White individuals [123]. However, the great majority of studies on the prevalence of eating disorders during the pandemic did not fully report on both their sample’s sex/gender and racial/ethnic breakdown. Therefore, further research is needed to explore whether eating disorder-related disparities changed since the pandemic outbreak. There is initial evidence that individuals who identified as gender/queer and lesbian exhibited heightened increases in ED symptoms from pre- to post-COVID onset compared to heterosexual individuals in a national study of US college students [124]. Future research may extend this work by examining whether these findings extend to other groups with stigmatised identities. Fourth, despite the exploration of different moderators, a high heterogeneity remained and too few studies reported consistent information which were suitable to be analysed. Considerable heterogeneity was especially observed across our analysis of cross-sectional data. However, this issue is frequent among studies concerning the prevalence of mental health symptoms during the COVID-19 pandemic [1,5,7,14], given the variety of measurement, demographic, and methodological moderators that can lead to large between-study variations in the reported prevalence. In the current review, we examined some potential sources of heterogeneity using a series of meta-regression analyses and identified several significant moderators (such as risk of bias, time of assessment, country, outcome measure, age, and gender) that may have contributed to the high heterogeneity. Other moderators could have been explored, such as social isolation, past traumatic experiences, dietary habits, and previous ED behaviours, but that information was not consistently reported in the large majority of studies, preventing us from analysing these relevant factors. Thus, the significant heterogeneity is an important limitation to this study. Regarding the association between eating symptoms and psychological distress, the great majority of the included studies used common measures of mental health symptoms instead of COVID-related stress. Although our meta-analysis focused on the general population, we cannot exclude the role of pre-existing mental health distress on the worsening of eating symptoms during the pandemic. Moreover, despite the large number of included studies, it is possible that some more recent studies were missed, given this evolving field. Thus, publication bias seemed evident in the analysis of some outcomes, with the risk of biased effect size estimates. In the current review, it is likely that the lack of inclusion of pre-prints and unpublished studies may have had an impact on publication bias, given the high number of ongoing studies on the topic.

Despite the aforementioned limitations, this meta-analysis on the prevalence of eating disorder symptoms and dysfunctional eating behaviours during COVID-19 is of major importance. It showed a high prevalence of eating symptoms among the general population and revealed how the pandemic fostered health problems that accompanied it. Prior evidence showed that eating symptoms deteriorated during the pandemic among individuals with pre-existing FED diagnosis [14,37,106,107,118]. The current results shed some light on the risk of heightened symptoms among the general population, although the analysis of longitudinal studies reported mixed findings without clear evidence of symptom deterioration during the pandemic. Moreover, the impact of COVID-19 seems highly heterogeneous, and further longitudinal studies are necessary to identify groups that are more at risk of experiencing eating disorder problems and whether the prevalence of symptoms reported in 2020 significantly changed as individuals were able to cope with pandemic stress.

## Figures and Tables

**Table 1:** Prevalence of EDs or DEB.

Domain	Outcome				
	k	ES (95% CI)	*p*	Q (*p*)	*I* ^2^
Weight Gain	84	0.33 (0.31; 0.36)	<0.001	18,969.675 (<0.001)	99.39%
Food Restriction	13	0.29 (0.16; 0.41)	<0.001	2282.463 (<0.001)	99.74%
Body Concerns	8	0.52 (0.38; 0.66)	<0.001	2062.509 (<0.001)	99.42%
Weight Loss	45	0.20 (0.18; 0.22)	<0.001	6926.623 (<0.001)	98.94%
Excessive Physical Activity	34	0.25 (0.20; 0.31)	<0.001	9591.382 (<0.001)	99.85%
Bingeing	11	0.40 (0.25; 0.55)	<0.001	2574.312 (<0.001)	99.7%
Overeating	28	0.40 (0.32; 0.48)	<0.001	9400.298 (<0.001)	99.71%
Food Craving	4	0.36 (0.13; 0.60)	<0.002	315.134 (<0.001)	99.49%
Snacking	44	0.31 (0.26; 0.36)	<0.001	836.339 (<0.001)	99.88%
Night Eating	3	0.08 (0.05; 0.11)	<0.001	48.781 (<0.001)	96.50%
Emotional Eating	17	0.33 (0.26; 0.41)	<0.001	75.931 (<0.001)	99.48%

**Table 2:** Changes in EDs or DEB before and during the pandemic.

Domain	Outcome					Moderators (z (95% CI), *p*)		
	k	SMD (95% CI)	*p*	Q (*p*)	*I* ^2^	%Female	Age	BMI
Weight Gain (pre-during pandemic)	8	0.052 (0.03; 0.08)	**<0.001**	4.569 (0.712)	0%	(k = 5) 0.00 (−0.01; 0.00), 0.561	(k = 4) −0.02 (−0.00; 0.01), 0.543	n.a.
Weight Gain (during pandemic)	9	−0.002 (−0.04; −0.00)	0.051	10.309 (0.239)	0%	(k = 9) 1.73 (−0.00; 0.00), 0.083	(k = 7) −0.17 (−0.00; 0.00), 0.864	(k = 6) 0.45 (−0.00; 0.00), 0.652
Food Restriction (pre-during pandemic)	4	0.496 (−0.15; 1.17)	0.117	3.976 (0.264)	37%	(k = 9) −0.026 (−0.06; 0.01), 0.126	n.a.	n.a.
Exercise Addiction (pre-during pandemic)	6	0.599 (−0.33; 1.53)	0.207	82.010 (<0.001)	96.4%	(k = 6) 1.01 (−0.02; 0.08), 0.315	n.a.	n.a.
Binge eating (during pandemic)	5	0.104 (−0.02; 0.22)	0.092	9.325 (0.053)	53.57%	(k = 4) 0.05 (−0.00; 0.01), 0.340	(k = 5) 0.01 (−0.00; 0.03), 0.169	n.a.

Note: significant results in bold. n.a.= Not sufficient number of studies available.

**Table 3:** Predictors of EDs or DEB during pandemic.

Domain	Outcome					Moderators (z (95% CI), *p*)		
	k	r (95% CI)	*p*	Q (*p*)	*I* ^2^	%Female	Age	BMI
Weight Gain and Psychological Distress	**10**	**0.277** (**0.11; 0.44**)	**<0.001**	1940.245 (<0.001)	99.08%	(k = 10) − 0.000 (−0.01; 0.01), 0.997	(k = 9) − 0.004 (−0.02; 0.02), 0.742	(k = 6) 0.006 (−0.02; 0.03), 0.612
Food Restriction and Psychological Distress	5	0.043 (−0.25; 0.33)	0.775	286.738 (<0.001)	98.89%	(k = 5)—(−0.02; 0.00) 0.113	n.a.	n.a.
Body Concerns and Psychological Distress	3	0.202 (0.00; 0.40)	0.043	17.638 (<0.001)	89.07%	n.a.	n.a.	n.a.
Overeating and Psychological Distress	3	**0.360** (**0.08; 0.06**)	**0.012**	24.862 (<0.001)	95.98%	n.a.	n.a.	n.a.
Emotional Eating and Psychological Distress	14	**0.299** (**0.22; 0.38**)	**<0.001**	21.546 (<0.001)	95.36%	(k = 14) −0.001 (−0.00; −0.00), 0.042	(k = 10) 0.013 (−0.00; 0.03), 0.109	(k = 8) 0.030 (−0.02; 0.08), 0.226

Note: significant results in bold. n.a.= Not sufficient number of studies available.

## Data Availability

The data presented in this study are available upon request from the corresponding author.

## Supplementary Materials

The following supporting information can be downloaded at: https://www.mdpi.com/article/10.3390/nu15163607/s1, Supplementary Table S1: Search strategy for each database; Supplementary Table S2: Quality assessment of included studies; Supplementary Table S3: Summary of characteristics of included studies; Supplementary Table S4: Meta-regression of factor affecting prevalence of weight gain; Supplementary Table S5: Meta-regression of factor affecting prevalence of food restriction; Supplementary Table S6: Meta-regression of factor affecting prevalence of body shape concerns; Supplementary Table S7: Meta-regression of factor affecting prevalence of weight loss; Supplementary Table S8: Meta-regression of factor affecting prevalence of excessive physical activity; Supplementary Table S9: Meta-regression of factor affecting prevalence of bingeing; Supplementary Table S10: Meta-regression of factor affecting prevalence of overeating; Supplementary Table S11: Meta-regression of factor affecting prevalence of food craving; Supplementary Table S12: Meta-regression of factor affecting prevalence of snacking; Supplementary Table S13: Meta-regression of factor affecting prevalence of emotional eating; Supplementary Figure S1: Forrest and Funnel Plot of weight gain in general population during the pandemic; Supplementary Figure S2: Forrest and Funnel Plot of food restriction in general population during the pandemic; Supplementary Figure S3: Forrest and Funnel Plot of body shape concerns in general population during the pandemic; Supplementary Figure S4: Forrest and Funnel Plot of weight loss in general population during the pandemic; Supplementary Figure S5: Forrest and Funnel Plot of excessive physical activity in general population during the pandemic; Supplementary Figure S6: Forrest and Funnel Plot of bingeing in general population during the pandemic; Supplementary Figure S7: Forrest and Funnel Plot of overeating in general population during the pandemic; Supplementary Figure S8: Forrest and Funnel Plot of food craving in general population during the pandemic; Supplementary Figure S9: Forrest and Funnel Plot of snacking in general population during the pandemic; Supplementary Figure S10: Forrest and Funnel Plot of night eating in general population during the pandemic; Supplementary Figure S11: Forrest and Funnel Plot of emotional eating in general population during the pandemic; Supplementary Figure S12: Forrest and Funnel Plot of change in weight gain from pre-pandemic to pandemic time; Supplementary Figure S13: Forrest and Funnel Plot of change in weight gain during the pandemic; Supplementary Figure S14: Forrest and Funnel Plot of change in food restriction from pre-pandemic to pandemic time; Supplementary Figure S15: Forrest and Funnel Plot of change in excessive physical exercise from pre-pandemic to pandemic time; Supplementary Figure S16: Forrest and Funnel Plot of change in excessive binge eating from pre-pandemic to pandemic time.
