# Supplementary material for: The Impact of the First and Second Wave of the COVID-19 Pandemic on Eating Symptoms and Dysfunctional Eating Behaviours in the General Population: A Systematic Review and Meta-Analysis
Source: Nutrients. 2023 Aug 17;15(16):3607. doi: 10.3390/nu15163607 (PMC10458761; doi:10.3390/nu15163607)
Supplement: Supplementary file 1 [file nutrients-15-03607-s001.zip › nutrients-2552231-supplementary.pdf]

Supplementary Materials

Supplementary Table S1

Search strategy for each database

Database	Search strategy
PubMed	((overweight) OR (weight gain) OR (feeding) OR (food restriction) OR (undereating) OR (food avoidance) OR (appetite loss) OR (fear of weight gain) OR (feeling fat) OR (body shape concern) OR (body dissatisfaction) OR (body misperception) OR (weight loss) OR (excessive physical activity) OR (excessive exercise) OR (compulsive exercise) OR (caloric compensation) OR (compensatory behaviours) OR (vomit) OR (bingeing) OR (binge eating) OR (overeating) OR (food craving) OR (snacking) OR (night eating) OR (night feeding) OR (emotional eating)) AND ((COVID) OR (COVID) 19" OR (COVID)-2019) OR (pandemic*) OR (SARSCoV2) OR (SARS-CoV) OR (coronavirus disease) OR (coronavirus) OR (Coronavirus Infections) OR (novel coronavirus) OR (HCoV) OR (severe acute respiratory syndrome coronavirus 2)) AND ((Lockdown) OR (lock-down) OR (Confinement) OR (Home-confinement) OR (Containment) OR (Quarantine) OR (Isolation) OR (social distancing) OR (physical distancing) OR (social isolation pandemic restrictions) OR (pandemic restriction) OR (physical Inactivity) OR (physical Inactive))
Embase	(overweight OR weight gain OR feeding OR food restriction OR undereating OR food avoidance OR appetite loss OR fear of weight gain OR feeling fat OR body shape concern OR body dissatisfaction OR body misperception OR weight loss OR excessive physical activity OR excessive exercise OR compulsive exercise OR caloric compensation OR compensatory behaviours OR vomit OR bingeing OR binge eating OR overeating OR food craving OR snacking OR night eating OR night feeding OR emotional eating) AND (COVID OR COVID 19 OR COVID-2019 OR pandemic* OR SARSCoV2 OR SARS-CoV OR coronavirus disease OR coronavirus OR Coronavirus Infections OR novel coronavirus OR HCoV OR severe acute respiratory syndrome coronavirus 2) AND (Lockdown OR lock-down OR Confinement OR Home-confinement OR Containment OR Quarantine OR Isolation OR social distancing OR physical distancing OR social

	isolation pandemic restrictions OR pandemic restriction OR physical Inactivity OR physical Inactive)
Web of Science	(overweight OR weight gain OR feeding OR food restriction OR undereating OR food avoidance OR appetite loss OR fear of weight gain OR feeling fat OR body shape concern OR body dissatisfaction OR body misperception OR weight loss OR excessive physical activity OR excessive exercise OR compulsive exercise OR caloric compensation OR compensatory behaviours OR vomit OR bingeing OR binge eating OR overeating OR food craving OR snacking OR night eating OR night feeding OR emotional eating) AND (COVID OR COVID 19 OR COVID-2019 OR pandemic* OR SARSCoV2 OR SARS-CoV OR coronavirus disease OR coronavirus OR Coronavirus Infections OR novel coronavirus OR HCoV OR severe acute respiratory syndrome coronavirus 2) AND (Lockdown OR lock-down OR Confinement OR Home-confinement OR Containment OR Quarantine OR Isolation OR social distancing OR physical distancing OR social isolation pandemic restrictions OR pandemic restriction OR physical Inactivity OR physical Inactive)
Scopus	“overweight” OR "weight gain" OR “feeding” OR "food restriction" OR “undereating” OR "food avoidance" OR "appetite loss" OR “fear of weight gain” OR “feeling fat” OR “body shape concern” OR “body dissatisfaction” OR “body misperception” OR “weight loss” OR “excessive physical activity” OR “excessive exercise” OR “compulsive exercise” OR "caloric compensation” OR “compensatory behaviours” OR “vomit” OR “bingeing” OR “binge eating” OR “overeating” OR "food craving" OR “snacking” OR “night eating” OR “night feeding” OR “emotional eating” AND “COVID” OR “COVID 19” OR “COVID-2019” OR “pandemic*” OR “SARSCoV2” OR “SARS-CoV” OR “coronavirus disease” OR “coronavirus” OR “Coronavirus Infections” OR “novel coronavirus” OR “HCoV” OR “severe acute respiratory syndrome coronavirus 2" AND “Lockdown” OR “lock-down” OR “Confinement” OR “Home-confinement” OR “Containment” OR “Quarantine” OR “Isolation” OR “social distancing” OR “physical distancing” OR “social isolation pandemic restrictions” OR “pandemic restriction” OR “physical Inactivity” OR “physical Inactive”

Supplementary Materials

Supplementary Table S2

Quality assessment of included studies

author, date	study design	Representativeness of the sample	Sample size	Non-respondents	Ascertainment of the exposure	Confounders	Assessment of the outcome	Statistical analyses	Independent longitudinal assessment	Total score	Risk of bias
Abdulsalam et al., 2021	retrospective	0	0	0	1	1	0	1	1	4	High
Abed Alah et al., 2021	cross sectional	0	1	0	1	1	0	1	0	4	High
Agurto et al., 2021	cross sectional	0	0	0	1	0	1	1	0	3	High
Al Domi et al., 2021	cross sectional	0	1	0	1	1	1	1	0	5	Medium
Al Musharaf, 2020	cross sectional	1	1	0	1	2	1	1	0	7	Low
Al Musharaf et al., 2021	longitudinal	0	1	0	1	2	1	1	1	7	Low
Al Saleh et al., 2021	cross sectional	1	1	0	1	1	1	1	0	6	Medium
Alafif et al., 2021	cross sectional	1	1	0	1	1	1	1	0	6	Medium
Aldhuwayhi et al., 2022	cross sectional	0	0	0	1	1	0	1	0	3	High
Alfawaz et al., 2021	retrospective	0	1	0	1	1	1	1	1	6	Medium
Ali et al., 2021	cross sectional	1	1	0	1	1	1	1	0	6	Medium
AlMughamis et al., 2020	cross sectional	0	0	0	1	1	0	1	0	3	High
AlTarrah et al., 2021	cross sectional	0	1	0	1	1	1	1	0	5	Medium
Álvarez-Gómez et al., 2021	cross sectional	0	0	0	1	1	0	1	0	3	High
Ammar et al., 2020	retrospective	0	1	0	1	1	1	1	1	6	Medium
Arriola-Torres et al., 2021	cross sectional	0	0	0	1	1	0	1	0	3	High

Baceviciene & Jankauskiene, 2021	longitudinal	0	0	1	1	1	1	1	1	6	Medium
Bajpeyi et al., 2021	cross sectional	0	0	0	1	0	0	1	0	2	High
Bakhsh et al., 2021	cross sectional	1	1	0	1	1	0	1	0	5	Medium
Barcın-Güzeldere & Devrim-Lanpir, 2022	cross sectional	1	1	0	1	2	1	1	0	7	Low
Bemanian et al., 2020	cross sectional	1	1	1	1	1	0	1	0	6	Medium
Bhutani et al., 2021a	longitudinal	0	1	0	1	1	1	1	1	6	Medium
Bhutani et al., 2021b	cross sectional	0	1	0	1	1	1	1	0	5	Medium
Bianchi et al., 2022	retrospective	1	1	0	1	1	1	1	1	7	Low
Bicer et al., 2021	retrospective	0	1	0	1	1	1	1	1	6	Medium
Bin Zara et al., 2020	cross sectional	0	1	0	1	2	1	1	0	6	Medium
Błaszczyk-Bebenek et. al, 2020	retrospective	0	0	0	1	1	0	1	1	4	High
Bolesławska et al., 2021	retrospective	0	0	0	1	0	0	1	1	3	High
Boukrim et al., 2021	cross sectional	0	0	0	1	0	0	1	0	2	High
Breiner et al., 2021	retrospective	0	0	0	1	1	1	1	1	5	Medium
Brito et al., 2021	cross sectional	0	1	0	1	1	0	1	0	4	High
Buckland & Kemps, 2021	cross sectional	0	1	0	1	0	1	1	0	4	High
Buckland et al., 2021	cross sectional	0	1	0	1	1	1	1	0	5	Medium
Buckley et al., 2021	cross sectional	0	0	0	1	0	1	1	0	3	High
Cardi et al., 2021	cross sectional	0	0	0	1	1	0	1	0	3	High
Carroll et al., 2020	cross sectional	0	0	1	1	1	0	1	0	4	High
Caso et al., 2022	longitudinal	0	1	1	1	2	1	1	1	8	Low

Castellini et al., 2020	longitudinal	0	0	0	1	1	1	1	1	5	Medium
Cecchetto et al., 2021	longitudinal	0	0	0	1	1	1	1	1	5	Medium
Chan & Chiu, 2022	cross sectional	0	0	0	1	0	1	1	0	3	High
Chee et al., 2020	cross sectional	0	0	0	1	1	1	1	0	4	High
Cheikh Ismail et al., 2020	retrospective	0	1	1	1	1	1	1	1	7	Low
Cheikh Ismail, Hashim,et al., 2021a	cross sectional	0	1	0	1	1	1	1	0	5	Medium
Cheikh Ismail, Osaili, et al., 2021b	cross sectional	0	1	0	1	1	1	1	0	5	Medium
Chen et al., 2021	cross sectional	1	0	1	1	1	1	1	0	6	Medium
Cherick et al., 2020	cross sectional	0	1	0	1	0	0	0	0	2	High
Christensen et al., 2021	cross sectional	0	0	0	0	1	1	1	0	3	High
Cirillo et al., 2021	retrospective	0	0	0	1	0	0	1	0	2	High
Coakley et al. 2021	cross sectional	0	1	1	1	0	1	1	0	5	Medium
Constant et al., 2020	cross sectional	1	1	1	0	1	0	0	0	4	High
Cooper et al., 2021	cross sectional	0	1	0	1	1	1	1	0	5	Medium
C. dos S. Costa et al., 2021	longitudinal	1	1	1	1	1	1	0	1	7	Low
Costa M. L. et al., 2021	cross sectional	0	0	1	1	1	1	1	0	5	Medium
Coulthard et al., 2021	cross sectional	0	0	1	1	1	1	1	0	5	Medium
Cruceanu & Georgescu, 2021	cross sectional	0	0	0	1	0	0	0	0	1	High
Cummings et al., 2021	cross sectional	1	1	1	1	1	1	1	0	7	Low
Czepczor-Bernat et al., 2021	cross sectional	0	0	1	1	1	1	1	0	5	Medium
Da Rocha et al., 2021	longitudinal	0	0	0	1	0	0	0	0	1	High

De Pasquale et al., 2021	cross sectional	0	0	0	1	1	1	1	0	4	High
Deschasaux-Tanguy et al., 2021	cross sectional	1	1	1	1	1	0	1	1	7	Low
Di Renzo et al., 2020	cross sectional	1	0	1	1	1	1	1	0	6	Medium
Dicken et al., 2021	longitudinal	0	1	1	1	1	0	1	0	5	Medium
Dobrowolski & Włodarek, 2021	cross sectional	1	0	0	0	0	0	0	0	1	High
Đogaš et al., 2020	cross sectional	1	1	1	1	0	0	1	0	5	Medium
Dores et al., 2021	cross sectional	1	1	1	1	1	1	1	0	7	Low
Dor-Haim et al., 2021	cross sectional	0	1	0	1	1	0	1	0	4	High
dos Santos Quaresma et al., 2021	cross sectional	1	1	0	1	1	1	1	0	6	Medium
Dragun et al., 2020	cross sectional	0	1	0	1	1	0	0	1	4	High
Drieskens et al., 2021	cross sectional	1	1	0	1	1	0	1	0	5	Medium
Drywień et al., 2020	cross sectional	0	1	1	1	1	0	1	0	5	Medium
Du et al., 2022	cross sectional	0	1	0	1	0	0	1	0	3	High
Dun et al., 2021	longitudinal	0	1	1	1	0	0	1	1	5	Medium
Ekpanyaskul & Padungtod, 2021	cross sectional	0	1	0	1	0	0	1	0	3	High
Elangovan et al., 2020	cross sectional	0	1	1	1	0	0	1	0	4	High
Elmacıoğlu et al., 2021	cross sectional	1	1	0	1	1	1	1	0	6	Medium
Enriquez-Martinez et al., 2021	cross sectional	1	1	1	1	0	0	1	0	5	Medium
Flaudias et al., 2020	cross sectional	0	1	0	1	0	1	1	0	4	High
Freitas et al., 2021	longitudinal	0	0	1	1	0	1	1	1	5	Medium
Gao et al., 2022	cross sectional	0	1	0	1	1	1	1	0	5	Medium
Grant et al., 2021	cross sectional	0	1	0	1	0	0	0	0	2	High

Guerrini Usubini et al., 2021	cross sectional	0	0	0	1	1	1	1	0	4	High
Jackson A. M. et al., 2022	cross sectional	0	0	0	1	1	0	1	0	3	High
Jackson A. et al., 2021	cross sectional	0	0	0	0	1	1	1	0	3	High
Jordan et al., 2021	cross sectional	0	0	0	0	1	1	1	0	3	High
Karakose et al., 2021	cross sectional	0	0	0	1	0	1	1	0	3	High
Kaufman-Shriqui et al., 2022	cross sectional	0	1	0	0	0	0	1	0	2	High
Kesilmi et al., 2021	cross sectional	0	0	0	0	0	1	1	0	2	High
Khubchandani et al., 2020	cross sectional	0	0	0	0	0	0	1	0	1	High
Landaeta-Díaz et al., 2021	cross sectional	1	1	1	0	1	0	1	0	5	Medium
León-Paucar et al., 2021	cross sectional	0	0	0	0	1	1	1	0	3	High
Liboredo et al., 2021	cross sectional	0	1	1	1	1	1	1	0	6	Medium
Li et al., 2021	longitudinal	1	1	1	0	1	1	1	1	7	Low
Lofrano-Prado, et al., 2021	cross sectional	0	1	1	0	0	0	1	0	3	High
Ma et al., 2021	cross sectional	1	1	0	1	1	0	1	0	5	Medium
Madalı, et al., 2021	cross sectional	0	1	0	1	0	1	1	0	4	High
Madan et al., 2021	longitudinal	0	1	0	1	1	0	1	0	4	High
Maffoni et al., 2021	cross sectional	0	1	1	0	0	0	1	0	3	High
Mahar et al., 2021	cross sectional	0	0	0	0	0	1	1	0	2	High
Malkawi et al., 2021	cross sectional	0	1	0	0	0	0	1	0	2	High
Martínez-de-Quel et al., 2021	longitudinal	0	0	0	0	1	1	1	1	4	High
Mason et al., 2021	cross sectional	0	1	1	0	1	0	1	1	5	Medium
Mazzolani et al. 2021	longitudinal	0	1	1	1	2	1	1	1	8	Low
McAtamney et al., 2021	cross sectional	0	0	1	0	1	1	1	0	4	High

Micheletti Cremasco et al., 2021	cross sectional	0	1	1	1	1	0	1	0	5	Medium
Molina-Montes et al., 2021	cross sectional	1	1	0	1	0	1	1	0	5	Medium
Mota et al., 2021	cross sectional	1	1	0	1	1	0	1	0	5	Medium
Mulugeta et al., 2021	longitudinal	0	1	1	0	1	0	1	1	5	Medium
Mumena, 2020	retrospective	1	1	1	0	0	0	1	0	4	High
Nitu et al., 2021	longitudinal	1	0	1	0	0	0	1	0	3	High
Özcan & Yeşİlkaya, 2021	cross sectional	1	0	0	0	1	1	1	0	4	High
Özden & Parlar Kiliç, 2021	cross sectional	0	1	1	0	1	1	1	0	5	Medium
Özen et al., 2021	cross sectional	0	0	0	0	1	1	1	0	3	High
Ozenoglu et al., 2021	cross sectional	0	0	0	0	1	1	1	0	3	High
Pak et al., 2022	cross sectional	0	0	0	1	1	1	1	0	4	High
Palmer et al., 2021	retrospective	0	1	1	0	1	0	1	0	4	High
Pappa et al., 2021	cross sectional	0	0	0	0	1	0	1	0	2	High
Pertuz-Cruz et al., 2021	cross sectional	0	1	0	0	1	0	1	0	3	High
Phillipou et al., 2020	cross sectional	0	1	0	0	1	1	1	0	4	High
Phillipou et al., 2021	cross sectional	0	1	0	0	0	1	1	0	3	High
Pirutinsky et al., 2021	cross sectional	0	1	0	0	0	1	1	0	3	High
Pisot et al., 2020	cross sectional	0	1	0	0	1	0	1	0	3	High
Poelman et al., 2021	cross sectional	0	1	0	0	1	1	1	0	4	High
Pompili et al., 2022	cross sectional	0	0	0	0	1	0	1	0	2	High
Pop et al., 2021	longitudinal	0	0	0	0	0	0	1	1	2	High
Prezotti et al., 2021	cross sectional	0	0	1	1	0	0	0	0	2	High
Puhl et al., 2020	longitudinal	0	0	1	1	2	1	1	1	7	Low
Queiroz et al., 2021	cross sectional	0	0	0	1	1	1	1	0	4	High
Radwan et al., 2021	cross sectional	1	1	1	1	1	0	1	0	6	Medium

Ramalho et al., 2022	cross sectional	0	0	0	1	1	1	1	0	4	High
Reyes-Olavarría et al., 2020	cross sectional	0	1	0	1	1	0	1	0	4	High
Robertson et al., 2021	cross sectional	0	0	0	1	1	0	1	0	3	High
Robinson et al., 2021	cross sectional	1	1	1	1	2	1	1	0	8	Low
Robinson et al., 2020	cross sectional	1	1	1	1	1	0	1	0	6	Medium
Rodriguez-Perez et al., 2020	cross sectional	0	1	1	1	1	1	1	0	6	Medium
Rogers et al., 2021	longitudinal	0	0	0	1	1	1	1	1	5	Medium
Ruiz-Zaldibar, et al., 2022	cross sectional	1	0	0	0	1	1	1	0	4	High
Ruiz et al., 2021	cross sectional	1	1	1	1	0	1	0	0	5	Medium
Sadler et al., 2021	cross sectional	1	0	1	1	1	1	1	0	6	Medium
Sánchez et al., 2021	cross sectional	1	1	1	1	0	1	1	0	6	Medium
Sánchez-Sánchez et al., 2020	cross sectional	1	1	1	1	0	1	1	1	7	Low
Sánchez-Sánchez et al., 2021	cross sectional	0	0	1	1	1	1	0	0	4	High
Santana et al., 2021	cross sectional	0	1	1	1	1	1	1	1	7	Low
Sarda et al., 2022	cross sectional	1	1	0	1	0	0	1	0	4	High
Saxena et al., 2021	cross sectional	0	0	0	1	0	0	0	0	1	High
Scacchi et al., 2021	cross sectional	1	1	1	1	0	1	1	0	6	Medium
Scarmozzino & Visioli, 2020	cross sectional	1	1	1	1	0	0	0	0	4	High
Scharmer et al., 2020	cross sectional	1	0	0	1	0	1	1	0	4	High

Schulte et al., 2022	cross sectional	0	0	1	1	0	1	1	0	4	High
Seal et al., 2022	longitudinal	1	1	0	1	1	0	1	1	6	Medium
Sebastião et al., 2022	cross sectional	0	0	0	1	1	0	1	0	3	High
Serin et al., 2020	cross sectional	0	1	0	1	0	1	1	0	4	High
Shaun et al., 2021	retrospective	0	0	0	1	1	1	1	1	5	Medium
Shibata et al., 2021	cross sectional	0	1	0	1	1	1	1	0	5	Medium
Shin, 2021	cross sectional	0	0	0	1	1	0	1	0	3	High
Sidor et al., 2020	cross sectional	0	1	0	1	0	1	1	0	4	High
Silva et al., 2021	cross sectional	1	1	0	1	0	0	1	0	4	High
Silverman & Wang, 2021	cross sectional	0	0	0	1	0	1	1	1	4	High
Skotnicka et al., 2021	retrospective	0	1	0	0	2	0	1	0	4	High
Smith et al., 2021	cross sectional	0	0	1	0	1	0	1	0	3	High
Sobba et al., 2021	retrospective	0	1	1	0	1	0	1	0	4	High
Solè et al., 2021	cross sectional	0	0	0	1	1	0	1	0	3	High
Suka et al., 2021	cross sectional	1	1	1	1	1	1	1	0	7	Low
Sulejmani et al., 202	cross sectional	0	0	0	1	1	0	1	0	3	High
Swami et al., 2021	cross sectional	0	0	0	1	1	1	1	0	4	High
Tabler et al., 2021	cross sectional	0	0	1	1	0	1	1	1	5	Medium
Tan et al., 2021	retrospective	0	1	0	1	1	1	1	0	5	Medium
Tfifha et al., 2021	cross sectional	0	0	0	1	1	0	1	0	3	High
Thahir et al., 2021	cross sectional	0	1	0	1	1	0	1	0	4	High
Trott et al., 2021	longitudinal	0	0	0	1	0	1	1	1	4	High
Turgut et al., 2020	cross sectional	0	0	0	1	0	1	1	0	3	High
Urzeala et al., 2022	longitudinal	0	1	0	1	1	1	1	1	6	Medium
Vacca et al., 2021	cross sectional	0	1	0	1	1	1	1	0	5	Medium
Valencia et al., 2021	cross sectional	0	0	0	1	0	0	1	0	2	High

Vidal et al., 2021	cross sectional	0	1	0	1	0	1	1	0	4	High
Visser et al., 2020	cross sectional	1	1	1	1	1	1	1	1	8	Low
Wang et al., 2022	cross sectional	0	0	0	1	1	1	1	0	4	High
Yang et al., 2020	longitudinal	0	1	0	1	0	1	1	1	5	Medium
Yılmaz Akyüz et al., 2021	cross sectional	0	1	0	1	1	1	1	0	5	Medium
Yilmaz & Sanlier, 2021	cross sectional	0	0	0	1	1	1	1	0	4	High
Yokoro & Wakimoto, 2021	cross sectional	0	0	1	1	0	0	1	0	3	High
Zach & Fernandez, 2021	cross sectional	0	0	0	1	1	0	1	0	3	High
Zachary et al., 2020	cross sectional	0	1	0	1	1	0	1	0	4	High
Zhang & Zhang, 2020	cross sectional	0	1	1	1	1	1	1	0	6	Medium
Zhou & Wade, 2021	longitudinal	0	1	1	1	1	1	1	1	7	Low
Zhu et al., 2021	retrospective	0	0	1	1	1	0	1	0	4	High
Zielinska & Luszczki, 2021	cross sectional	0	1	0	1	1	1	1	0	5	Medium

Supplementary Table S3

Summary of characteristics of included studies

author, date	country	recruitment time	N	Female, n (%)*	Age, n/ mean*	Type of sample	BMI	data collection	Outcome definition	Outcome assessment
**Abdulsalam et al., 2021**	Saudi Arabia	COVID-19 curfew period	472	321 (68)	na	Adults	na	Online survey	Weight Gain; Weight Loss; Excessive physical activity; Night eating	Single item/ad hoc questionnaire
**Abed Alah et al., 2021**	Qatar	December, 2020 - February, 2021	1408	580 (41.2)	na	Adults	na	Online survey	Weight Gain	Single item/ad hoc questionnaire
**Agurto et al., 2021**	Peru	July 2020	686	569 (82.9)	na	Adults	25.97	Online survey	Weight Gain	Eating Habits Questionnaire
**Al Domi et al., 2021**	Jordania	March, 2020 – April, 2020	4473	3086 (70.3)	na	Adults	na	Online survey	Weight Gain; Weight Loss; Excessive physical activity; Snacking; Increase Appetite	Single item/ad hoc questionnaire
**Al Musharaf, 2020**	Saudi Arabia	May 18, 2020 - May 28, 2020	638	638 (100)	22.0	Women	23.2	Online survey	Emotional eating	Emotional Eating Scale
**Al Musharaf et al., 2021**	Saudi Arabia	T1: February, 2019 - April, 2019; T2: April, 2020 – May, 2020	297	297 (100)	20.7	Women	23.0	Phone interview	Weight Gain	Saudi Food and Drug Administration's food frequency questionnaire (SFDA-FFQ)
**Al Saleh et al., 2021**	Saudi Arabia	March 15, 2020 – April 30, 2020	1641	908 (55.3)	na	Adults	na	Online survey	Weight Gain; Weight Loss; Excessive physical activity	Single item/ad hoc questionnaire
**Alafif et al., 2021**	Saudi Arabia	March 23, 2020 - June 21, 2020.	733	577 (78.7)	21	Students	na	Online survey	Weight Gain; Snacking; Emotional eating	COVIDiet Questionnaire, Mediterranean Diet Adherence Screener (MEDAS); Three-Factor Eating Questionnaire
**Aldhuwayhi et al., 2022**	Saudi Arabia	na	206	63 (31)	na	Students	na	Online survey	Binge Eating	Single item/ad hoc questionnaire
**Alfawaz et al., 2021**	Saudi Arabia	May 11, 2020 - June 6, 2020	1965	1044 (53.0)	na	Adults	na	Online survey	Excessive physical activity; Snacking	Single item/ad hoc questionnaire
**Ali et al., 2021**	Pakistan	na	1956	1410 (72.1)	na	Adults	na	Online survey	Weight Gain	Single item/ad hoc questionnaire
**AlMughamis et al., 2020**	Kuwait	April 2, 2020 – April 12, 2020	522	380 (72.8)	11.75	Adults	na	Online Survey	Weight gain; Snacking; Excessive physical activity	Single item/ad hoc questionnaire

**AlTarrah et al., 2021**	Kuwait	July 28, 2020 - August 31, 2020	841	655 (77.9)	na	Adults	na	Online Survey	Overeating; Undereating; Snacking	Single item/ad hoc questionnaire
**Álvarez-Gómez et al., 2021**	Spain	March 15, 2021 -April 4, 2021	510	381 (74.7)	5.1	Adults	na	Online Survey	Overeating; Snacking; Physical activity; Weight gain; Weight loss	Single item/ad hoc questionnaire
**Ammar et al. 2020**	Asia, Africa, Europe, others	April 1, 2020 - April 6. 2020.	1047	563 (53.8)	na	Adults	na	Online Survey	Eating out of control; Snacks between meals	International Physical Activity Questionnaire Short Form (IPAQ-SF); Short Diet Behaviours Questionnaire for Lockdowns (SDBQL);
**Arriola-Torres et al., 2021**	Peru	July 01, 2020 – July 11, 2020	107	58 (54.6)	4.40	Health Workers	na	Online Survey	Food cravings	Single item/ad hoc questionnaire
**Baceviciene & Jankauskiene, 2021**	Lithuania	T1: October 2019; T2: February 2021	230	182 (79.1)	5.4	Students	22.32	Online Survey	Disordered eating; overeating; Having unhealthy snacks; Eating late at night	EDE-Q 6.0; Sociocultural Attitudes Towards Appearance Questionnaire-4 (SATAQ-4)
**Bajpeyi et al., 2021**	Texas	October-December 2020	58	48 (82.8)	6.8	Older adults	29.7	Telephone Interview	Weight gain; Weight loss	Single item/ad hoc questionnaire
**Bakhsh et al., 2021**	Saudi Arabia	June - July 2020	2255	1453 (64)	na	Adults	28.1	Online Survey	Weight gain; Weight loss; Snacking; Overeating; Physical activity	Single item/ad hoc questionnaire
**Barcın-Güzeldere & Devrim-Lanpir, 2022**	Turkey	June - September 2020	506	387 (76.5)	11.48	Adults	24.22	Online Survey	Emotional eating	Emotional Eater Questionnaire
**Bemanian et al., 2020**	Norway	April 15, 2020 - April 30, 2020	24968	13982 (56)	na	Adults	na	Online Survey	Emotional eating	Single item/ad hoc questionnaire
**Bhutani et al., 2021a**	USA	T0: 24 April to 4 May 2020 T1: 21 September to 13 October 2020	727	388 (53.37)	na	Adults	26.38	Online Survey	Overweight, Weight Gain; Weight Loss; Food cravings; Snacking	Control of Eating Questionnaire (CoEQ)
**Bhutani et al., 2021b**	USA	April 24, 2020 –May 4, 2020	1609	911 (56.62)	na	Adults	na	Online Survey	Food cravings	Control of Eating Questionnaire (CoEQ)
**Bianchi et al., 2022**	Italy	April 13, 2020 - May 19, 2020	1925	1384 (71.9)	2.75	Adults	na	Online Survey	Bingeing, Binge Eating	Binge Eating Disorder Screener (BEDS-7)
**Bicer et al., 2021**	Turkey	March 15, 2020 -May 15, 2020	2955	2805 (94.9)	10.34	Adults	23.9	Online Survey	Weight gain; Weight loss; Cognitive Restriction;	Three Factor Eating Questionnaire (TFEQ)

									Emotional eating; Uncrontrolled eating	
**Bin Zara et al., 2013**	United States	April, 2020 – June, 2020	3133	2462 (79.4)	na	Adults	30.73	Online Survey	Overweight, Weight Gain; Weight Loss; Excessive physical activity, Excessive exercise, Compulsive exercise; Vomit	Dana-Farber's Cancer Institute Eating Habits Questionnaire; Yale Food Addiction Scale; USDA Food Security Module
**Błaszczyk-Bebenek et al., 2020**	Poland	April 29, 2020- May 19, 2020	312	200 (64.1)	13.05	Adults	25.28	Online Survey	Weight gain; Weight loss	Single item/ad hoc questionnaire
**Bolesławska et al., 2021**	Poland	April 19, 2020 - May 19, 2020	312	200 (64.1)	na	Adults	24.78	Online Survey	Weight gain; Weight loss	Single item/ad hoc questionnaire
**Boukrim et al., 2021**	Morocco	April 01, 2020 - June 10, 2020	406	302 (74.40)	1.36	Adults	24.26	Online Survey	Weight gain	Guideline Score of the French National Nutrition and Health Program (PNNS-GS)
**Breiner et al., 2021**	USA	April 9, 2020 - May 27, 2020	159	144 (90.6)	11.68	Adults	na	Online Survey	Weight gain	Single item/ad hoc questionnaire
**Brito et al., 2021**	Brazil	the second half of May 2020	135	115 (85.9)	na	Adults	na	Online Survey	Weight gain	Single item/ad hoc questionnaire
**Buckland & Kemps, 2021**	Australia	August - September 2020	124	147 (98)	12.9	Adults	na	Online Survey	Snacking	Control of Eating Questionnaire (COEQ); Three Factor Eating Questionnaire (TFEQ)
**Buckland et al., 2021**	UK	May 15, 2020 - June 27, 2020	588	406 (69)	12.6	Adults	25.1	Online Survey	Snacking; Food cravings	Food Frequency Questionnaire (FFQ); Adult Eating Behaviour questionnaire (AEBQ)
**Buckley et al., 2021**	Australia	April 29, 2020 - May 7, 2020	204	175 (85.8)	8.1	Athletes	na	Online Survey	Body shape concern	Eating Attitudes Test-26 (EAT-26)
**Cardi et al., 2021**	Italy	April 6, 2020 - May 4, 2020	292	196 (67.1)	14.23	Adults	na	Online Survey	Overeating	Single item/ad hoc questionnaire
**Carroll et al., 2020**	Canada	April 20, 2020 -May 15, 2020	361	235 (65)	na	Parents	na	Online Survey	Snacking	Single item/ad hoc questionnaire
**Caso et al., 2022**	Italy	T1: April 30, 2020; T2: June 4, 2020	728	447 (61.4)	16.72	Students	na	Online Survey	Feeding/food restriction; Emotional eating; Overeating	Dutch Eating Behavior Questionnaire (DEBQ)
**Castellini et al., 2020**	Italy	T1: December 1, 2019; T2: January 15, 2020	97	97 (100)	10.89	Healthy Controls	na	Online Survey	Weight gain; Objective binge eating; Compensatory physical exercise	Eating Disorder Examination Questionnaire, (EDE-Q)

**Cecchetto et al., 2021**	Italy	May 14, 2020 - May 19, 2020	365	267 (73.1)	13.59	Adults	23.08	Online Survey	Emotional eating; Bingeing, Binge Eating	Dutch Eating Behaviour Questionnaire investigating Emotional Eating (DEBQ); Binge-Eating Disorder Screener (BEDS-7)
**Chan & Chiu, 2022**	China	March - April 2020	316	224 (70.9)	4.97	Young Adults	na	Online Survey	Eating Disorders	Chinese version of the SCOFF questionnaire
**Chee et al., 2020**	Canada	May-June 2020	680	510 (75)	14.7	Adults	25.5	Online Survey	Snacking	Beverage and Snack Questionnaire 2 (BSQ2)
**Cheikh Ismail et al., 2020**	Emirates	April-May 2020	1012	768 (75.9)	na	Adults	na	Online Survey	Snacking; Excessive physical activity; Weight Gain; Weight Loss; Feeding/food restriction, Undereating, Food avoidance, Appetite Loss	Single item/ad hoc questionnaire
**Cheick Ismail, Hashim, et al., 2021a**	Lebanon	June 3, 2020 - June 28, 2020	2507	1830 (73)	na	Adults	na	Online Survey	Snacking; Excessive physical activity; Weight Gain; Weight Loss	Short Food Frequency Questionnaire (FFQ); International Physical Activity Questionnaire Short Form (IPAQ-SF)
**Cheikh Ismail, Osaili, et al., 2021b**	Middle East and North Africa region	April 15, 2020 - April 29, 2020	2970	2126 (71.6)	na	Adults	na	Online Survey	Snacking; Excessive physical activity; Weight Gain; Weight Loss	Short Food Frequency Questionnaire (FFQ); International Physical Activity Questionnaire Short Form (IPAQ-SF)
**Chen et al., 2021**	China	April 2020	616	375 (60.9)	16.3	Adults	na	Telephone survey	Weight Gain	Single item/ad hoc questionnaire
**Cherick et al., 2020**	France	na	1092	na	na	Adults	na	Online Survey	Emotional Eating	Single item/ad hoc questionnaire
**Christensen et al., 2021**	USA (Kansas)	2 samples: December - March 2020 / April 2020	357	279 (78.2)	4.2	Students	25.0	Online Survey	Eating disorder symptoms	Eating Disorder Diagnostic Scale (EDDS)
**Cirillo et al., 2021**	Italy	April-May 2020	140	140 (100)	5	Women in fertility treatment	na	Online Survey	Emotional Eating	Single item/ad hoc questionnaire
**Coakley et al., 2021**	U.S.	September-October 2020	1243	909 (73.1)	na	Students	na	Online Survey	Anxiety and appetitive traits	Adult Eating Behavior Questionnaire (AEBQ)
**Constant et al., 2020**	France	April 2020	4005	2051 (51.2)	na	Adults	na	Online Survey	Physical Activity and snacking	Single item/ad hoc questionnaire
**Cooper et al., 2021**	USA	April-May 2020	1607	916 (57)	12,9	Adults	26	Online Survey	Perceived caloric intake, overall PA, vigorous PA, snacking	Single item/ad hoc questionnaire
**C. dos S. Costa et al., 2021**	Brasil	January 2020	14259	11168 (78,3)	na	Adults	na	Online Survey	Weight gain; Weight loss	Single item/ad hoc questionnaire
**Costa L. et al., 2021**	Brasil	December 2020 - January 2021	598	387 (64.7)	12.32	Adults	na	Online Survey	Emotional Eating; Body dissatisfaction	Three-factor eating questionnaire subscale (TFEQ-R21)

**Coulthard et al., 2021**	UK	na	620	546 (88)	na	Adults	na	Online Survey	Emotional Eating; Eating behaviour	Three-Factor Eating Questionnaire–Revised (TFEQ-R18)
**Cruceanu & Georgescu, 2021**	Romania	na	103	67 (65)	na	Adults	25	Online Survey	Physical Activity	Single item/ad hoc questionnaire
**Cummings, 2021**	USA	February 2019 - March 2020	868	449 (51.9)	12.86	Adults	25.99	Online Survey	Eating behaviours	Modified Yale Food Addiction Scale 2.0 (mYFAS2.0)
**Czepczor-Bernat et al., 2021**	Poland	December 2020 - January 2021	671	671 (100)	11.38	women	24.78	Online Survey	Eating disorder symptoms; Body image	Eating Disorder Inventory (EDI); Multidimensional Body–Self Relations Questionnaire (MBSRQ)
**Da Rocha et al., 2021**	Brasil	December 2019 - January 2021	29	29 (100)	5.6	Elderly Women	na	na	BMI; Body weight	Single item/ad hoc questionnaire
**De Pasquale et al., 2021**	Italy	March 2020 - February 2021	469	248 (52)	2.70	Students	na	Online Survey	Eating disorder symptoms; Binge eating	Eating Disorder Inventory-2 (EDI-2); Binge Eating Behaviors (BES)
**Deschasaux-Tanguy et al., 2021**	France	2009 - 2019	37252	19483 (52.3)	16.6	Adults	na	Online Survey	Weight change; Physical activity	Single item/ad hoc questionnaire
**Di Renzo et al., 2020**	Italy	April - May 2020	602	480 (79.7)	12.9	Adults	25	Online Survey	Eating disorder behaviours; Emotional Eating	Yale Food Addiction Scale
**Dicken et al., 2021**	UK	May - December 2020	1818	1267 (69.7)	14.3	Adults	26.2	Online Survey	Weight change; BMI	Single item/ad hoc questionnaire
**Dobrowolski & Włodarek, 2021**	Poland	na	183	143 (78)	11	Adults	na	Online Survey	Weight change; Physical activity	Single item/ad hoc questionnaire
**Đogaš, Kalcina et al., 2020**	Croatia	April - May 2020	3017	2121 (70.3)	na	Adults	24.64	Online Survey	Weight change	Single item/ad hoc questionnaire
**Dores et al., 2021**	Portugal/Spai n	April-May 2020	3161	2046 (65.2)	12.10	Adults	na	Online Survey	Exercise addiction	Exercise Addiction Inventory (EAI)
**Dor-Haim et al., 2021**	Israel	March-April 2020	1202	301 (25)	15.60	Adults	na	Online Survey	Weight gain	Single item/ad hoc questionnaire
**dos Santos Quaresma et al., 2021**	Brasil	April-May 2020	724	585 (80.8)	na	Adults	25	Online Survey	Emotional eating; Binge eating	Three-factor eating questionnaire (TFEQ-R21)
**Dragun et al., 2020**	Croatia	2018- May 2020	557	372 (73)	na	Students	22.0	Online Survey	Lifestyle and dietary habits	Single item/ad hoc questionnaire
**Drieskens et al., 2021**	Belgium	April 2020	28029	18919 (67.5)	na	Adults	na	Online Survey	Health behaviour; Weight gain	Single item/ad hoc questionnaire
**Drywień et al., 2020**	Poland	April-May 2020	1769	1769 (100)	na	Women	na	Online Survey	Weight change	Single item/ad hoc questionnaire
**Du et al., 2021**	China, Ireland,	April-May 2020	2254	1502 (66.7)	5.5	Students	24.4	Online Survey	Dietary habits	Single item/ad hoc questionnaire

	Malaysia, South Korea, Taiwan, the Netherlands, and the United States									
**Dun et al., 2021**	China	December2019 - May 2020	12889	10337 (80.2)	1	Students	na	Online Survey	Weight gain	Single item/ad hoc questionnaire
**Ekpanyaskul & Padungtod, 2021**	Thailand	May-June 2020	1011	617 (71)	9.82	Working-from-home workers	na	Online Survey	Dietary changes	Single item/ad hoc questionnaire
**Elangovan et al., 2020**	India	June-July 2020	1023	483 (47.2)	na	Workers (from home - at office)	na	Online Survey	Dietary changes	Single item/ad hoc questionnaire
**Elmacıoğlu et al., 2021**	Turkey	April-May 2020	1036	827 (80)	12.98	Adults	23.98	Online Survey	Emotional Eating; Weight gain	Three Factor Nutrition Questionnaire (TFEQ-R18)
**Enriquez-Martinez et al., 2021**	Brazil (N=2,171),Argentina (N=1,111), Peru (N=1,174), Mexico (N=686), and Spain (N=1,183)	April-September 2020	6325	4306 (68)	na	Adults	na	Online Survey	Dietary changes	Single item/ad hoc questionnaire
**Flaudias et al., 2020**	France	March 2020	5738	4280 (74.6)	4.5	Students	22.6	Online Survey	Eating disorders symptoms	Eating Disorder Inventory (EDI-2); Sick, Control, One, Fat, Food (SCOFF); Ideal Body Stereotype Scale (IBSS)
**Freitas et al., 2021**	Brazil	T0: March-December 2019; T1: August 2020	71	71 (100)	0.41	Young Women (students)	22.93	T0: Paper-pencil Survey, T1: Online Survey	Food cravings; Emotional eating; Overeating	Brazilian Food Craving Inventory (FCI-Br); Three Factor Eating Questionnaire—R21 (TFEQ-R21)
**Gao et al., 2021**	China	February 17, 2020 - February 27, 2020	912	723 (79.3)	10.48	Adults	na9	Online Survey	Emotional Eating	Adult Eating Behavior Questionnaire (AEBQ)
**Grant et al., 2021**	Italy	April 22. 2020 - May 10, 2020	2678	1387 (51.8)	na	Adults	na	Online Survey	Emotional Eating; Snacking; Excessive physical activity; Weight Gain	Single item/ad hoc questionnaire
**Guerrini Usubini et al., 2021**	Italy	December 1, 2020 - January 31, 2021	437	224 (51.3)	5.12	Young adults	21.9	Online Survey	Emotional Eating	The Emotional Eating subscale of the Dutch Eating Behavior Ques tionnaire (EE_DEBQ)

**Jackson et al., 2022**	USA	April 21, 2020 - May 06, 2020	360	184 (51.1)	16.17	Adults	na	Online Survey	Snacking	Single item/ad hoc questionnaire
**Jackson et al., 2021**	USA	April 21, 2020 -May 6, 2020	360	184 (51.1)	16.2	Adults	26.26	Online Survey	Weight Gain	Intuitive Eating Scale (IES-2)
**Jordan et al., 2021**	USA	July–September 2020	140	124 (88.6)	6.85	Adults	29.10	Online Survey	Concern about weight gain; Disordered eating; Emotional eating	Eating Disorder Examination Questionnaire-Short Form (EDE-QS); 12 items from the Emotional Eating Scale-Revised (EES-R)
**Karakose et al., 2021**	Turkey	2020-2021	266	35 (13.2)	na	Adults	na	Online Survey	External eating behavior; Restrained eating behavior	Fear of COVID-19 Scale; Dutch Eating Behavior Questionnaire" (DEBQ)
**Kaufman-Shriqui et al., 2022**	Israel	March 30, 2020 -April 25, 2020	3797	2848 (75)	na	Adults	na	Online Survey	Diet quality	Single item/ad hoc questionnaire
**Kesilmi et al., 2021**	Turkey		286	na (na)	4.72	Adults	71.8	Online Survey	Uncontrolled Eating; Emotional Eating	International Physical Activity Scale (IPAQ); Three-Factor Eating Questionnaire (TFEQ)
**Khubchandani et al., 2020**	USA	April 2020	838	433 (52)	0.39	Adults	na	Online Survey	Eating habits	Single item/ad hoc questionnaire
**Landaeta-Díaz et al., 2021**	Chile	April 1, 2020- May 8, 2020	1724	1420 (82.3)	10.3	Adults	na	Online Survey	Food consumption; Body weight	Single item/ad hoc questionnaire
**León-Paucar et al., 2021**	Peru	June 26, 2020 – July 27, 2020	589	292 (49.6)	9.4	Adults	na	Online Survey	Body Dissatisfaction	Food frequency questionnaire (FFQ); Body Shape Questionnaire (BSQ)
**Liboredo et al. 2021**	Brasil	August-September 2020	1368	1094 (80)	na	Adults	na	Online Survey	Eating disorder symptoms	Three-Factor Eating Questionnaire (TFEQ-R21)
**Li et al., 2021**	China	September 2019, February 2020, and April 2020	634	443 (70)	1.56	Adults	na	Online Survey	Disinhibited eating	Eating Questionnaire-R18 (TFEQ-R18)
**Lofrano-Prado et al., 2021**	Brazil	May 5, 2020 - May 17, 2020	1854	1085 (58.5)	13.1	Adults	25.7	Online Survey	BMI; Physical activity	Single item/ad hoc questionnaire
**Ma et al., 2021**	China	April 25, 2020 - May 11, 2020	10545	5940 (56.3)	9.9	Adults	na	Online Survey	Weight Changes	Single item/ad hoc questionnaire
**Madalı et al., 2021**	Turkey	August-September 2020.	1626	1131 (69.6)	11	Adults	24.4	Online Survey	Emotional Eating	Emotional Eating Scale
**Madan et al., 2021**	India		1000	500 (50)	na	Adults	na	Online Survey	Weight Changes	Single item/ad hoc questionnaire
**Maffoni et al., 2021**	Italy	April 30, 2020- May 10, 2020	1304	973 (75)	na	Adults	23.2	Online Survey	Craving or eating between meals	Single item/ad hoc questionnaire

**Mahar et al., 2021**	Pakistan	April-May 2020	313	na (na)	na	Adults	na	Online Survey	Eating disorders; Binge eating	SCOFF questionnaire; BEDS-7
**Malkawi et al., 2021**	Jordan	March-April 2020.	2103	2103 (100)	6.4	Adults	na	Online Survey	Weight Changes	Single item/ad hoc questionnaire
**Martínez-de-Quel et al., 2021**	Spain	March 16 and March 31, 2020 - April 30 and May 11, 2020	161	60 (37)	11.2	Adults	23.7	Online Survey	Eating disorders	Eating Attitude Test-26 (EAT-26)
**Mason et al. 2021**	California	October 24, 2018 and October 31, 2019 and May 18, 2020 and July 21, 2020	1820	1119 (61)	.46	Adults	na	Online Survey	Overeating; Snacking	Single item/ad hoc questionnaire
**Mazzolani et al., 2021**	Brazil	June - September 2020	1183	1183 (100)	0.71	Women	24.79	Online Survey	Snacking; Feeling fat, Body shape concern, Body dissatisfaction, Body misperception; Bingeing, Binge Eating	Brazilian-Portuguese version of The Eating Motivation Survey (TEMS); Binge Eating Scale (BES); Disordered Eating Attitude Scale- Short Version
**McAtamney et al., 2021**	United Kingdom	Mid July 2020	136	88 (64.7)	11.88	Adults	26.21	Online Survey	Emotional eating	Emotional Eating Scale (EES); Salzburg Emotional Eating Scale (SEES)
**Micheletti Cremasco et al., 2021**	Italy	May 14, 2020 – May 31, 2020	3666	2676 (73)	12	Adults	22.3	Online Survey	Weight changes	Single item/ad hoc questionnaire
**Molina-Montes et al., 2021**	Spain	March 20, 2020 - May 5, 2020	36185	28064 (77.6)	na	Adults	na	Online Survey	Snacking	Mediterranean Diet Adherence Screener (MEDAS)
**Mota et al., 2021**	Brazil	May - July 2020	710	574 (80.8)	na	Healthcare Professionals	na	Online Survey	Bingeing, Binge Eating; Night eating, Night feeding	Single item/ad hoc questionnaire
**Mulugeta et al., 2021**	Massachusetts.	March 01, 2020 - May 31, 2020	11534	7681 (66.6)	na	Adults	na	Electronic Medical Records	Weight changes	Medical records
**Mumena, 2020**	Saudi Arabia	April 13 and 22, 2020	879	576 (65.5)	12.1	Adults	na	Online Survey	Changes in eating habits	Single item/ad hoc questionnaire
**Nitu et al., 2021**	Romania	January-March 2021	620	501 (80.8)	9.42	Adults	22.93	Online Survey	Weight fluctuations; Meal frequency	Single item/ad hoc questionnaire
**Özcan & Yeşİlkaya, 2021**	Turkey	na	578	422 (73)	na	Adults	24.12	Online Survey	Emotional eating	Turkish version of Emotional Eater Questionnaire (EEQ-TR)
**Özden & Parlar Kiliç, 2021**	Turkey	May 15 and 29, 2020	1011	607 (60)	3.11	Adults	na	Online Survey	Weight change; Addictive eating behavior	Nutrition Exercise Behavior Scale

**Özen et al., 2021**	Turkey.	April-May 2020	334	162 (48.5)	3.7	Adults	22.3	na	Emotional Eating	The Emotional Eating Scale (EES)
**O¨ zenog˘lu et al., 2021**	Turkey.	April 2020	432	312 (72.2)	12.14	Adults	na	na	Attitude of Healthy Eating levels	Healthy Nutrition Attitude Scale (ASHN)
**Pak et al., 2022**	Turkey.	April 15, 2020 - May 15, 2020	362	242 (66.9)	8.22	Adults	na	Online Survey	Emotional Eating	Eating Questionnaire-R21 (TFEQ-R21)
**Palmer et al., 2021**	Germany	March 12, 2020 - May 3, 2020	827	622 (75.2)	na	Adults	na	Online Survey	Weight change	Single item/ad hoc questionnaire
**Pappa et al., 2021**	West London	June-July 2020	387	275 (71.1)	na	Adults	na	Online Survey	Overeating	Single item/ad hoc questionnaire
**Pertuz-Cruz et al., 2021**	Spain	April 6, 2020 - May 22, 2020	2745	2006 (73.1)	na	Adults	na	Online Survey	Snacking; Overeating; Weight gain	Single item/ad hoc questionnaire
**Phillipou et al., 2020**	Australia	April 20	5289	4231 (80)	13.67	Adults	na	Online Survey	Food restricting; Binge eating	Eating Disorders Examination Questionnaire (EDE-Q).
**Phillipou et al., 2021**	Australia	April-September 2020	4684	na (na)	14.17	Adults	na	Online Survey	Changes in eating habits	Eating Disorders Examination Questionnaire (EDE-Q).
**Pirutinsky et al., 2021**	New Yourk	March 30, 2020 - May 14, 2020	731	482 (66)	16.79	Adults	na	Online Survey	Weight Change	Single item/ad hoc questionnaire
**Pisot et al., 2020**	Slovenia	April 15, 2020 - May 3, 2020	4108	2567 (62.5)	13.2	Adults	na	Online Survey	Snacking; Weight gain	Single item/ad hoc questionnaire
**Poelman et al., 2021**	Netherlands	April 22, 2020– April 28, 2020	1030	520 (50.5)	17.0	Adults	26.2	Online Survey	Changes in eating habits	Single item/ad hoc questionnaire
**Pompili et al., 2022**	Italy	April-May, 2020	447	280 (63)	1.93	Adults	22.65	Online Survey	Food disturbance; Compensatory behaviors	Single item/ad hoc questionnaire
**Pop et al., 2021**	Romania	2018-2020	88	0 (0)	0.67	Adults	24.2	na	Weight gain	Single item/ad hoc questionnaire
**Prezotti et al., 2021**	Brazil	June 11, 2020 - June 19, 2020	275	26 (9.5)	2.74	Urology residents	na	Online Survey	Weight Gain; Weight loss	Single item/ad hoc questionnaire
**Puhl et al., 2020**	USA	T0: 2018 T1: Aprile - July 2020	584	375 (64.2)	2	Young Adults	28.2	Online Survey	Bingeing, Binge Eating; Emotional eating	five-item coping subscale of the Motivations to Eat Scale; two questions adapted from the adult version of the Questionnaire on Eating and Weight Patterns-Revised
**Queiroz et al., 2021**	Brazil	April 30, 2021 - May 31, 2021	302	232 (76.8)	na	Adults	na	Online Survey	Weight gain; Weight loss	ecSI2.0™BR questionnaire
**Radwan et al., 2021**	United Arab Emirates	May 5, 2020 - May 18, 2020	2060	1548 (75.1)	10.05	Adults	27.33	Online Survey	Weight Gain; Weight loss; Snacking; Overeating	Single item/ad hoc questionnaire

**Ramalho et al., 2022**	Portugal	May 11, 2020 - May 25, 2020	254	210 (82.7)	11.82	Adults	24.08	Online Survey	Overweight, Weight Gain; Weight loss; Bingeing, Binge Eating; Emotional eating; Feeling fat, Body shape concern, Body dissatisfaction, Body misperception; Overeating; Feeding/food restriction, Undereating, Food avoidance, Appetite Loss	Three-factor eating questionnaire (TFEQ-R21)
**Reyes-Olavarría et al., 2020**	Chile	May-June 2020	700	528 (75.4)	na	Adults	na	Online Survey	Overweight, Weight Gain; Overeating; Undereating	Single item/ad hoc questionnaire
**Robertson et al., 2021**	UK	May 11, 2020 - June 26, 2020	264	206 (78)	14.16	Adults	na	Online Survey	Body shape concern; Excessive physical activity, excessive exercise, compulsive exercise	Single item/ad hoc questionnaire
**Robinson et al., 2021**	UK	April 28, 2020 - May 2, 2020	2002	1236 (61.7)	12.3	Adults	27.8	Online Survey	Weight changes;,Physical Activity, Diet quality; Binge eating	IPAQ; SFFQ; ALEBS
**Robinson et al., 2020**	UK	April 19-22, 2020	723	488 (67)	9.6	Adults	na	Online Survey	Bingeing; Physical activity	IPAQ; SFFQ; ALEBS
**Rodriguez-Perez et al., 2020**	Spain	March 20, 2020 -Mid April 2020	7514	5305 (70.6)	na	Adults	na	Online Survey	Changes in dietary habits	Single item/ad hoc questionnaire
**Rogers et al., 2021**	USA	T0: March 30, 2020 - April 7, 2020 T1: November 2-21, 2020	619	342 (55)	na	Adults	na	Online Survey	Changes in dietary habits; Changes in exercise habits; Risk for food insecurity	Diet Behavior and Nutrition questionnaire of the National Health and Nutrition Examination Survey (NHANES); PROMIS-29 (PROPr);
**Ruiz-Zaldibar et al., 2022**	Spain	May 28, 2020 – June 21, 2020	675	472 (69.9)	12.9	Adults	24.2	Online Survey	Emotional eating	Emotional eater questionnaire (EEQ)
**Ruiz et al., 2021**	Cross-cultural: United Kingdom, South Korea, Finland, Philippines, Latin America, Spain, North America, Italy	May-August 2020	1131	745 (65)	12.88	Adults	na	Online Survey	Changes in eating habits; Weight change	Single item/ad hoc questionnaire
**Sadler et al., 2021**	USA	May-June 2020	428	270 (63.1)	8.25	Adults	27.9	Online Survey	Food intake	Emotional overeating subscale from the Adult Eating Behavior Questionnaire
**Sánchez et al., 2021**	Spain	May 26, 2020-June 10, 2020	1000	515 (51.5)	18	Adults	25.3	Computer-assisted telephone	Weight change	CATI

								interviews (CATI)		
**Sánchez-Sánchez et al., 2020**	Spain	May 2020	1065	775 (72.8)	12.4	Young Adults	na	Online Survey	Physical activity; Weight change	Single item/ad hoc questionnaire
**Sánchez-Sánchez et al., 2021**	Spain	January 10, 2021 - February 10, 2021	637	477 (74.9)	na	Adults	na	Online Survey	Increase in food consumption	Single item/ad hoc questionnaire
**Santana et al., 2021**	Brazil	April-May 2020	955	735 (77)	8.07	Students	na	Online Survey	Changes in dietary habits	Food Frequency Questionnaire
**Sarda et al., 2022**	France	June 2020	2422	1269 (52.4)	na	Adults	na	Online Survey	Changes in cooking habits	Single item/ad hoc questionnaire
**Saxena et al., 2021**	India	June-July 2020	60	30 (50)	1.35	Students	23.06	Online Survey	Food intake	Single item/ad hoc questionnaire
**Scacchi et al., 2021**	Italy	May 2020	1865	1304 (69.9)	16	Adults	na	Online Survey	Changes in food choice and intake	Emotional Overeating Questionnaire-5 (EOQ-5)
**Scarmozzino & Visioli, 2020**	Italy	April 2020	1929	1319 (67)	na	Adults	na	Online Survey	Changes in dietary habits	Single item/ad hoc questionnaire
**Scharmer et al., 2020**	USA	March-April 2020	295	192 (65.1)	2.0	Students	na	Online Survey	Eating disorder symptoms	Eating Disorder Examination-Questionnaire (EDE-Q); Compulsive exercise test (CET)
**Schulte et al., 2022**	USA	February 2021	243	na (na)	13.19	Adults	25.85	Online Survey	Changes in weight and eating behaviours	Modified Yale Food Addiction Scale 2.0 (mYFAS2.0)
**Seal et al., 2022**	USA	T0: March 2020, T1: August 2020	1516	1193 (78,8)	17.6	Adults	27.1	Online Survey	Overweight, Weight Gain	Single item/ad hoc questionnaire
**Sebastião et al., 2022**	USA & Brazil	September-October 2020	277	186 (67.1)	13.6	Adults	27.1	Online Survey	Weight Gain	Single item/ad hoc questionnaire
**Serin et al., 2020**	Turkey	na	1064	624 (58.6)	na	Students	na	Online Survey	Feeding/food restriction, Undereating, Food avoidance, Appetite Loss; Emotional eating	Dutch Eating Behaviour Questionnaire (DEBQ)
**Shaun et al., 2021**	Bangladesh	February 3-13, 2021	394	175 (44.42)	na	Students	na	Online Survey	Weight Gain; Undereating; Excessive physical activity; Snacking	International Physical Activity Questionnaire Short Form (IPAQ-SF)
**Shibata et al., 2021**	Brazil, Italy, Spain, Lithuania, Portugal, UK, Japan, Hungay	April - June 2020	2295	1607 (70)	11.40	Adults	na	Online Survey	Feeling fat, Body shape concern, Body dissatisfaction, Body misperception; Excessive physical activity	Appearance Anxiety Inventory; Excessive Addiction Inventory (EAI)
**Shin, 2021**	USA	na	515	306 (59.4)	12.5	Overweight and Obese (BMI>25)	31.2	Online Survey	Weight Gain	Single item/ad hoc questionnaire

**Sidor et al., 2020**	Poland	April 2020	1097	1043 (95.1)	9	Adults, non working on regular basis	23.5	Online Survey	Weight Gain; Weight loss; Overeating; Snacking	Single item/ad hoc questionnaire
**Silva et al., 2021**	Portugal	April 2020	5856	2495 (42.6)	na	Adults	na	Telephone Survey	Weight Gain; Snacking	Single item/ad hoc questionnaire
**Silverman & Wang, 2021**	US	June-July 2020	129	116 (90)	na	School teachers	na	Online Survey	Weight gain	Dutch Eating Behavioral Questionnaire; Food Frequency Questionnaire short-form
**Skotnicka et al., 2021**	Poland, Austria and the United Kingdom	October 1-30, 2020	1831	604 (56.4)	na	Adults	na	Online Survey	Weight change	Single item/ad hoc questionnaire
**Smith et al., 2021**	USA	May - June 2020	429	272 (63.4)	na	Adults	na	Online Survey	Physical Activity	Single item/ad hoc questionnaire
**Sobba et al., 2021**	USA	August 26, 2020 - October 21, 2020	589	435 (73.9)	na	Adults	27.36	Online Survey	Snacking; Physical Activity; Weight change	Single item/ad hoc questionnaire
**Solè et al., 2021**	Spain	May 14, 2020 - June 8, 2020	413	306 (75.7)	14.04	Adults (communit y controls)	na	Online Survey	Weight Gain; Weight loss	Single item/ad hoc questionnaire
**Suka et al., 2021**	Japan	November 2020	8000	na (na)	na	Adults	na	Online Survey	Excessive physical activity; Weight Gain; Weight Loss; Night eating	National Health and Nutrition Survey
**Sulejmani et al., 2021**	Kosovo	May- June 2020	689	488 (71)	na	Adults	na	Online Survey	Weight Gain; Snacking; Overeating	Single item/ad hoc questionnaire
**Swami et al., 2021**	UK	May 21, 2020	506	255 (50.4)	11.36	Adults	26.35	Online Survey	Feeling fat, Body shape concern, Body dissatisfaction, Body misperception	Women: Body Dissatisfaction Subscale (EDI-3-BD) and Drive for Thinness subscale (EDI-3-DT) of EDI-3; Men: Low Body Fat subscale and Muscolarity subscale of Male Body Attutude Scale (MBAS)
**Tabler et al., 2021**	USA (Oklahoma, Wyoming, Texas)	October 2020 - January 2021	411	304 (74)	11.4	Adults	26.7	Online Survey	Weight Gain; Weight loss; Eating disorder symptoms	Eating Disorder Examination—Questionnaire Short (EDE-QS)
**Tan et al., 2021**	Malaysia	June 4-11, 2021-	1013	652 (64.4)	2.46	Young Adults	22.78	Online Survey	Overweight, Weight Gain; Weight loss	Food Choice Questionnaire (FCQ)
**Tfifha et al., 2021**	Tunisia	April 19, 2020 - May 5, 2020	180	126 (70.2)	na	Young doctors	na	Online Survey	Bingeing, Binge Eating	Single item/ad hoc questionnaire
**Thahir et al., 2021**	Indonesia	April 4, 2020 - April 18, 2020	1044	862 (82.6)	2.42	Students	21.94	Online Survey	Overweight, Weight Gain	Single item/ad hoc questionnaire
**Trott et al., 2021**	UK	T0: April 08, 2019 - 31 July 2019 T1:	319	268 (84)	11.75	Adults (health club users)	24.02	Online Survey	Excessive physical activity, excessive exercise, compulsive exercise; Feeling fat, Body shape concern, Body	Exercise Addiction Inventory (EAI) - Eating Attitudes Test 26 (EAT-26) - Body

		August 26, 2020 - Semptember 11, 2020							dissatisfaction, Body misperception	Dysmorphic Disorder Questionnaire (BDDQ)
**Turgut et al., 2020**	Turkey	na	278	111 (39.93)	5.74	Athletes	22.52	na	Night eating, Night feeding	Night Eating Questionnaire (NE)
**Urzeala et al., 2022**	France, Australia, Austria, Canada, Chile, China, Denmark, Indonesia, Italy, Iran, Norway, Portugal, Tunisia, Taiwan, Scotland, Switzerland, Romania and United States	March - June 2020	10121	5603 (55.36)	na	Adults	24.78	Online Survey	Weight Gain	COVISTRESS Questionnaire
**Vacca et al., 2021**	Italy - Spain	April 2020	817	536 (65.61)	na	Adults	na	Online Survey	Emotional eating; Overeating; Food restriction	Three-factor eating questionnaire (TFEQ)-R21
**Valencia et al., 2021**	Arizona	na	155	na	na	Adults	na	na	Overeating	Single item/ad hoc questionnaire
**Vidal et al., 2021**	Uruguay	May 2020	891	659 (74)	na	Adults	na	Online Survey	Snacking	Single item/ad hoc questionnaire
**Visser et al., 2020**	The Netherlands	June-October 2020	1119	591 (52.8)	7	Elders	na	Online Survey	Weight Gain, Increased Physical activity, Snacking	Single item/ad hoc questionnaire
**Wang et al., 2022**	US	May-June 2020	197	197 (100)	6.87	Mothers	28.34	Online Survey	Emotional Eating	TFEQ-R18, FPSQ
**Yang et al., 2020**	China	May 2020	10082	7230 (71.7)	2.3	Adults	21.8	Online Survey	Weight Gain	Single item/ad hoc questionnaire
**Yılmaz Akyüz et al., 2021**	Turkey	April - May 2020	2019	1589 (78.2)	na	Adults	na	Online Survey	Emotional Eating; Overweight, Weight Gain; Weight Loss	Three-Factor Eating Scale (TFEQTr21)
**Yilmaz & Sanlier, 2021**	Turkey	April-May 2020	529	529 (100)	8	Women	na	Online Survey	Night eating	Night Eating Syndrome Questionnaire
**Yokoro & Wakimoto, 2021**	Japan	May 2020	164	164 (100)	0.7	Students	20.8	Online Survey	Weight change; Snacking	Single item/ad hoc questionnaire
**Zach & Fernandez, 2021**	Israeli	April 2020	1855	1289 (69.49)	14.5	Adults	25.46	Online Survey	Weight change	Single item/ad hoc questionnaire
**Zachary et al., 2020**	USA	2020	173	96 (55.49)	12.5	Adults	27.0	Online Survey	Weight gain	Single item/ad hoc questionnaire
**Zhang & Zhang, 2020**	China	April-May 2020	640	100 (100)	4.5	Pregnant Women	na	Online Survey	Emotional eating	Dutch Eating Behavior Questionnaire

**Zhou & Wade, 2021**	USA	September 2021	100	100 (100)	2.01	Students at risk for disordered eating	25.52	Online Survey	Eating Disorder	EDE-Q
**Zhu et al., 2021**	China	March-April 2020	889	542 (61)	11.4	Adults	na	Online Survey	Snacking; Physical Activity; Weight change	Single item/ad hoc questionnaire
**Zielinska & Luszczki, 2021**	Poland	January-June 2021	1022	958 (93.7)	11.86	Adults	25.20	Online Survey	Weight gain; Weight loss; Food addiction	Yale Food Addiction Scale (YFAS-2)

Note: *na: not applicable*

Supplementary Table S4

Meta-regression of factor affecting prevalence of Weight Gain

Weight Gain	EST.	SE	95% CI	p
Risk of bias				
High risk (ref) (k=8)				
Medium risk (k=31)	0.0004	0.026	-0.051; 0.052	0.988
Low risk (k=45)	-0.067	0.043	-0.151; 0.018	0.122
Recruitment time				
First wave (ref) (k=66)				
Second wave (k=8)	-0.012	0.044	-0.097; 0.074	0.787
Third wave (k=8)	-0.014	0.043	-0.098; 0.071	0.750
Country				
Asia (ref) (k=35)				
Africa (k=3)	0.082	0.066	-0.047; 0.211	0.214
Europa (k=28)	-0.048	0.028	-0.102; 0.006	0.082
South America (k=11)	0.057	0.038	-0.017; 0.131	0.133
North America (k=7)	0.032	0.046	-0.058; 0.122	0.491
Type of sample				
General adult population (ref) (k=69)				
Women (k=3)	-0.007	0.069	-0.142; 0.128	0.920
Athletes (k=8)	0.106	0.119	-0.126; 0.338	0.371
Older adults (k<3)	na	na	na	na
Overweight & Obese (k<3)	na	na	na	na
Students (k=8)	0.004	0.044	-0.082; 0.090	0.927
% Female (k=79)	0.0002	0.0007	-0.001; 0.002	0.715
Age (k=53)	-0.0008	0.001	-0.003; 0.002	0.474
BMI (k=38)	0.003	0.007	-0.012; 0.017	0.704
Outcome assessment				
Single item (ref) (k=52)				
Standardized questionnaire (k=31)	-0.025	0.026	-0.075; 0.026	0.338

Note: *na: not applicable, SE: standard error, 95% CI: 95% confidence interval; ref: reference category; the reference category was defined as the most numerous category; significant predictors in bold.*

Supplementary Table S5

Metaregression of factor affecting prevalence of Food restriction

Food restriction	EST.	S.E.	95% CI of Est.	P value
Risk of bias				
High risk (ref) (k<3)				
Medium risk (k<3)	na	na	na	na
Low risk (k=9)	0.225	0.188	-0.143; 0.594	0.231
Recruitment time				
First wave (ref) (k=12)				
Second wave (k<3)	na	na	na	na
Country				
Asia (ref) (k=5)				
Europa (k<3)	na	na	na	na
South America (k<3)	na	na	na	na
North America (k=5)	0.120	0.163	-0.200; 0.439	0.463
Type of sample				
General adult population (ref) (k=12)				
Students (k<3)	na	na	na	na
% Female (k=13)	0.014	0.004	0.005; 0.022	**0.001**
Age (k=9)	-0.007	0.016	-0.039; 0.025	0.682
BMI (k=6)	0.017	0.071	-0.123; 0.156	0.815
Outcome assessment				
Single item (ref) (k=7)				
Standardized questionnaire (k=6)	0128	0.130	-0.127; 0.382	0.325

Note: *na: not applicable, SE: standard error, 95% CI: 95% confidence interval; ref: reference category; the reference category was defined as the most numerous category; significant predictors in bold.*

Supplementary Table S6

Metaregression of factor affecting prevalence of Body shape concerns

Body shape concerns	EST.	S.E.	95% CI of Est.	P value
Risk of bias				
High risk (ref) (k= 4)				
Medium risk (k= 3)	0.097	0.175	-0.246; 0.439	0.580
Low risk (k=<<3)	na	na	na	na
Recruitment time				
First wave (ref) (k=6)				
Second wave (k=<<3)	na	na	na	na
Country				
Australia (ref) (k=<<3)				
Asia (k=<<3)	na	na	na	na
Europa (k=<<3)	na	na	na	na
South America (k=<<3)	na	na	na	na
North America (k= 3)	0.240	0.300	-0.834; 0.792	0.496
Type of sample				
General adult population (ref) (k=6)				
Women (k=>3)	na	na	na	na
Athletes (k=>3)	na	na	na	na
% Female (k=8)	-0.003	.004	-0.011; 0.006	0.515
Age (k=7)	0.009	0.008	-0.008; 0.025	0.305
BMI (k=3)	-0.035	0.057	-0.146; 0.076	0.537
Outcome assessment				
Single item (ref) (k=3)				
Standardized questionnaire (k=5)	-0.244	0.122	-0.483; -0.006	**0.044**

Note: *na: not applicable, SE: standard error, 95% CI: 95% confidence interval; ref: reference category; the reference category was defined as the most numerous category; significant predictors in bold.*

Supplementary Table S7

Metaregression of factor affecting prevalence of Weight loss

Weight loss	EST.	S.E.	95% CI of Est.	P value
Risk of bias				
High risk (ref) (k=24)				
Medium risk (k= 18)	-0.086	0.041	-0.167; -0.005	**0.037**
Low risk (k=3)	-0.040	0.021	-0.081; 0.001	0.058
Recruitment time				
First wave (ref) (k=35)				
Second wave (k=4)	-0.030	0.037	-0.103; 0.043	0.417
Third wave (k=5)	0.048	0.034	-0.018; 0.114	0.157
Country				
Asia (ref) (k=19)				
Africa (k=3)	0.022	0.045	-0.066; 0.109	0.627
Europa (k=12)	0.037	0.026	-0.014; 0.089	0.158
South America (k=6)	-0.013	0.033	-0.078; 0.053	0.705
North America (k=5)	0.007	0.036	-0.064; 0.079	0.837
Type of sample				
General adult population (ref) (k=41)				
Healthcare professionals (k<3)	na	na	na	na
Students (k=3)	0.063	0.043	-0.021; 0.147	0.142
% Female (k=43)	0.000	0.000	0.000; 0.000	**0.031**
Age (k=27)	-0.003	0.001	-0.006; -<0.001	**0.017**
BMI (k=22)	-0.006	0.007	-0.020; 0.007	0.448
Outcome assessment				
Single item (ref) (k=26)				
Standardized questionnaire (k=19)	0.009	0.021	-0.033; 0.051	0.684

Note: *na: not applicable, SE: standard error, 95% CI: 95% confidence interval; ref: reference category; the reference category was defined as the most numerous category; significant predictors in bold.*

Supplementary Table S8

Metaregression of factor affecting prevalence of Excessive physical activity

Excessive physical activity	EST.	S.E.	95% CI of Est.	P value
Risk of bias				
High risk (ref) (k=13)				
Medium risk (k=16)	0.003	0.063	-0.120; 0.126	0.959
Low risk (k=5)	0.112	0.089	-0.062; 0.286	0.208
Recruitment time				
First wave (ref) (k=26)				
Second wave (k=3)	0.304	0.093	0.123; 0.486	**<0.001**
Third wave (k=<3)	na	na	na	na
Country				
Asia (ref) (k=13)				
Europa (k=11)	0.007	0.048	-0.086; 0.101	0.876
South America (k<3)	na	na	na	na
North America (k= 8)	0.290	0.053	0.187; 0.393	**<.001**
Type of sample				
General adult population (ref) (k=31)				
Healthcare professionals (k<3)	na	na	na	na
Students (k<3)	na	na	na	na
% Female (k=32)	0.006	0.003	.000; 0.011	**0.034**
Age (k=15)	-0.004	0.005	-0.013; -0.006	0.439
BMI (k=8)	0.029	0.030	-0.030; 0.088	0.335
Outcome assessment				
Single item (ref) (k=20)				
Standardized questionnaire (k=14)	0.031	0.059	-0.085; 0.146	0.602

Note: *na: not applicable, SE: standard error, 95% CI: 95% confidence interval; ref: reference category; the reference category was defined as the most numerous category; significant predictors in bold.*

Supplementary Table S9

Metaregression of factor affecting prevalence of Bingeing

Bingeing	EST.	S.E.	95% CI of Est.	P value
Risk of bias				
High risk (ref) (k=5)				
Medium risk (k=3)	0.035	0.198	-0.352; 0.422	0.858
Low risk (k<3)	na	na	na	na
Recruitment time				
First wave (ref) (k=7)				
Second wave (k<3)	na	na	na	na
Country				
Asia (ref) (k=2)				
Africa (k<3)	na	na	na	na
Europa (k<3)	na	na	na	na
South America (k<3)	na	na	na	na
North America (k=3)	0.210	0.152	-0.088; 0.507	0.168
Type of sample				
General adult population (ref) (k=6)				
Healthcare professionals (k=3)	-0.155	0.166	-0.481; 0.171	0.352
Students (k<3)	na	na	na	na
% Female (k=10)	-0.003	0.004	-0.012; 0.005	0.485
Age (k=7)	<0.000	0.014	-0.026; 0.027	0.995
BMI (k<3)	na	na	na	na
Outcome assessment				
Single item (ref) (k= 6)				
Standardized questionnaire (k=4)	-0.006	0.165	-0.328; 0.317	0.973

Note: *na: not applicable, SE: standard error, 95% CI: 95% confidence interval; ref: reference category; the reference category was defined as the most numerous category; significant predictors in bold.*

Supplementary Table S10

Metaregression of factor affecting prevalence of Overeating

Overeating	EST.	S.E.	95% CI of Est.	P value
Risk of bias				
High risk (ref) (k=16)				
Medium risk (k=10)	-0.039	0.086	-0.208; 0.130	0.652
Low risk (k<3)	na	na	na	na
Recruitment time				
First wave (ref) (k=27)				
Third wave (k<3)	na	na	na	na
Country				
Asia (ref) (k= 9)				
Europa (k=11)	0.071	0.096	-0.117; 0.258	0.461
South America (k=3)	0.172	0.142	-0.106; 0.450	0.225
North America (k=5)	0.015	0.119	-0.218; 0.248	0.899
Type of sample				
General adult population (ref) (k=26)				
Students (k<3)	na	na	na	na
% Female (k=28)	<0.000	<0.000	-0.000; 0.001	0.734
Age (k=16)	-0.004	0.007	-0.018; 0.011	0.632
BMI (k=10)	-0.040	0.048	-0.135; 0.054	0.405
Outcome assessment				
Single item (ref) (k=18)				
Standardized questionnaire (k=10)	0.191	0.074	0.045; 0.337	**0.010**

Note: *na: not applicable, SE: standard error, 95% CI: 95% confidence interval; ref: reference category; the reference category was defined as the most numerous category; significant predictors in bold.*

Supplementary Table S11

Metaregression of factor affecting prevalence of Food craving

Food craving	EST.	S.E.	95% CI of Est.	P value
Risk of bias				
High risk (ref) (k= 1)				
Medium risk (k= 3)	-0.422	0.172	-0.760; -0.084	**0.014**
Recruitment time				
First wave (ref) (k=3)				
Third wave (k<3)	na	na	na	na
Country				
Europa (k=3)				
South America (k<3)	na	na	na	na
Type of sample				
General adult population (ref) (k=3)				
Healthcare professionals (k<3)	na	na	na	na
% Female (k=4)	-0.010	0.005	-0.020; <0.000	0.063
Age (k=4)	0.003	0.019	-0.034; 0.040	0.873
BMI (k=3)	1.009	1.038	-1.025; 3.042	0.331
Outcome assessment				
Single item (ref) (k=3)				
Standardized questionnaire (k=1)	na	na	na	na

Note: *na: not applicable, SE: standard error, 95% CI: 95% confidence interval; ref: reference category; the reference category was defined as the most numerous category; significant predictors in bold.*

Supplementary Table S12

Metaregression of factor affecting prevalence of Snacking

Snacking	EST.	S.E.	95% CI of Est.	P value
Risk of bias				
High risk (ref) (k= 5)				
Medium risk (k= 3)	0.060	0.055	-0.048; 0.168	0.277
Low risk (k<3)	na	na	na	na
Recruitment time				
First wave (ref) (k=7)				
Second wave (k<3)	na	na	na	na
Country				
Australia (ref) (k<3)				
Asia (k=14)	-0.278	0.186	-0.642; 0.086	0.134
Europa (k= 18)	-0.252	0.184	-0.614; 0.109	0.171
South America (k=3)	-0.180	0.206	-0.584; 0.224	0.384
North America (k=8)	-0.175	0.190	-0.547; 0.198	0.358
Type of sample				
General adult population (ref) (k=40)				
Older adults (k<3)	na	na	na	na
Students (k=3)	-0.012	0.108	-0.223; 0.199	0.913
% Female (k=43)	<0.000	0.002	-0.003; 0.004	0.843
Age (k=23)	-<0.000	0.002	-0.005; 0.004	0.929
BMI (k=15)	0.010	0.014	-0.017; 0.036	0.482
Outcome assessment				
Single item (ref) (k= 27)				
Standardized questionnaire (k=16)	-0.013	0.056	-0.123; 0.098	0.821

Note: *na: not applicable, SE: standard error, 95% CI: 95% confidence interval; ref: reference category; the reference category was defined as the most numerous category; significant predictors in bold.*

Supplementary Table S13

Metaregression of factor affecting prevalence of Emotional eating

Emotional eating	EST.	S.E.	95% CI of Est.	P value
Risk of bias				
High risk (ref) (k=9)				
Medium risk (k=7)	-0.085	0.006	-0.214; 0.044	0.196
Low risk (k<3)	na	na	na	na
Recruitment time				
First wave (ref) (k=14)				
Second wave (k<3)	na	na	na	na
Country				
Asia (ref) (k= 6)				
Africa (k<3)	na	na	na	na
Europa (k=5)	0.100	0.102	-0.100; 0.301	0.327
North America (k=5)	0.044	0.102	-0.157; 0.244	0.670
Type of sample				
General adult population (ref) (k=14)				
Women (k=<3)	na	na	na	na
Students (k=<3)	na	na	na	na
% Female (k= 15)	-0.002	0.004	-0.010; 0.005	0.494
Age (k=14)	0.009	0.006	-0.002; 0.021	0.114
BMI (k=8)	0.031	0.043	-0.053; 0.116	0.468
Outcome assessment				
Single item (ref) (k=7)				
Standardized questionnaire (k=10)	0.024	0.080	-0.133; 0.180	0.768

Note: *na: not applicable, SE: standard error, 95% CI: 95% confidence interval; ref: reference category; the reference category was defined as the most numerous category; significant predictors in bold.*

Supplementary Figure S1

Forrest and Funnel Plot of weight gain in general population during the pandemic. 95% CI = 95% confidence interval; ES = effect size

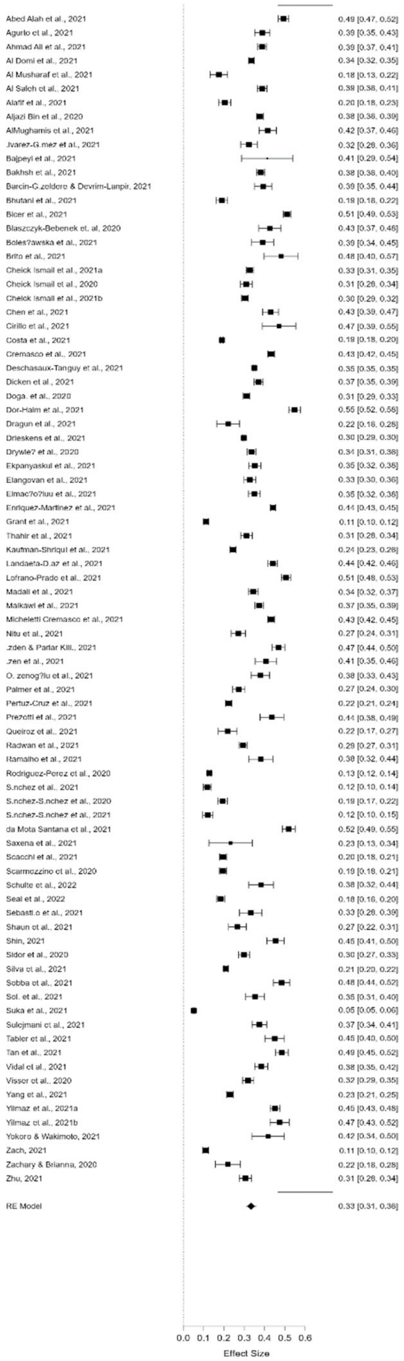

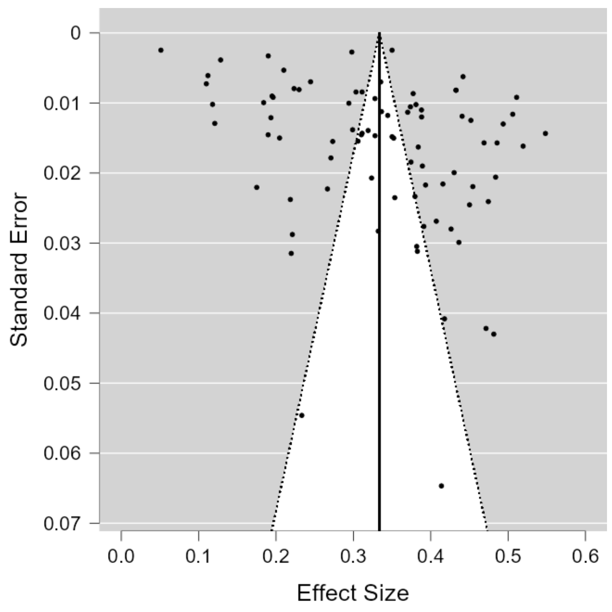

Standard Error
0
0.01
0.02
0.03
0.04
0.05
0.06
0.07
0.0
0.1
0.2
0.3
0.4
0.5
0.6
Effect Size

Supplementary Figure S2

Forrest and Funnel Plot of food restriction in general population during the pandemic. 95% CI = 95% confidence interval; ES = effect size

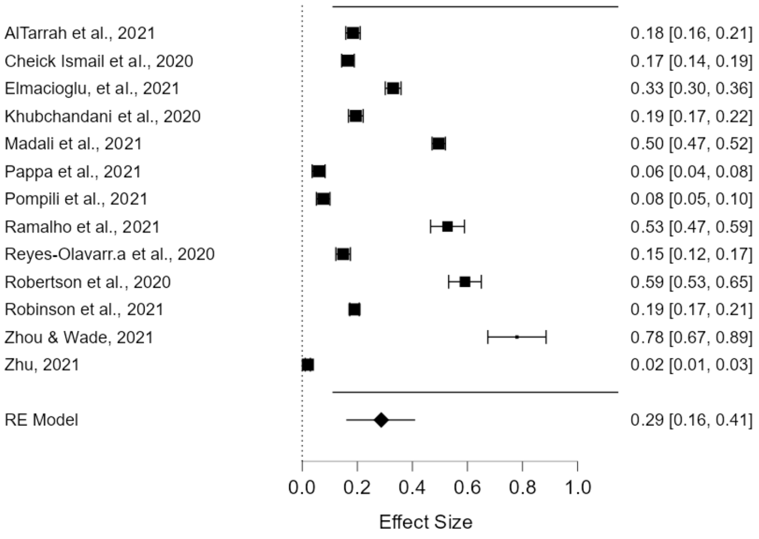

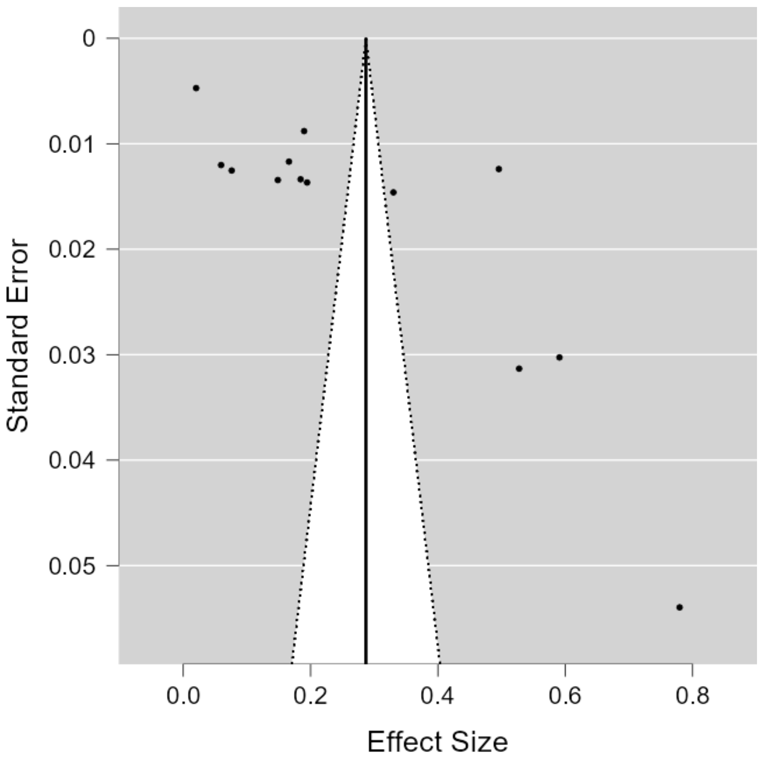

Supplementary Figure S3

Forrest and Funnel Plot of Body shape concerns in general population during the pandemic. 95% CI = 95% confidence interval; ES = effect size

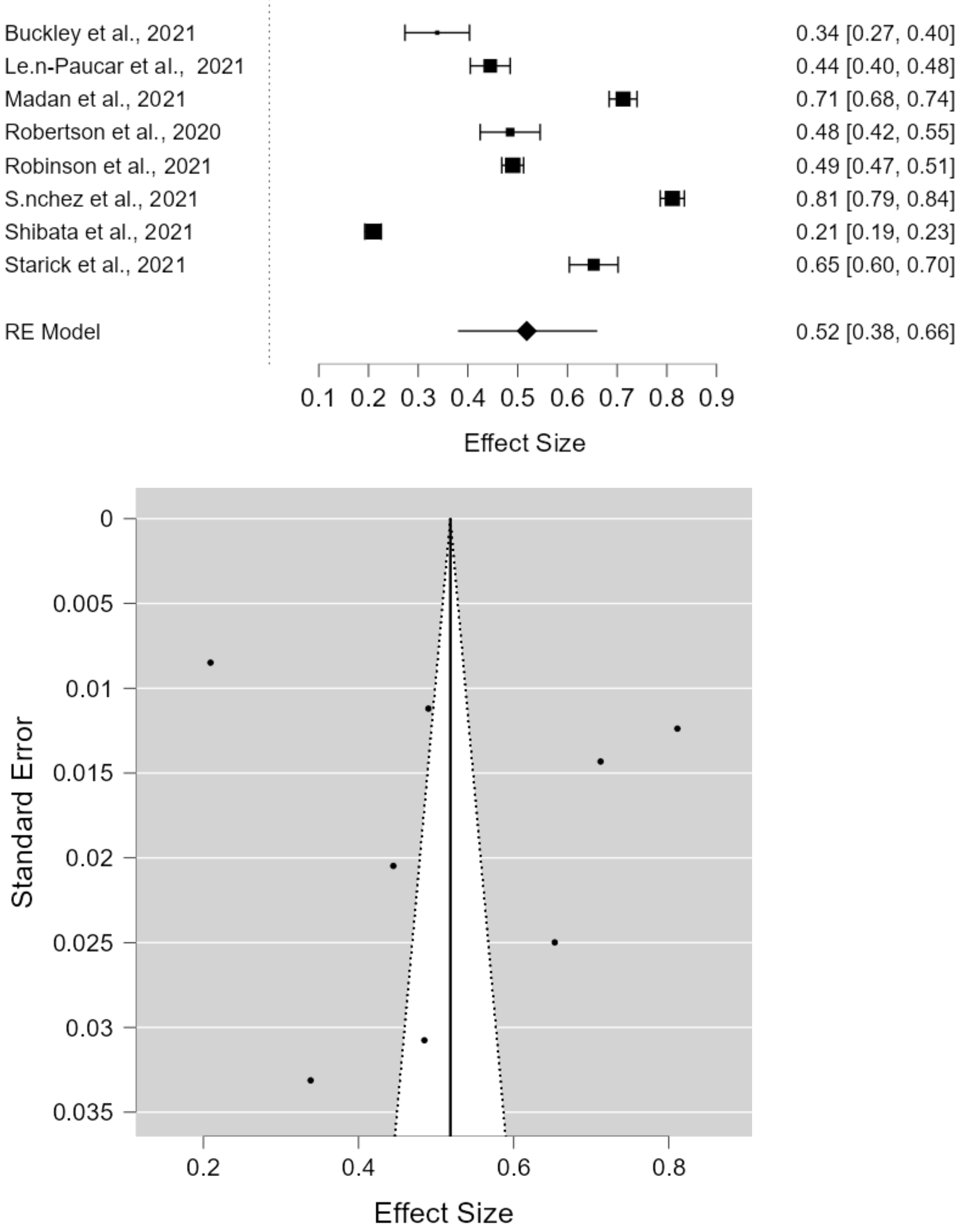

Supplementary Figure S4

Forrest and Funnel Plot of weight loss in general population during the pandemic. 95% CI = 95% confidence interval; ES = effect size

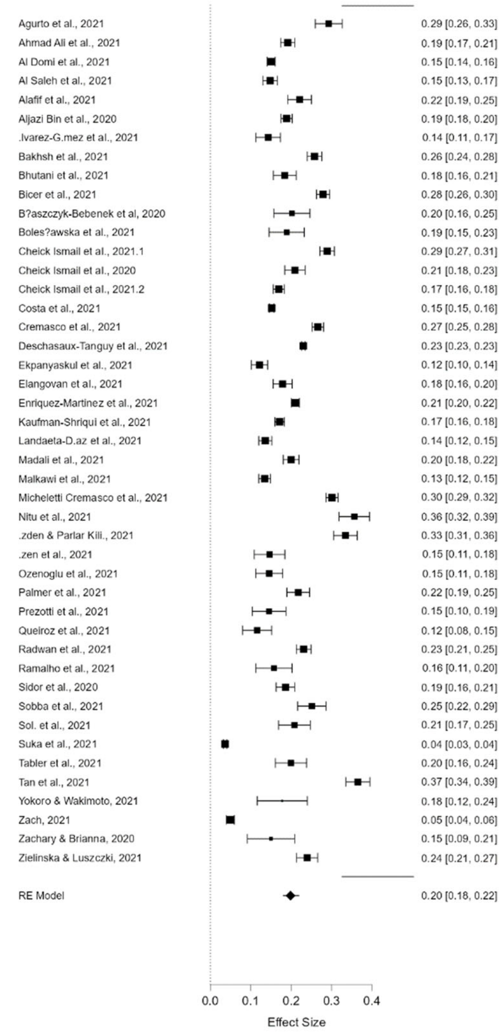

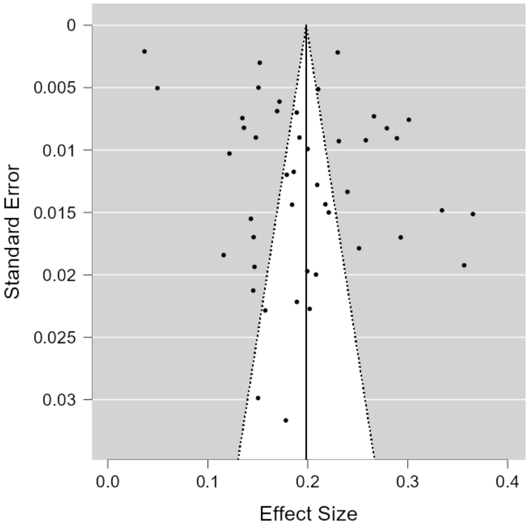

Supplementary Figure S5

Forrest and Funnel Plot of excessive physical activity in general population during the pandemic. 95% CI = 95% confidence interval; ES = effect size

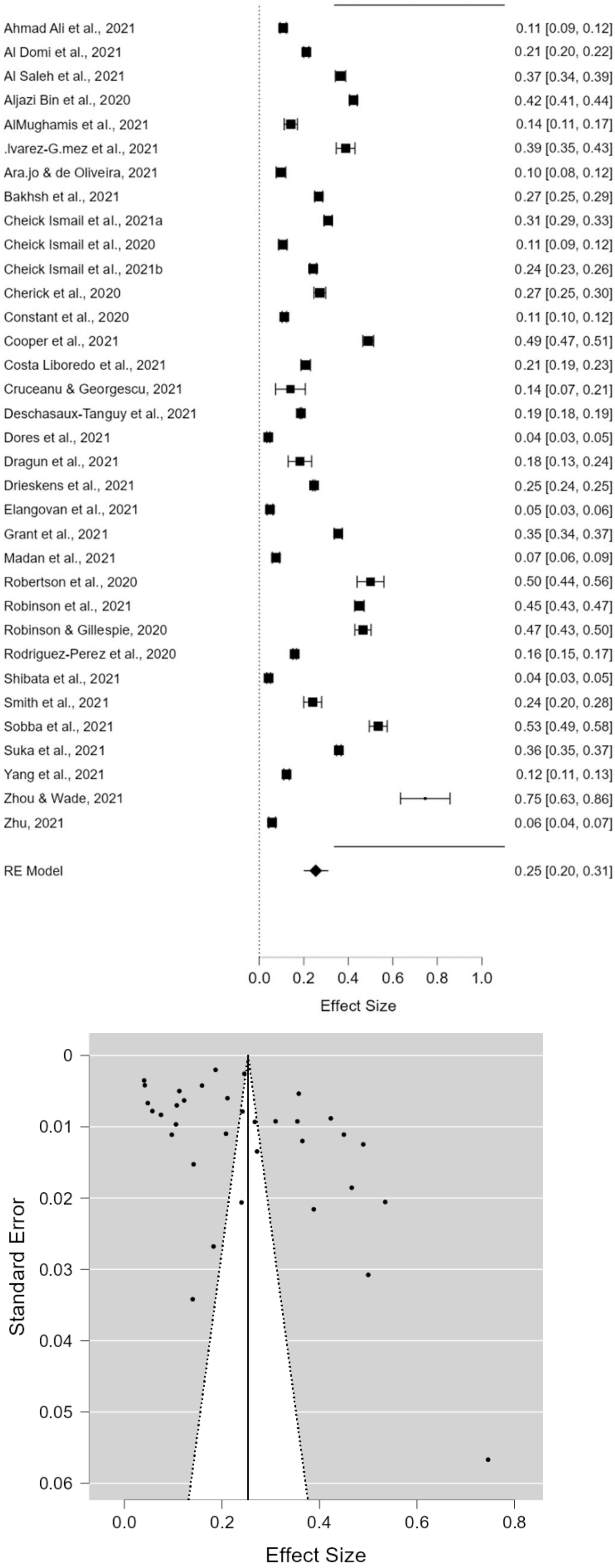

Supplementary Figure S6

Forrest and Funnel Plot of bingeing in general population during the pandemic. 95% CI = 95% confidence interval; ES = effect size

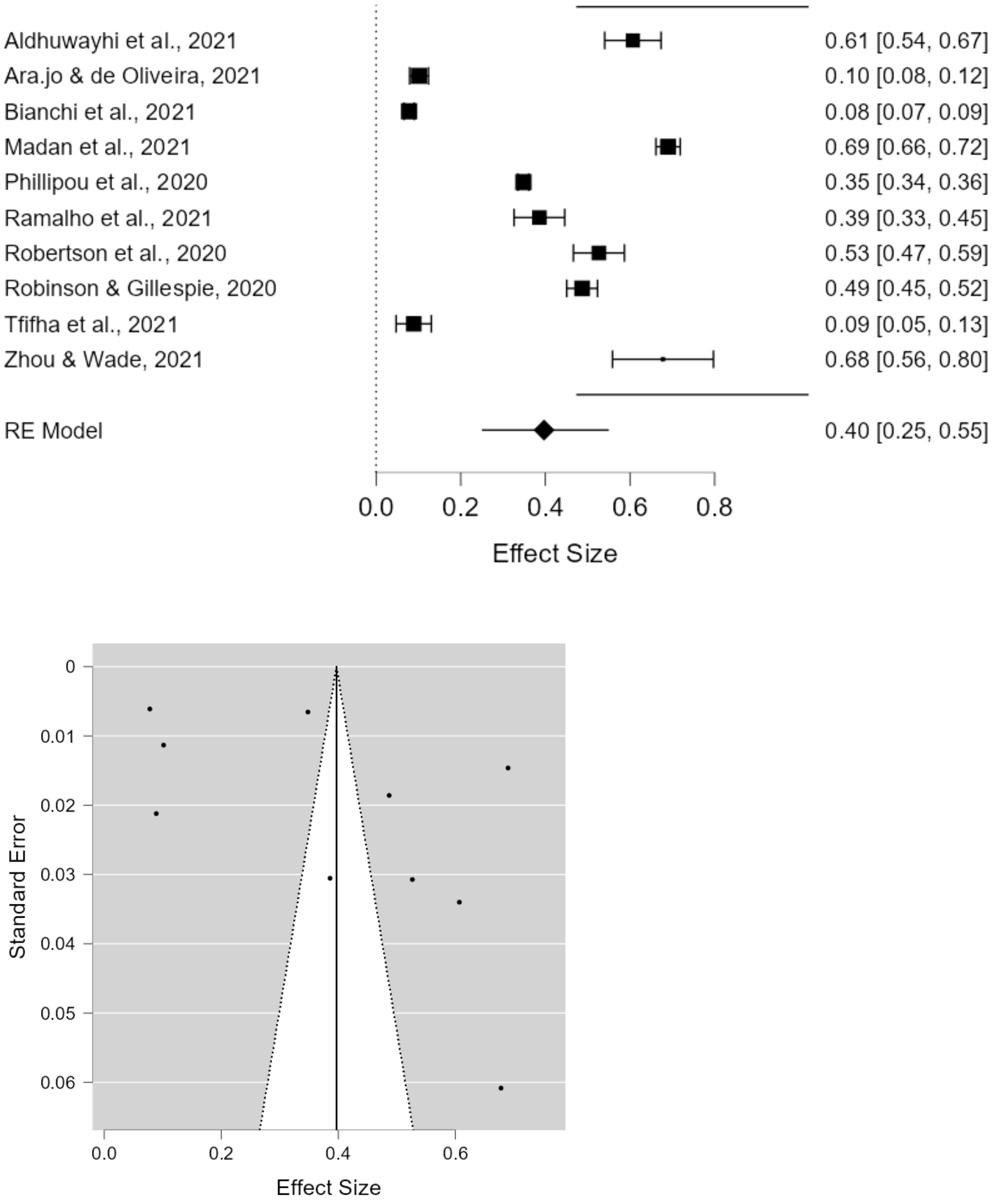

Supplementary Figure S7

Forrest and Funnel Plot of overeating in general population during the pandemic. 95% CI = 95% confidence interval; ES = effect size

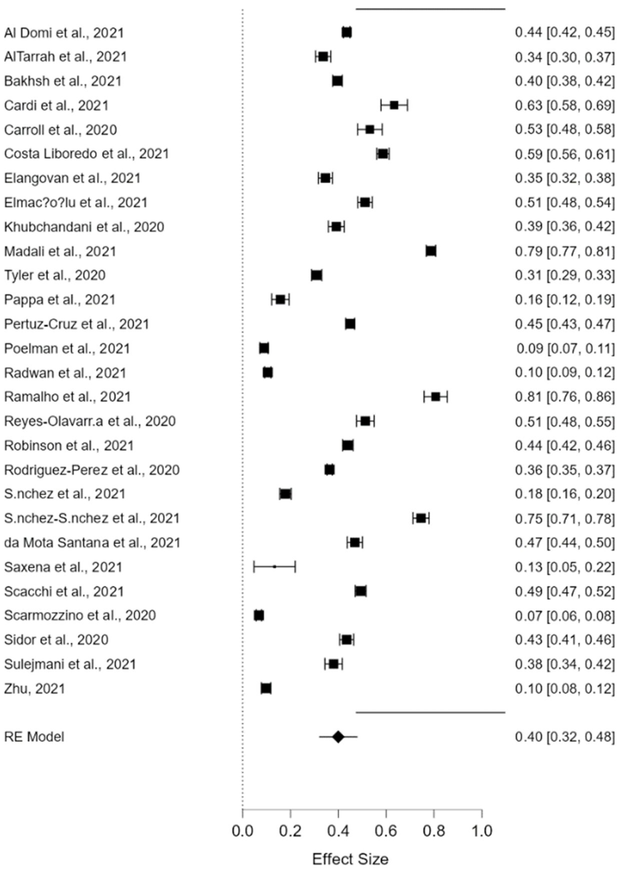

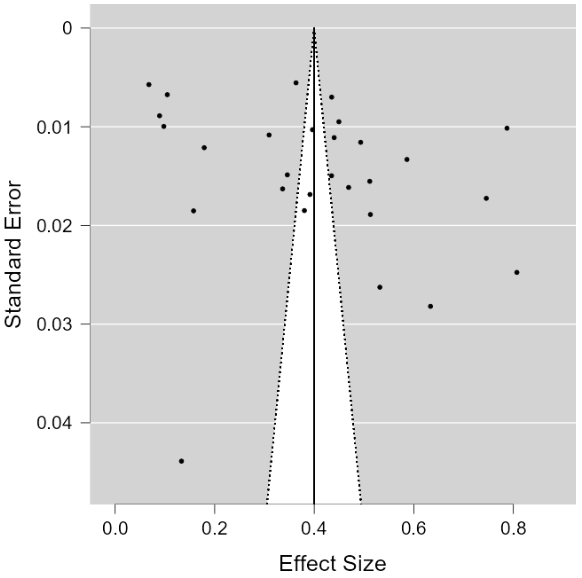

Supplementary Figure S8

Forrest and Funnel Plot of food craving in general population during the pandemic. 95% CI = 95% confidence interval; ES = effect size

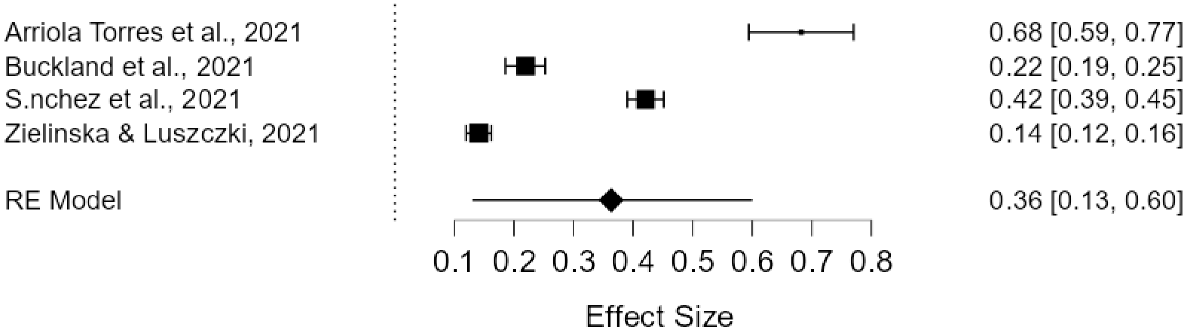

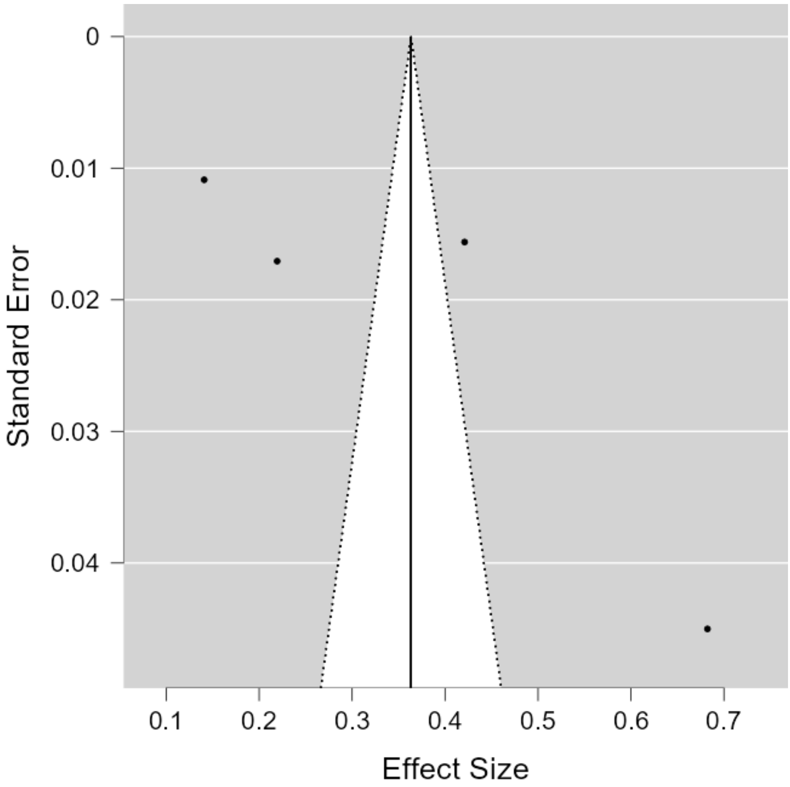

Supplementary Figure S9

Forrest and Funnel Plot of snacking in general population during the pandemic. 95% CI = 95% confidence interval; ES = effect size

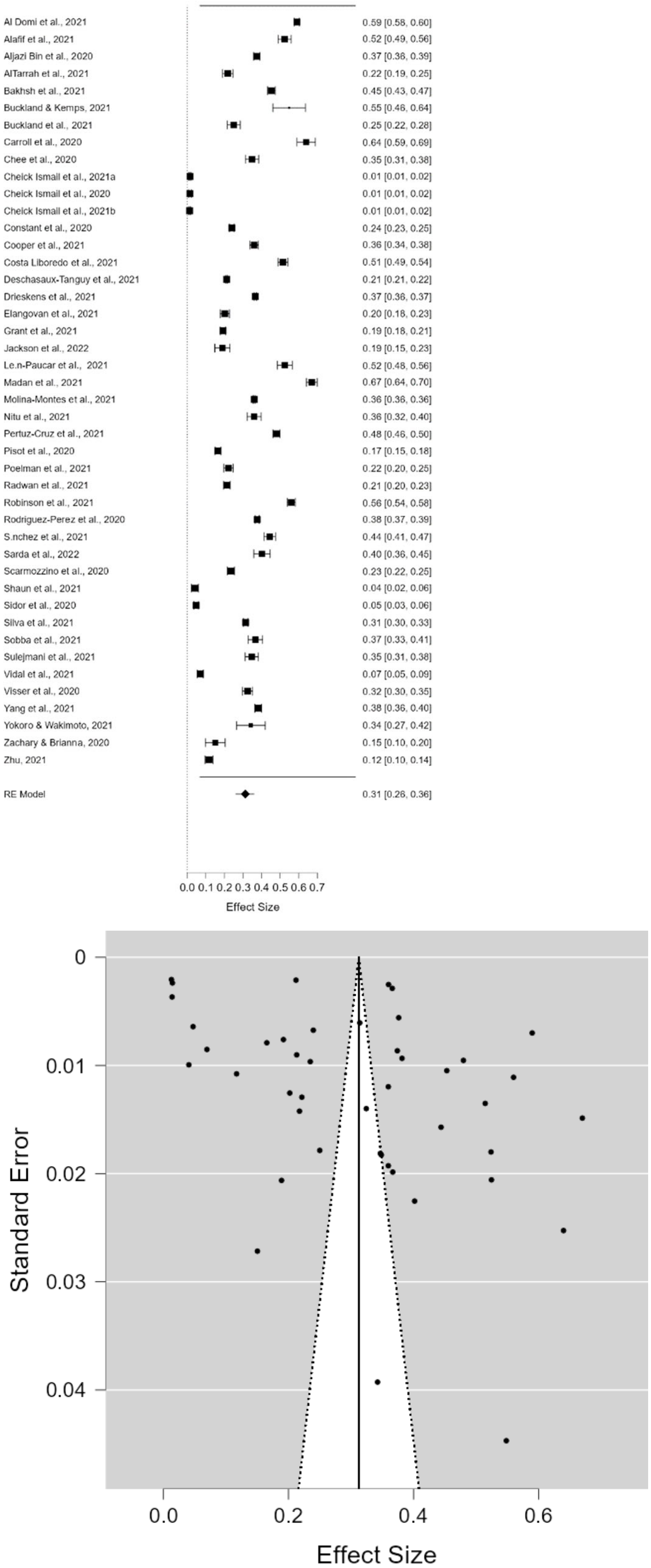

Supplementary Figure S10

Forrest and Funnel Plot of night eating in general population during the pandemic. 95% CI = 95% confidence interval; ES = effect size

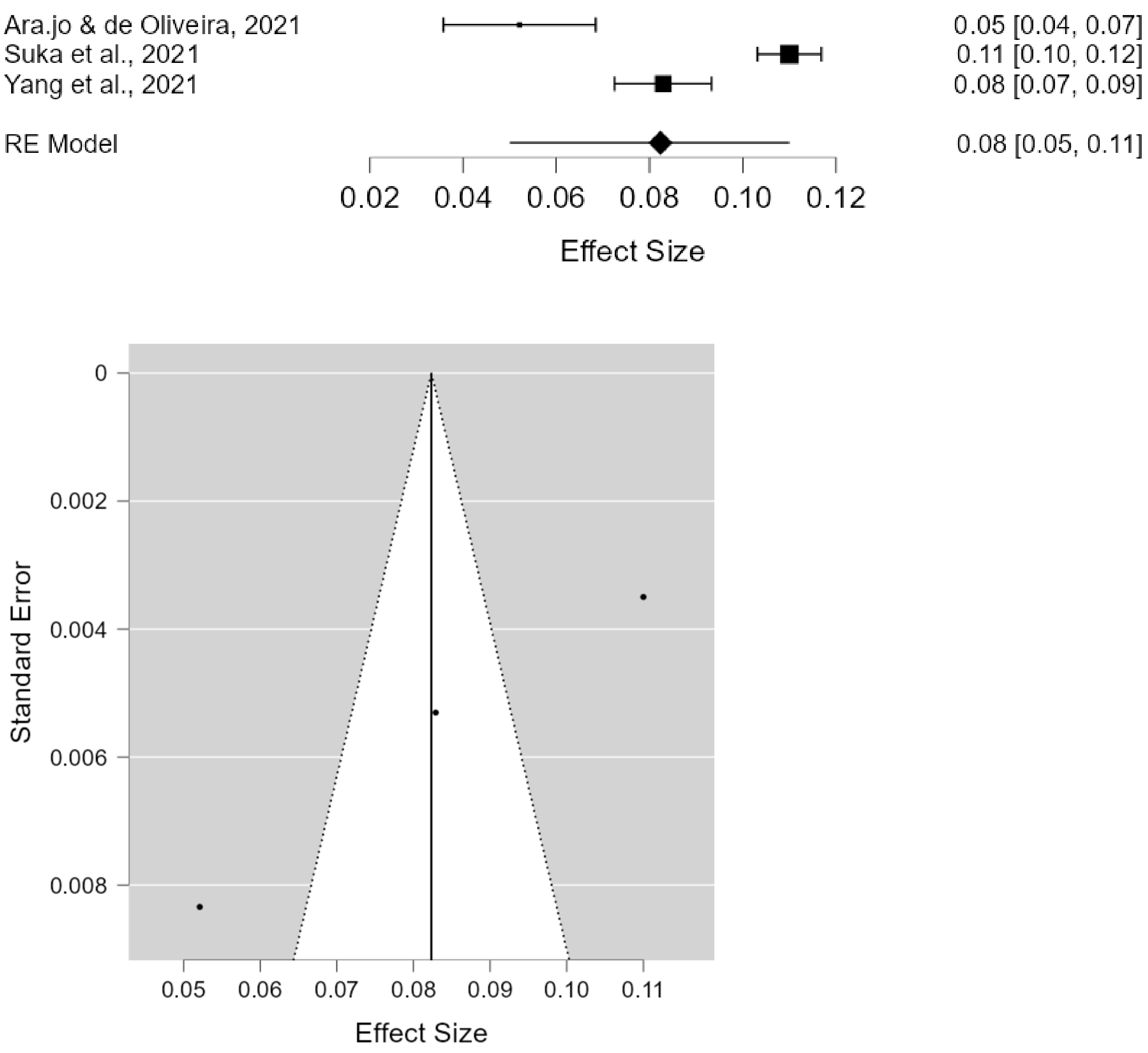

Supplementary Figure S11

Forrest and Funnel Plot of emotional eating in general population during the pandemic. 95% CI = 95% confidence interval; ES = effect size

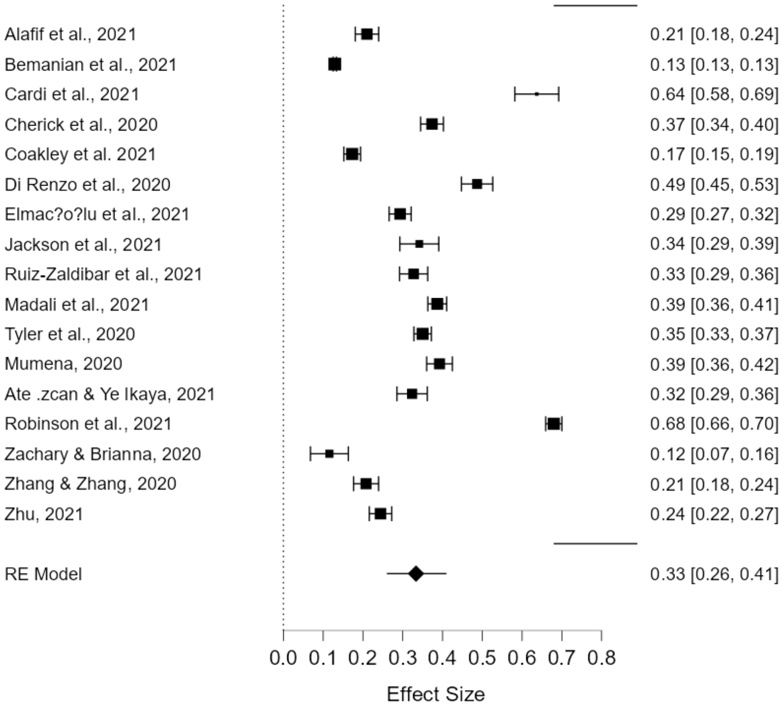

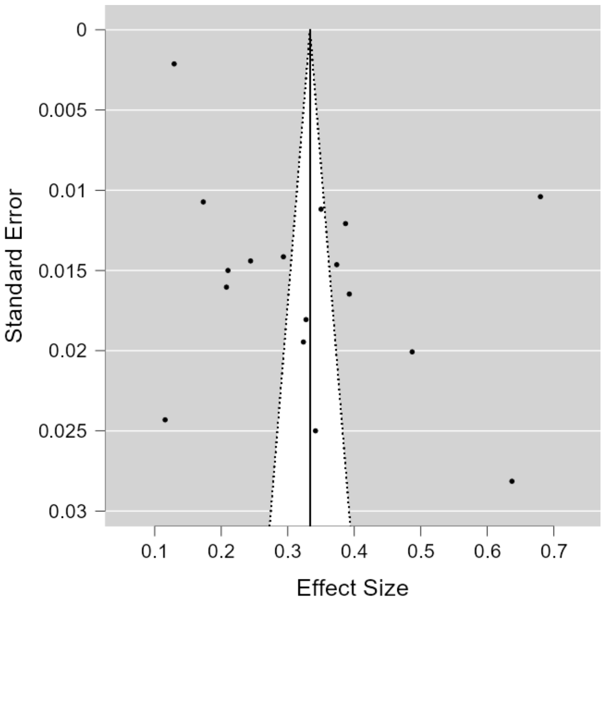

Supplementary Figures S12

Forrest and Funnel Plot of change in weight gain from pre-pandemic to pandemic time.

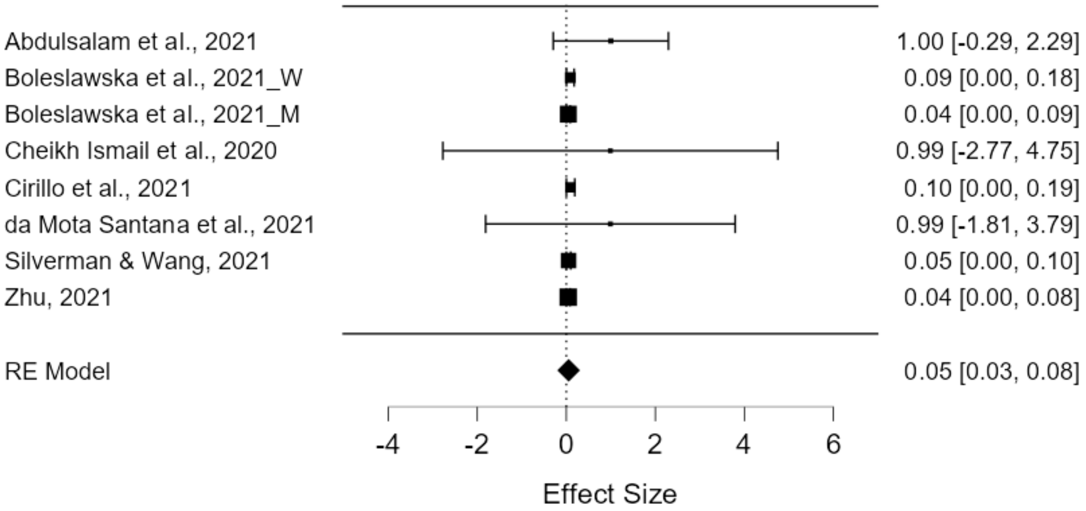

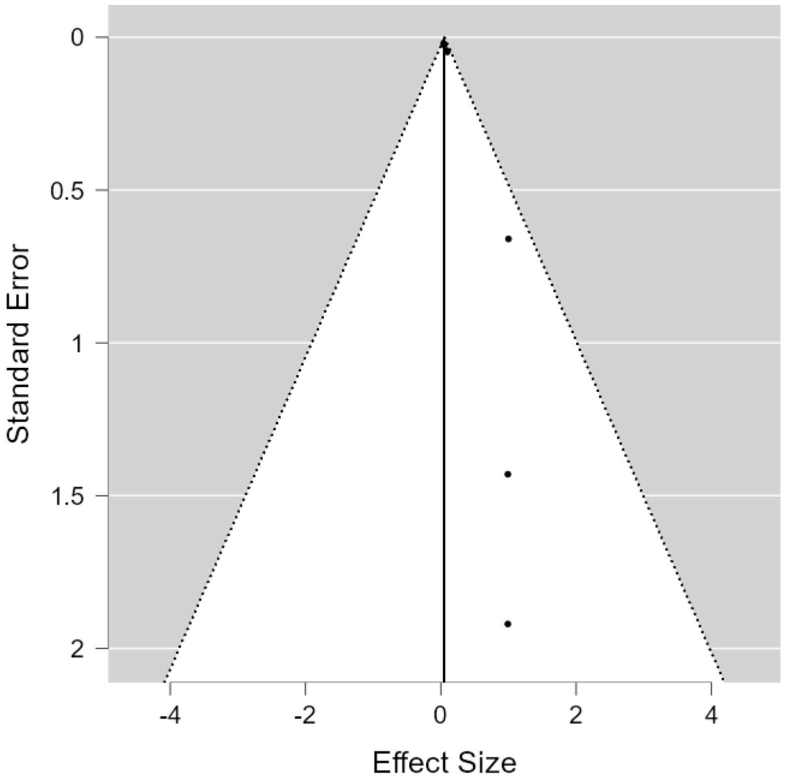

Supplementary Figure S13

Forrest and Funnel Plot of change in weight gain during the pandemic.

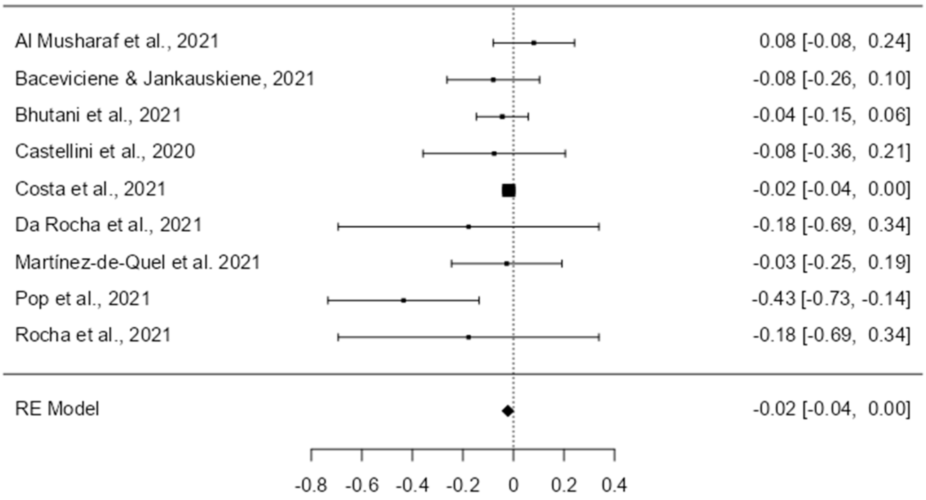

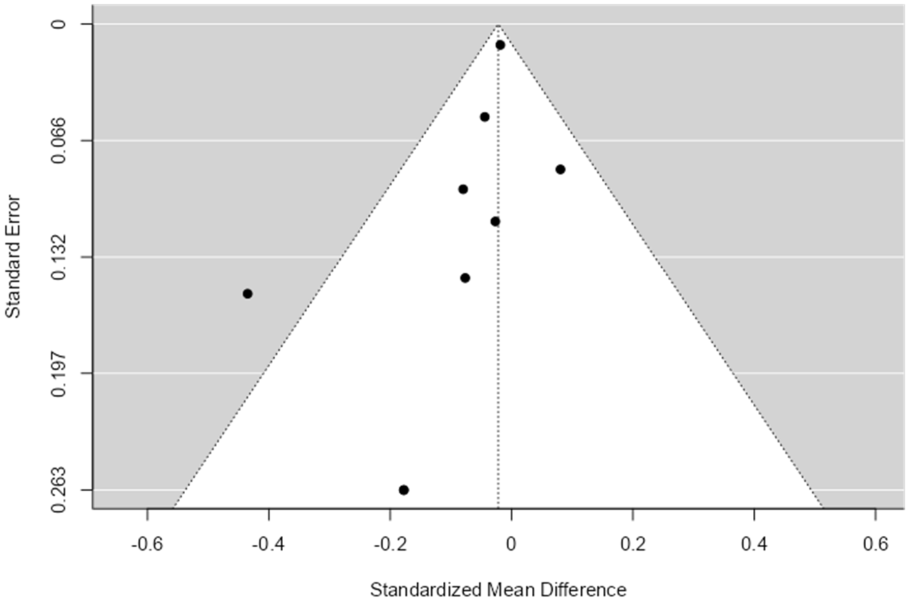

Supplementary Figure S14

Forrest and Funnel Plot of change in food restriction from pre-pandemic to pandemic time.

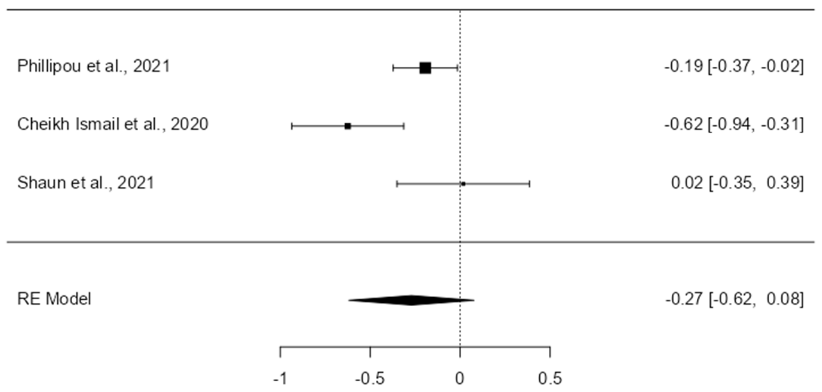

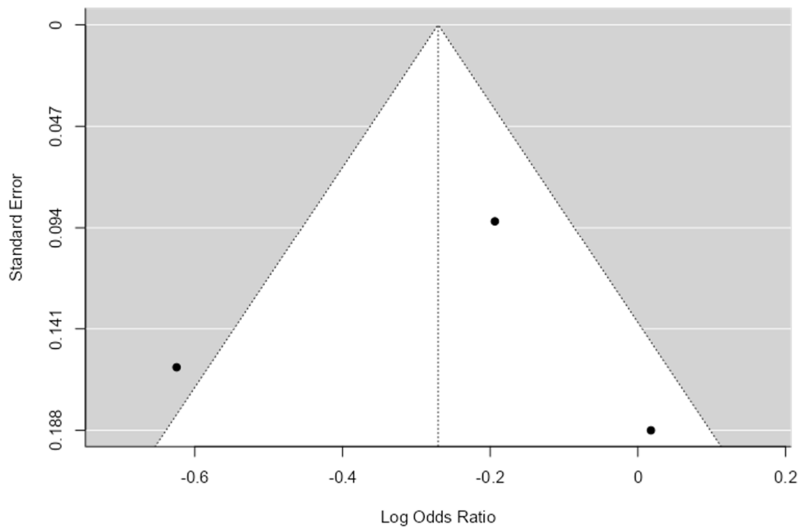

Supplementary Figure S15

Forrest and Funnel Plot of change in excessive physical exercise from pre-pandemic to pandemic time.

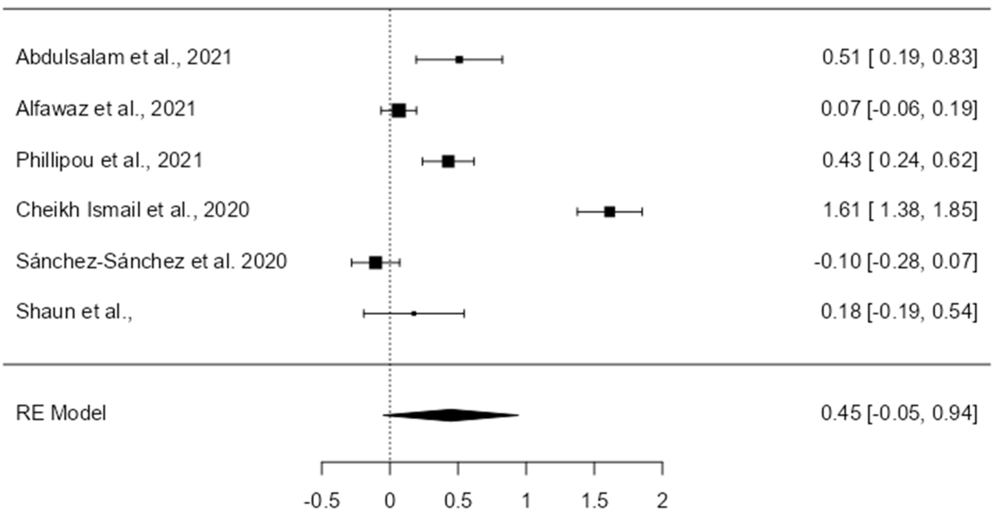

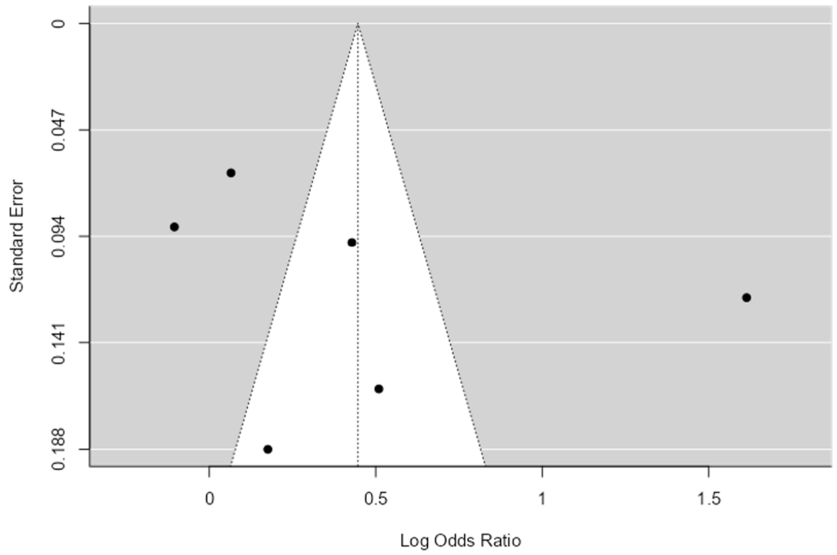

Supplementary Figure S16

Forrest and Funnel Plot of change in excessive binge eating from pre-pandemic to pandemic time.

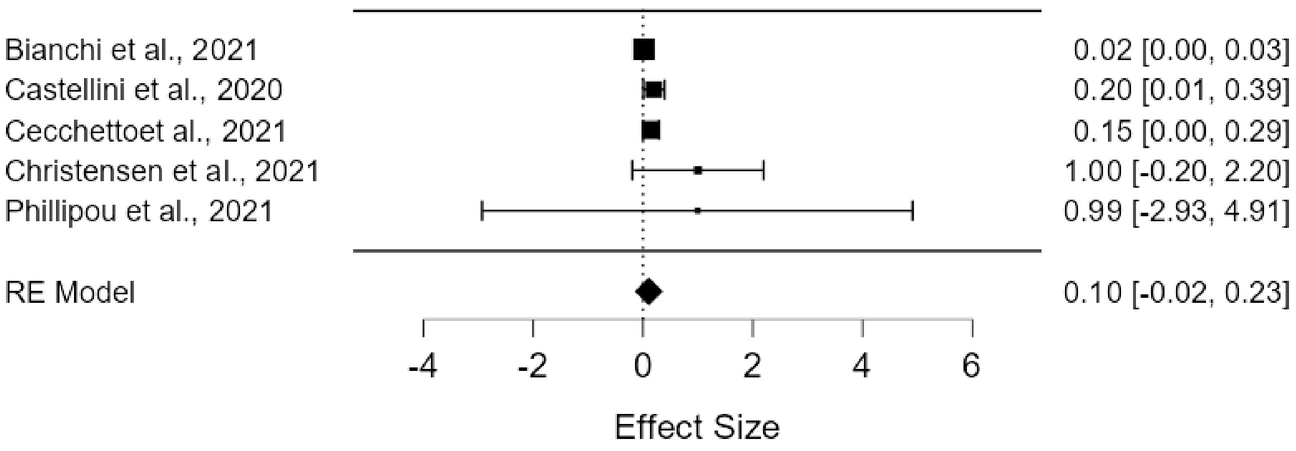

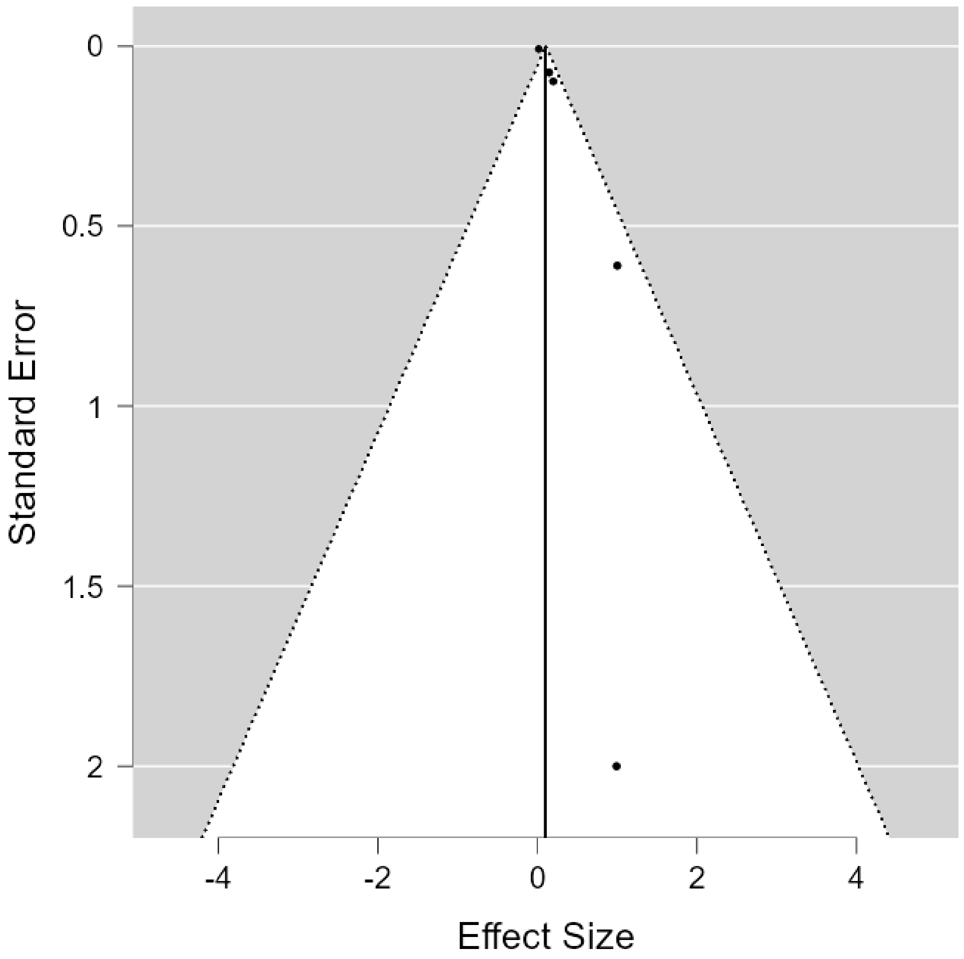

References

1. Abdulsalam, N. M.; Khateeb, N. A.; Aljerbi, S. S.; Alqumayzi, W. M.; Balubaid, S. S.; Almarghlani, A. A.; Ayad, A. A.; Williams, L. L. Assessment of Dietary Habits and Physical Activity Changes during the Full COVID-19 Curfew Period and Its Effect on Weight among Adults in Jeddah, Saudi Arabia. *Int J Environ Res Public Health* **2021**, *18* (16), 8580. https://doi.org/10.3390/ijerph18168580.
2. Abed Alah, M.; Abdeen, S.; Kehyayan, V.; Bougmiza, I. Impact of Staying at Home Measures during COVID-19 Pandemic on the Lifestyle of Qatar's Population: Perceived Changes in Diet, Physical Activity, and Body Weight. *Prev Med Rep* **2021**, *24*, 101545. https://doi.org/10.1016/j.pmedr.2021.101545.
3. Agurto, H. S.; Alcantara-Diaz, A. L.; Espinet-Coll, E.; Toro-Huamanchumo, C. J. Eating Habits, Lifestyle Behaviors and Stress during the COVID-19 Pandemic Quarantine among Peruvian Adults. *PeerJ* **2021**, *9*, e11431. https://doi.org/10.7717/peerj.11431.
4. Al-Domi, H.; AL-Dalaeen, A.; AL-Rosan, S.; Batarseh, N.; Nawaiseh, H. Healthy Nutritional Behavior during COVID-19 Lockdown: A Cross-Sectional Study. *Clin Nutr ESPEN* **2021**, *42*, 132–137. https://doi.org/10.1016/j.clnesp.2021.02.003.
5. Al-Musharaf, S. Prevalence and Predictors of Emotional Eating among Healthy Young Saudi Women during the COVID-19 Pandemic. *Nutrients* **2020**, *12* (10), 2923. https://doi.org/10.3390/nu12102923.
6. Al-Musharaf, S.; Aljuraiban, G.; Bogis, R.; Alnafisah, R.; Aldhwayan, M.; Tahrani, A. Lifestyle Changes Associated with COVID-19 Quarantine among Young Saudi Women: A Prospective Study. *PLoS One* **2021**, *16* (4), e0250625. https://doi.org/10.1371/journal.pone.0250625.
7. Al-Saleh, M.; Alamri, A.; Alhefzi, A.; Assiri, K.; Moshebah, A. Population Healthy Lifestyle Changes in Abha City during COVID-19 Lockdown, Saudi Arabia. *J Family Med Prim Care* **2021**, *10* (2), 809. https://doi.org/10.4103/jfmpc.jfmpc_1224_20.
8. Alafif, N. O.; Abdelfattah, E. H.; Al hadi, R. A.; Alanazi, S. B.; Alkabaa, R. I.; Alsalem, F. A.; Aljeldah, T. M.; Aldriweesh, K. K.; Albati, A. A. Effect of Quarantine on Eating Behaviors and Weight Change among King Saud University Students in Riyadh. *J King Saud Univ Sci* **2021**, *33* (8), 101609. https://doi.org/10.1016/j.jksus.2021.101609.
9. Aldhuwayhi, S.; Shaikh, S. A.; Mallineni, S. K.; Kumari, V. V.; Thakare, A. A.; Ahmed Khan, A. R.; Mustafa, M. Z.; Manva, M. Z. Occupational Stress and Stress Busters Used Among Saudi Dental Practitioners During the COVID-19 Pandemic Outbreak. *Disaster Med Public Health Prep* **2022**, *16* (5), 1975–1981. https://doi.org/10.1017/dmp.2021.215.
10. Alfawaz, H.; Amer, O. E.; Aljumah, A. A.; Aldisi, D. A.; Enani, M. A.; Aljohani, N. J.; Alotaibi, N. H.; Alshingetti, N.; Alomar, S. Y.; Khattak, M. N. K.; Sabico, S.; Al-Daghri, N. M. Effects of Home Quarantine during COVID-19 Lockdown on Physical Activity and Dietary Habits of Adults in Saudi Arabia. *Sci Rep* **2021**, *11* (1), 5904. https://doi.org/10.1038/s41598-021-85330-2.
11. Ali, A.; Sohaib, M.; Iqbal, S.; Hayat, K.; Khan, A. U.; Rasool, M. F. Evaluation of COVID-19 Disease Awareness and Its Relation to Mental Health, Dietary Habits, and Physical Activity: A Cross-Sectional Study from Pakistan. *Am J Trop Med Hyg* **2021**, *104* (5), 1687–1693. https://doi.org/10.4269/ajtmh.20-1451.
12. AlMughamis, N.; AlAsfour, S.; Mehmood, S. Poor Eating Habits and Predictors of Weight Gain during the COVID-19 Quarantine Measures in Kuwait: A Cross Sectional Study. *F1000Res* **2020**, *9*, 914. https://doi.org/10.12688/f1000research.25303.1.
13. AlTarrah, D.; AlShami, E.; AlHamad, N.; AlBesher, F.; Devarajan, S. The Impact of Coronavirus COVID-19 Pandemic on Food Purchasing, Eating Behavior, and Perception of Food Safety in Kuwait. *Sustainability* **2021**, *13* (16), 8987. https://doi.org/10.3390/su13168987.

14. Álvarez-Gómez, C.; De La Higuera, M.; Rivas-García, L.; Diaz-Castro, J.; Moreno-Fernandez, J.; Lopez-Frias, M. Has COVID-19 Changed the Lifestyle and Dietary Habits in the Spanish Population after Confinement? *Foods* **2021**, *10* (10), 2443. https://doi.org/10.3390/foods10102443.
15. Ammar, A.; Brach, M.; Trabelsi, K.; Chtourou, H.; Boukhris, O.; Masmoudi, L.; Bouaziz, B.; Bentlage, E.; How, D.; Ahmed, M.; Müller, P.; Müller, N.; Aloui, A.; Hammouda, O.; Paineiras-Domingos, L.; Braakman-Jansen, A.; Wrede, C.; Bastoni, S.; Pernambuco, C.; Mataruna, L.; Taheri, M.; Irandoust, K.; Khacharem, A.; Bragazzi, N.; Chamari, K.; Glenn, J.; Bott, N.; Gargouri, F.; Chaari, L.; Batatia, H.; Ali, G.; Abdelkarim, O.; Jarraya, M.; El Abed, K.; Souissi, N.; Van Gemert-Pijnen, L.; Riemann, B.; Riemann, L.; Moalla, W.; Gómez-Raja, J.; Epstein, M.; Sanderman, R.; Schulz, S.; Jerg, A.; Al-Horani, R.; Mansi, T.; Jmail, M.; Barbosa, F.; Ferreira-Santos, F.; Šimunič, B.; Pišot, R.; Gaggioli, A.; Bailey, S.; Steinacker, J.; Driss, T.; Hoekelmann, A. Effects of COVID-19 Home Confinement on Eating Behaviour and Physical Activity: Results of the ECLB-COVID19 International Online Survey. *Nutrients* **2020**, *12* (6), 1583. https://doi.org/10.3390/nu12061583.
16. Arriola Torres, L. F.; Palomino Taype, K. R.; Quintana Castro, L. Calidad de Sueño y Antojo Por Azúcares En Médicos Residentes Durante La Pandemia de COVID-19 En El Perú. *Neurología Argentina* **2021**, *13* (1), 7–13. https://doi.org/10.1016/j.neuarg.2021.01.004.
17. Baceviciene, M.; Jankauskiene, R. Changes in Sociocultural Attitudes towards Appearance, Body Image, Eating Attitudes and Behaviours, Physical Activity, and Quality of Life in Students before and during COVID-19 Lockdown. *Appetite* **2021**, *166*, 105452. https://doi.org/10.1016/j.appet.2021.105452.
18. Bajpeyi, S.; Jung, H.; Carrillo, I. A.; Nunez, A. V.; Umucu, E. Role Of Mental Health On Weight Gain During Covid-19 Pandemic Among Older Adults In Subsidized Housing. *University of Texas at El Paso*. 2021.
19. Bakhsh, M. A.; Khawandanah, J.; Naaman, R. K.; Alashmali, S. The Impact of COVID-19 Quarantine on Dietary Habits and Physical Activity in Saudi Arabia: A Cross-Sectional Study. *BMC Public Health* **2021**, *21* (1), 1487. https://doi.org/10.1186/s12889-021-11540-y.
20. Barcln-Güzeldere, H. K.; Devrim-Lanpir, A. The Association between Body Mass Index, Emotional Eating and Perceived Stress during COVID-19 Partial Quarantine in Healthy Adults. *Public Health Nutr* **2022**, *25* (1), 43–50. https://doi.org/10.1017/S1368980021002974.
21. Bemanian, M.; Mæland, S.; Blomhoff, R.; Rabben, Å. K.; Arnesen, E. K.; Skogen, J. C.; Fadnes, L. T. Emotional Eating in Relation to Worries and Psychological Distress Amid the COVID-19 Pandemic: A Population-Based Survey on Adults in Norway. *Int J Environ Res Public Health* **2020**, *18* (1), 130. https://doi.org/10.3390/ijerph18010130.
22. Bhutani, S.; vanDellen, M. R.; Haskins, L. B.; Cooper, J. A. Energy Balance-Related Behavior Risk Pattern and Its Correlates During COVID-19 Related Home Confinement. *Front Nutr* **2021**, *8*. https://doi.org/10.3389/fnut.2021.680105.
23. Bhutani, S.; Vandellen, M. R.; Cooper, J. A. Longitudinal Weight Gain and Related Risk Behaviors during the Covid-19 Pandemic in Adults in the Us. *Nutrients* **2021**, *13* (2), 1–14. https://doi.org/10.3390/nu13020671.
24. Bianchi, D.; Baiocco, R.; Pompili, S.; Lonigro, A.; Di Norcia, A.; Cannoni, E.; Longobardi, E.; Zammuto, M.; Di Tata, D.; Laghi, F. Binge Eating and Binge Drinking in Emerging Adults During COVID-19 Lockdown in Italy: An Examination of Protective and Risk Factors. *Emerging Adulthood* **2022**, *10* (1), 291–303. https://doi.org/10.1177/21676968211058501.
25. Biçer, N. Ç.; Baş, M.; Köse, G.; Duru, P. Ş.; Baş, D.; Karaca, E.; Köseoğlu, S. Lockdown Changed Us in Turkey Eating Behaviors, Depression Levels, and Body Weight Changes during Lockdown. *Progress in Nutrition* **2021**, *23* (3), 11–11. https://doi.org/10.23751/pn.v23i3.11856.
26. Bin Zarah, A.; Enriquez-Marulanda, J.; Andrade, J. M. Relationship between Dietary Habits, Food Attitudes and Food Security Status among Adults Living within the United States Three Months

Post-Mandated Quarantine: A Cross-Sectional Study. *Nutrients* **2020**, *12* (11), 3468. https://doi.org/10.3390/nu12113468.
27. Błaszczyk-Bębenek, E.; Jagielski, P.; Bolesławska, I.; Jagielska, A.; Nitsch-Osuch, A.; Kawalec, P. Nutrition Behaviors in Polish Adults before and during COVID-19 Lockdown. *Nutrients* **2020**, *12* (10), 3084. https://doi.org/10.3390/nu12103084.
28. Bolesławska, I.; Błaszczyk-Bębenek, E.; Jagielski, P.; Jagielska, A.; Przysławski, J. Nutritional Behaviors of Women and Men in Poland during Confinement Related to the SARS-CoV-2 Epidemic. *Sci Rep* **2021**, *11* (1), 19984. https://doi.org/10.1038/s41598-021-99561-w.
29. Boukrim, M.; Obtel, M.; Kasouati, J.; Achbani, A.; Razine, R. Covid-19 and Confinement: Effect on Weight Load, Physical Activity and Eating Behavior of Higher Education Students in Southern Morocco. *Ann Glob Health* **2021**, *87* (1), 7. https://doi.org/10.5334/aogh.3144.
30. Breiner, C. E.; Miller, M. L.; Hormes, J. M. Changes in Eating and Exercise Behaviors during the COVID-19 Pandemic in a Community Sample: A Retrospective Report. *Eat Behav* **2021**, *42*, 101539. https://doi.org/10.1016/j.eatbeh.2021.101539.
31. Brito, L. M. S.; Lima, V. A. de; Mascarenhas, L. P.; Mota, J.; Leite, N. PHYSICAL ACTIVITY, EATING HABITS AND SLEEP DURING SOCIAL ISOLATION: FROM YOUNG ADULT TO ELDERLY. *Revista Brasileira de Medicina do Esporte* **2021**, *27* (1), 21–25. https://doi.org/10.1590/1517-8692202127012020_0061.
32. Buckland, N. J.; Kemps, E. Low Craving Control Predicts Increased High Energy Density Food Intake during the COVID-19 Lockdown: Result Replicated in an Australian Sample. *Appetite* **2021**, *166*, 105317. https://doi.org/10.1016/j.appet.2021.105317.
33. Buckland, N. J.; Swinnerton, L. F.; Ng, K.; Price, M.; Wilkinson, L. L.; Myers, A.; Dalton, M. Susceptibility to Increased High Energy Dense Sweet and Savoury Food Intake in Response to the COVID-19 Lockdown: The Role of Craving Control and Acceptance Coping Strategies. *Appetite* **2021**, *158*, 105017. https://doi.org/10.1016/j.appet.2020.105017.
34. Buckley, G. L.; Hall, L. E.; Lassemillante, A.-C. M.; Belski, R. Disordered Eating & Body Image of Current and Former Athletes in a Pandemic; a Convergent Mixed Methods Study - What Can We Learn from COVID-19 to Support Athletes through Transitions? *J Eat Disord* **2021**, *9* (1), 73. https://doi.org/10.1186/s40337-021-00427-3.
35. Cardi, V.; Albano, G.; Gentili, C.; Sudulich, L. The Impact of Emotion Regulation and Mental Health Difficulties on Health Behaviours during COVID19. *J Psychiatr Res* **2021**, *143*, 409–415. https://doi.org/10.1016/j.jpsychires.2021.10.001.
36. Carroll, N.; Sadowski, A.; Laila, A.; Hruska, V.; Nixon, M.; Ma, D.; Haines, J. The Impact of COVID-19 on Health Behavior, Stress, Financial and Food Security among Middle to High Income Canadian Families with Young Children. *Nutrients* **2020**, *12* (8), 2352. https://doi.org/10.3390/nu12082352.
37. Caso, D.; Guidetti, M.; Capasso, M.; Cavazza, N. Finally, the Chance to Eat Healthily: Longitudinal Study about Food Consumption during and after the First COVID-19 Lockdown in Italy. *Food Qual Prefer* **2022**, *95*, 104275. https://doi.org/10.1016/j.foodqual.2021.104275.
38. Castellini, G.; Cassioli, E.; Rossi, E.; Innocenti, M.; Gironi, V.; Sanfilippo, G.; Felciai, F.; Monteleone, A. M.; Ricca, V. The Impact of <scp>COVID</Scp> -19 Epidemic on Eating Disorders: A Longitudinal Observation of Pre versus Post Psychopathological Features in a Sample of Patients with Eating Disorders and a Group of Healthy Controls. *International Journal of Eating Disorders* **2020**, *53* (11), 1855–1862. https://doi.org/10.1002/eat.23368.
39. Cecchetto, C.; Aiello, M.; Gentili, C.; Ionta, S.; Osimo, S. A. Increased Emotional Eating during COVID-19 Associated with Lockdown, Psychological and Social Distress. *Appetite* **2021**, *160*, 105122. https://doi.org/10.1016/j.appet.2021.105122.
40. Chan, C. Y.; Chiu, C. Y. Disordered Eating Behaviors and Psychological Health during the COVID-19 Pandemic. *Psychol Health Med* **2022**, *27* (1), 249–256. https://doi.org/10.1080/13548506.2021.1883687.

41. Chee, M. J.; Koziel Ly, N. K.; Anisman, H.; Matheson, K. Piece of Cake: Coping with COVID-19. *Nutrients* **2020**, *12* (12), 3803. https://doi.org/10.3390/nu12123803.
42. Cheikh Ismail, L.; Osaili, T. M.; Mohamad, M. N.; Al Marzouqi, A.; Jarrar, A. H.; Abu Jamous, D. O.; Magriplis, E.; Ali, H. I.; Al Sabbah, H.; Hasan, H.; AlMarzooqi, L. M. R.; Stojanovska, L.; Hashim, M.; Shaker Obaid, R. R.; Saleh, S. T.; Al Dhaheri, A. S. Eating Habits and Lifestyle during COVID-19 Lockdown in the United Arab Emirates: A Cross-Sectional Study. *Nutrients* **2020**, *12* (11), 3314. https://doi.org/10.3390/nu12113314.
43. Cheikh Ismail, L.; Hashim, M.; Mohamad, M. N.; Hassan, H.; Ajab, A.; Stojanovska, L.; Jarrar, A. H.; Hasan, H.; Abu Jamous, D. O.; Saleh, S. T.; Al Daour, R.; Osaili, T. M.; Al Dhaheri, A. S. Dietary Habits and Lifestyle During Coronavirus Pandemic Lockdown: Experience From Lebanon. *Front Nutr* **2021**, *8*. https://doi.org/10.3389/fnut.2021.730425.
44. Cheikh Ismail, L.; Osaili, T. M.; Mohamad, M. N.; Al Marzouqi, A.; Jarrar, A. H.; Zampelas, A.; Habib-Mourad, C.; Omar Abu Jamous, Di.; Ali, H. I.; Al Sabbah, H.; Hasan, H.; Almarzooqi, L. M. R.; Stojanovska, L.; Hashim, M.; Shaker Obaid, R. R.; Elfeky, S.; Saleh, S. T.; Shawar, Z. A. M.; Al Dhaheri, A. S. Assessment of Eating Habits and Lifestyle during the Coronavirus 2019 Pandemic in the Middle East and North Africa Region: A Cross-Sectional Study. *British Journal of Nutrition* **2021**, *126* (5), 757–766. https://doi.org/10.1017/S0007114520004547.
45. Chen, W. L.; Song, S. Y.; Yap, K. H. The Unintended Consequences of the Pandemic: The New Normal for College Students in South Korea and Taiwan. *Front Public Health* **2021**, *9*. https://doi.org/10.3389/fpubh.2021.598302.
46. Cherikh, F.; Frey, S.; Bel, C.; Attanasi, G.; Alifano, M.; Iannelli, A. Behavioral Food Addiction During Lockdown: Time for Awareness, Time to Prepare the Aftermath. *Obes Surg* **2020**, *30* (9), 3585–3587. https://doi.org/10.1007/s11695-020-04649-3.
47. Christensen, K. A.; Forbush, K. T.; Richson, B. N.; Thomeczek, M. L.; Perko, V. L.; Bjorlie, K.; Christian, K.; Ayres, J.; Wildes, J. E.; Mildrum Chana, S. Food Insecurity Associated with Elevated Eating Disorder Symptoms, Impairment, and Eating Disorder Diagnoses in an American University Student Sample before and during the Beginning of the <scp>COVID</Scp> -19 Pandemic. *International Journal of Eating Disorders* **2021**, *54* (7), 1213–1223. https://doi.org/10.1002/eat.23517.
48. Cirillo, M.; Rizzello, F.; Badolato, L.; De Angelis, D.; Evangelisti, P.; Coccia, M. E.; Fatini, C. The Effects of COVID-19 Lockdown on Lifestyle and Emotional State in Women Undergoing Assisted Reproductive Technology: Results of an Italian Survey. *J Gynecol Obstet Hum Reprod* **2021**, *50* (8), 102079. https://doi.org/10.1016/j.jogoh.2021.102079.
49. Coakley, K. E.; Le, H.; Silva, S. R.; Wilks, A. Anxiety Is Associated with Appetitive Traits in University Students during the COVID-19 Pandemic. *Nutr J* **2021**, *20* (1), 45. https://doi.org/10.1186/s12937-021-00701-9.
50. Constant, A.; Conserve, D. F.; Gallopel-Morvan, K.; Raude, J. Socio-Cognitive Factors Associated With Lifestyle Changes in Response to the COVID-19 Epidemic in the General Population: Results From a Cross-Sectional Study in France. *Front Psychol* **2020**, *11*. https://doi.org/10.3389/fpsyg.2020.579460.
51. Cooper, J. A.; vanDellen, M.; Bhutani, S. Self-Weighing Practices and Associated Health Behaviors during COVID-19. *Am J Health Behav* **2021**, *45* (1), 17–30. https://doi.org/10.5993/AJHB.45.1.2.
52. Costa, C. dos S.; Steele, E. M.; Leite, M. A.; Rauber, F.; Levy, R. B.; Monteiro, C. A. Mudanças No Peso Corporal Na Coorte NutriNet Brasil Durante a Pandemia de Covid-19. *Rev Saude Publica* **2021**, *55*, 1. https://doi.org/10.11606/s1518-8787.2021055003457.
53. Costa, M. L.; Costa, M. G. O.; de Souza, M. F. C.; da Silva, D. G.; Vieira, D. A. dos S.; Mendes-Netto, R. S. Is Physical Activity Protective against Emotional Eating Associated Factors during the COVID-19 Pandemic? A Cross-Sectional Study among Physically Active and Inactive Adults. *Nutrients* **2021**, *13* (11), 3861. https://doi.org/10.3390/nu13113861.

54. Coulthard, H.; Sharps, M.; Cunliffe, L.; van den Tol, A. Eating in the Lockdown during the Covid 19 Pandemic; Self-Reported Changes in Eating Behaviour, and Associations with BMI, Eating Style, Coping and Health Anxiety. *Appetite* **2021**, *161*, 105082. https://doi.org/10.1016/j.appet.2020.105082.
55. Crucianu, C.; Georgescu, P. L. THE CONSEQUENCES OF COVID-19 PANDEMIC ON DIET AND PHYSICAL ACTIVITY. *Archiv Euromedica* **2021**, *11* (4), 14–16. https://doi.org/10.35630/2199-885X/2021/11/4.3.
56. Cummings, J. R.; Ackerman, J. M.; Wolfson, J. A.; Gearhardt, A. N. COVID-19 Stress and Eating and Drinking Behaviors in the United States during the Early Stages of the Pandemic. *Appetite* **2021**, *162*, 105163. https://doi.org/10.1016/j.appet.2021.105163.
57. Czepczor-Bernat, K.; Swami, V.; Modrzejewska, A.; Modrzejewska, J. COVID-19-Related Stress and Anxiety, Body Mass Index, Eating Disorder Symptomatology, and Body Image in Women from Poland: A Cluster Analysis Approach. *Nutrients* **2021**, *13* (4), 1384. https://doi.org/10.3390/nu13041384.
58. da Rocha, A. Q.; Lobo, P. C. B.; Pimentel, G. D. Muscle Function Loss and Gain of Body Weight during the COVID-19 Pandemic in Elderly Women: Effects of One Year of Lockdown. *J Nutr Health Aging* **2021**, *25* (8), 1028–1029. https://doi.org/10.1007/s12603-021-1663-x.
59. De Pasquale, C.; Sciacca, F.; Conti, D.; Pistorio, M. L.; Hichy, Z.; Cardullo, R. L.; Di Nuovo, S. Relations Between Mood States and Eating Behavior During COVID-19 Pandemic in a Sample of Italian College Students. *Front Psychol* **2021**, *12*. https://doi.org/10.3389/fpsyg.2021.684195.
60. Deschasaux-Tanguy, M.; Druesne-Pecollo, N.; Esseddik, Y.; de Edelenyi, F. S.; Allès, B.; Andreeva, V. A.; Baudry, J.; Charreire, H.; Deschamps, V.; Egnell, M.; Fezeu, L. K.; Galan, P.; Julia, C.; Kesse-Guyot, E.; Latino-Martel, P.; Oppert, J.-M.; Péneau, S.; Verdot, C.; Hercberg, S.; Touvier, M. Diet and Physical Activity during the Coronavirus Disease 2019 (COVID-19) Lockdown (March–May 2020): Results from the French NutriNet-Santé Cohort Study. *Am J Clin Nutr* **2021**, *113* (4), 924–938. https://doi.org/10.1093/ajcn/nqaa336.
61. Di Renzo, L.; Gualtieri, P.; Cinelli, G.; Bigioni, G.; Soldati, L.; Attinà, A.; Bianco, F. F.; Caparello, G.; Camodeca, V.; Carrano, E.; Ferraro, S.; Giannattasio, S.; Leggeri, C.; Rampello, T.; Lo Presti, L.; Tarsitano, M. G.; De Lorenzo, A. Psychological Aspects and Eating Habits during COVID-19 Home Confinement: Results of EHLC-COVID-19 Italian Online Survey. *Nutrients* **2020**, *12* (7), 2152. https://doi.org/10.3390/nu12072152.
62. Dicken, S. J.; Mitchell, J. J.; Newberry Le Vay, J.; Beard, E.; Kale, D.; Herbec, A.; Shahab, L. Impact of COVID-19 Pandemic on Weight and BMI among UK Adults: A Longitudinal Analysis of Data from the HEBECO Study. *Nutrients* **2021**, *13* (9), 2911. https://doi.org/10.3390/nu13092911.
63. Dobrowolski, H.; Włodarek, D. Body Mass, Physical Activity and Eating Habits Changes during the First COVID-19 Pandemic Lockdown in Poland. *Int J Environ Res Public Health* **2021**, *18* (11), 5682. https://doi.org/10.3390/ijerph18115682.
64. Đogaš, Z.; Lušić Kalcina, L.; Pavlinac Dodig, I.; Demirović, S.; Madirazza, K.; Valić, M.; Pecotić, R. The Effect of COVID-19 Lockdown on Lifestyle and Mood in Croatian General Population: A Cross-Sectional Study. *Croat Med J* **2020**, *61* (4), 309–318. https://doi.org/10.3325/cmj.2020.61.309.
65. Dores, A. R.; Carvalho, I. P.; Burkauskas, J.; Simonato, P.; De Luca, I.; Mooney, R.; Ioannidis, K.; Gómez-Martínez, M. Á.; Demetrovics, Z.; Ábel, K. E.; Szabo, A.; Fujiwara, H.; Shibata, M.; Ventola, A. R. M.; Arroyo-Anlló, E. M.; Santos-Labrador, R. M.; Griskova-Bulanova, I.; Pranckeviciene, A.; Kobayashi, K.; Martinotti, G.; Fineberg, N. A.; Barbosa, F.; Corazza, O. Exercise and Use of Enhancement Drugs at the Time of the COVID-19 Pandemic: A Multicultural Study on Coping Strategies During Self-Isolation and Related Risks. *Front Psychiatry* **2021**, *12*. https://doi.org/10.3389/fpsyt.2021.648501.

66. Dor-Haim, H.; Katzburg, S.; Revach, P.; Levine, H.; Barak, S. The Impact of COVID-19 Lockdown on Physical Activity and Weight Gain among Active Adult Population in Israel: A Cross-Sectional Study. *BMC Public Health* **2021**, *21* (1), 1521. https://doi.org/10.1186/s12889-021-11523-z.
67. dos Santos Quaresma, M. V.; Marques, C. G.; Magalhães, A. C. O.; dos Santos, R. V. T. Emotional Eating, Binge Eating, Physical Inactivity, and Vespertine Chronotype Are Negative Predictors of Dietary Practices during COVID-19 Social Isolation: A Cross-Sectional Study. *Nutrition* **2021**, *90*, 111223. https://doi.org/10.1016/j.nut.2021.111223.
68. Dragun, R.; Veček, N. N.; Marendić, M.; Pribisalić, A.; Đivić, G.; Cena, H.; Polašek, O.; Kolčić, I. Have Lifestyle Habits and Psychological Well-Being Changed among Adolescents and Medical Students Due to COVID-19 Lockdown in Croatia? *Nutrients* **2020**, *13* (1), 97. https://doi.org/10.3390/nu13010097.
69. Drieskens, S.; Berger, N.; Vandevijvere, S.; Gisle, L.; Braekman, E.; Charafeddine, R.; De Ridder, K.; Demarest, S. Short-Term Impact of the COVID-19 Confinement Measures on Health Behaviours and Weight Gain among Adults in Belgium. *Archives of Public Health* **2021**, *79* (1), 22. https://doi.org/10.1186/s13690-021-00542-2.
70. Drywień, M. E.; Hamulka, J.; Zielinska-Pukos, M. A.; Jeruszka-Bielak, M.; Górnicka, M. The COVID-19 Pandemic Lockdowns and Changes in Body Weight among Polish Women. A Cross-Sectional Online Survey PLifeCOVID-19 Study. *Sustainability* **2020**, *12* (18), 7768. https://doi.org/10.3390/su12187768.
71. Du, C.; Adjepong, M.; Zan, M. C. H.; Cho, M. J.; Fenton, J. I.; Hsiao, P. Y.; Keaver, L.; Lee, H.; Ludy, M. J.; Shen, W.; Swee, W. C. S.; Thrivikraman, J.; Amoah-Agyei, F.; de Kanter, E.; Wang, W.; Tucker, R. M. Gender Differences in the Relationships between Perceived Stress, Eating Behaviors, Sleep, Dietary Risk, and Body Mass Index. *Nutrients* **2022**, *14* (5). https://doi.org/10.3390/nu14051045.
72. Dun, Y.; Ripley-Gonzalez, J. W.; Zhou, N.; You, B.; Li, Q.; Li, H.; Zhang, W.; Thomas, R. J.; Olson, T. P.; Liu, J.; Dong, Y.; Liu, S. Weight Gain in Chinese Youth during a 4-Month COVID-19 Lockdown: A Retrospective Observational Study. *BMJ Open* **2021**, *11* (7), e052451. https://doi.org/10.1136/bmjopen-2021-052451.
73. Ekpanyaskul, C.; Padungtod, C. Occupational Health Problems and Lifestyle Changes Among Novice Working-From-Home Workers Amid the COVID-19 Pandemic. *Saf Health Work* **2021**, *12* (3), 384–389. https://doi.org/10.1016/j.shaw.2021.01.010.
74. Elangovan, A.; Eapen, A.; M. Padmapriya, V.; Nagaraj, J.; Kannan, Radhakrishnan; Ravi, M.; Santhakumar, A.; Malathi, M.; Elavarasu, Govindhasamy; A. Merciline, D. Impact of Lockdown Due to Covid-19 on the Lifestyle Changes of Employees in India: A Cross-Sectional Analysis of Personnel Who Work at Office Versus Work from Home. *Asian Journal of Pharmaceutical Research and Health Care* **2020**, *13* (2), 177–186. https://doi.org/10.18311/ajprhc/2021/27687.
75. Elmacıoğlu, F.; Emiroğlu, E.; Ülker, M. T.; Özyılmaz Kırcali, B.; Oruç, S. Evaluation of Nutritional Behaviour Related to COVID-19. *Public Health Nutr* **2021**, *24* (3), 512–518. https://doi.org/10.1017/S1368980020004140.
76. Enriquez-Martinez, O. G.; Martins, M. C. T.; Pereira, T. S. S.; Pacheco, S. O. S.; Pacheco, F. J.; Lopez, K. V.; Huancahuire-Vega, S.; Silva, D. A.; Mora-Urda, A. I.; Rodriguez-Vásquez, M.; Montero López, M. P.; Molina, M. C. B. Diet and Lifestyle Changes During the COVID-19 Pandemic in Ibero-American Countries: Argentina, Brazil, Mexico, Peru, and Spain. *Front Nutr* **2021**, *8*. https://doi.org/10.3389/fnut.2021.671004.
77. Flaudias, V.; Iceta, S.; Zerhouni, O.; Rodgers, R. F.; Billieux, J.; Llorca, P.-M.; Boudesseul, J.; de Chazeron, I.; Romo, L.; Maurage, P.; Samalin, L.; Bègue, L.; Naassila, M.; Brousse, G.; Guillaume, S. COVID-19 Pandemic Lockdown and Problematic Eating Behaviors in a Student Population. *J Behav Addict* **2020**, *9* (3), 826–835. https://doi.org/10.1556/2006.2020.00053.

78. Freitas, F. da F.; de Medeiros, A. C. Q.; Lopes, F. de A. Effects of Social Distancing During the COVID-19 Pandemic on Anxiety and Eating Behavior—A Longitudinal Study. *Front Psychol* **2021**, *12*. https://doi.org/10.3389/fpsyg.2021.645754.
79. Gao, Y.; Ao, H.; Hu, X.; Wang, X.; Huang, D.; Huang, W.; Han, Y.; Zhou, C.; He, L.; Lei, X.; Gao, X. Social Media Exposure during COVID-19 Lockdowns Could Lead to Emotional Overeating via Anxiety: The Moderating Role of Neuroticism. *Appl Psychol Health Well Being* **2022**, *14* (1), 64–80. https://doi.org/10.1111/aphw.12291.
80. Grant, F.; Scalvedi, M. L.; Scognamiglio, U.; Turrini, A.; Rossi, L. Eating Habits during the COVID-19 Lockdown in Italy: The Nutritional and Lifestyle Side Effects of the Pandemic. *Nutrients* **2021**, *13* (7), 2279. https://doi.org/10.3390/nu13072279.
81. Guerrini Usubini, A.; Cattivelli, R.; Varallo, G.; Castelnuovo, G.; Molinari, E.; Giusti, E. M.; Pietrabissa, G.; Manari, T.; Filosa, M.; Franceschini, C.; Musetti, A. The Relationship between Psychological Distress during the Second Wave Lockdown of COVID-19 and Emotional Eating in Italian Young Adults: The Mediating Role of Emotional Dysregulation. *J Pers Med* **2021**, *11* (6), 569. https://doi.org/10.3390/jpm11060569.
82. Jackson, A. M.; Weaver, R. H.; Iniguez, A.; Lanigan, J. A Lifespan Perspective of Structural and Perceived Social Relationships, Food Insecurity, and Dietary Behaviors during the COVID-19 Pandemic. *Appetite* **2022**, *168*, 105717. https://doi.org/10.1016/j.appet.2021.105717.
83. Jackson, A.; Anderson, A.; Weybright, E.; Lanigan, J. Differing Experiences of Boredom During the Pandemic and Associations With Dietary Behaviors. *J Nutr Educ Behav* **2021**, *53* (8), 706–711. https://doi.org/10.1016/j.jneb.2021.04.005.
84. Jordan, A. K.; Barnhart, W. R.; Studer-Perez, E. I.; Kalantzis, M. A.; Hamilton, L.; Musher-Eizenman, D. R. 'Quarantine 15': Pre-Registered Findings on Stress and Concern about Weight Gain before/during COVID-19 in Relation to Caregivers' Eating Pathology. *Appetite* **2021**, *166*, 105580. https://doi.org/10.1016/j.appet.2021.105580.
85. Karakose, T.; Yirci, R.; Basyigit, H.; Kucukcakir, A. Investigation of Associations between the Effects of COVID-19 Fear on School Administrators and Nutrition and Problematic Eating Behaviors. *Progress in Nutrition* **2021**, *23* (2), e2021187–e2021187. https://doi.org/10.23751/pn.v23i2.11656.
86. Kaufman-Shriqui, V.; Navarro, D. A.; Raz, O.; Boaz, M. Dietary Changes and Anxiety during the Coronavirus Pandemic: A Multinational Survey. *Eur J Clin Nutr* **2022**, *76* (1), 84–92. https://doi.org/10.1038/s41430-021-00897-3.
87. Kesilmiş, İ.; Yilmaz, O.; Çelik, D. Ö. EFFECT OF COVID-19 ON AMATEUR FOOTBALL: PERSPECTIVE OF PHYSICAL ACTIVITY, NUTRITION, AND MOOD. *Int J Life Sci Pharma Res* **2021**, 156–163.
88. Khubchandani, J.; Kandiah, J.; Saiki, D. The COVID-19 Pandemic, Stress, and Eating Practices in the United States. *Eur J Investig Health Psychol Educ* **2020**, *10* (4), 950–956. https://doi.org/10.3390/ejihpe10040067.
89. Landaeta-Díaz, L.; González-Medina, G.; Agüero, S. D. Anxiety, Anhedonia and Food Consumption during the COVID-19 Quarantine in Chile. *Appetite* **2021**, *164*, 105259. https://doi.org/10.1016/j.appet.2021.105259.
90. León-Paucar, S. D.; Calderón-Olivos, B. C.; Calizaya-Milla, Y. E.; Saintila, J. Depression, Dietary Intake, and Body Image during Coronavirus Disease 2019 Quarantine in Peru: An Online Cross-Sectional Study. *SAGE Open Med* **2021**, *9*, 205031212110519. https://doi.org/10.1177/20503121211051914.
91. Liboredo, J. C.; Anastácio, L. R.; Ferreira, L. G.; Oliveira, L. A.; Della Lucia, C. M. Quarantine During COVID-19 Outbreak: Eating Behavior, Perceived Stress, and Their Independently Associated Factors in a Brazilian Sample. *Front Nutr* **2021**, *8*. https://doi.org/10.3389/fnut.2021.704619.
92. Li, X.; Li, J.; Qing, P.; Hu, W. COVID-19 and the Change in Lifestyle: Bodyweight, Time Allocation, and Food Choices. *Int J Environ Res Public Health* **2021**, *18* (19), 10552. https://doi.org/10.3390/ijerph181910552.

93. Lofrano-Prado, M. C.; do Prado, W. L.; Botero, J. P.; Cardel, M. L.; Farah, B. Q.; Oliveira, M. D.; Cucato, G. G.; Correia, M. A.; Ritti-Dias, R. M. The Same Storm but Not the Same Boat: Effects of <scp>COVID</Scp> -19 Stay-at-home Order on Mental Health in Individuals with Overweight. *Clin Obes* **2021**, *11* (1). https://doi.org/10.1111/cob.12425.
94. Ma, L.; Gao, L. W.; Lau, J. T.; Rahman, A.; Johnson, B. T.; Yan, A. F.; Shi, Z. M.; Ding, Y. X.; Nie, P.; Zheng, J. G.; Wang, Y. F.; Wang, W. D.; Xue, Q. L. Mental Distress and Its Associations with Behavioral Outcomes during the COVID-19 Pandemic: A National Survey of Chinese Adults. *Public Health* **2021**, *198*, 315–323. https://doi.org/10.1016/j.puhe.2021.07.034.
95. Madalı, B.; Alkan, Ş. B.; Örs, E. D.; Ayrancı, M.; Taşkın, H.; Kara, H. H. Emotional Eating Behaviors during the COVID-19 Pandemic: A Cross-Sectional Study. *Clin Nutr ESPEN* **2021**, *46*, 264–270. https://doi.org/10.1016/j.clnesp.2021.09.745.
96. Madan, J.; Blonquist, T.; Rao, E.; Marwaha, A.; Mehra, J.; Bharti, R.; Sharma, N.; Samaddar, R.; Pandey, S.; Mah, E.; Shete, V.; Chu, Y.; Chen, O. Effect of COVID-19 Pandemic-Induced Dietary and Lifestyle Changes and Their Associations with Perceived Health Status and Self-Reported Body Weight Changes in India: A Cross-Sectional Survey. *Nutrients* **2021**, *13* (11), 3682. https://doi.org/10.3390/nu13113682.
97. Maffoni, S.; Brazzo, S.; De Giuseppe, R.; Biino, G.; Vietti, I.; Pallavicini, C.; Cena, H. Lifestyle Changes and Body Mass Index during COVID-19 Pandemic Lockdown: An Italian Online-Survey. *Nutrients* **2021**, *13* (4), 1117. https://doi.org/10.3390/nu13041117.
98. Mahar, B.; Warsi, J.; Shah, T. Eating Disorders and Eating Pattern During Covid-19 Pandemic: A Short Bulletin. *Journal of Liaquat University of Medical & Health Sciences* **2021**, *20* (02), 157–162. https://doi.org/10.22442/jlumhs.2021.00776.
99. Malkawi, S. H.; Almhdawi, K.; Jaber, A. F.; Alqatarneh, N. S. COVID-19 Quarantine-Related Mental Health Symptoms and Their Correlates among Mothers: A Cross Sectional Study. *Matern Child Health J* **2021**, *25* (5), 695–705. https://doi.org/10.1007/s10995-020-03034-x.
100. Martínez-de-Quel, Ó.; Suárez-Iglesias, D.; López-Flores, M.; Pérez, C. A. Physical Activity, Dietary Habits and Sleep Quality before and during COVID-19 Lockdown: A Longitudinal Study. *Appetite* **2021**, *158*, 105019. https://doi.org/10.1016/j.appet.2020.105019.
101. Mason, T. B.; Barrington-Trimis, J.; Leventhal, A. M. Eating to Cope With the COVID-19 Pandemic and Body Weight Change in Young Adults. *Journal of Adolescent Health* **2021**, *68* (2), 277–283. https://doi.org/10.1016/j.jadohealth.2020.11.011.
102. Mazzolani, B. C.; Smaira, F. I.; Esteves, G. P.; Santo André, H. C.; Amarante, M. C.; Castanho, D.; Campos, K.; Benatti, F. B.; Pinto, A. J.; Roschel, H.; Gualano, B.; Nicoletti, C. F. Influence of Body Mass Index on Eating Habits and Food Choice Determinants Among Brazilian Women During the COVID-19 Pandemic. *Front Nutr* **2021**, *8*. https://doi.org/10.3389/fnut.2021.664240.
103. McAtamney, K.; Mantzios, M.; Egan, H.; Wallis, D. J. Emotional Eating during COVID-19 in the United Kingdom: Exploring the Roles of Alexithymia and Emotion Dysregulation. *Appetite* **2021**, *161*, 105120. https://doi.org/10.1016/j.appet.2021.105120.
104. Micheletti Cremasco, M.; Mulasso, A.; Moroni, A.; Testa, A.; Degan, R.; Rainoldi, A.; Rabaglietti, E. Relation among Perceived Weight Change, Sedentary Activities and Sleep Quality during COVID-19 Lockdown: A Study in an Academic Community in Northern Italy. *Int J Environ Res Public Health* **2021**, *18* (6), 2943. https://doi.org/10.3390/ijerph18062943.
105. Molina-Montes, E.; Uzhova, I.; Verardo, V.; Artacho, R.; García-Villanova, B.; Jesús Guerra-Hernández, E.; Kapsokefalou, M.; Malisova, O.; Vlassopoulos, A.; Katidi, A.; Koroušić Seljak, B.; Modic, R.; Eftimov, T.; Hren, I.; Valenčič, E.; Šatalić, Z.; Panjkota Krbavčić, I.; Vranešić Bender, D.; Giacalone, D.; Bom Frøst, M.; Konic Ristic, A.; Milesevic, J.; Nikolic, M.; Kolay, E.; Güney, M.; Kriaucioniene, V.; Czlapka-Matyasik, M.; Bykowska-Derda, A.; Kujundzic, E.; Taljić, I.; Brka, M.; Spiroski, I.; Cunha Velho, S.; Patrícia Sousa Pinto, S.; Nascimento Monteiro, I.; Adriana Pereira, J.; Dolores Ruíz-López, M.; Rodríguez-Pérez, C. Impact of COVID-19 Confinement on Eating Behaviours

across 16 European Countries: The COVIDiet Cross-National Study. *Food Qual Prefer* **2021**, *93*, 104231. https://doi.org/10.1016/j.foodqual.2021.104231.

106. Mota, I. A.; Oliveira Sobrinho, G. D. de; Morais, I. P. S.; Dantas, T. F. Impact of COVID-19 on Eating Habits, Physical Activity and Sleep in Brazilian Healthcare Professionals. *Arq Neuropsiquiatr* **2021**, *79* (5), 429–436. https://doi.org/10.1590/0004-282x-anp-2020-0482.
107. Mulugeta, W.; Desalegn, H.; Solomon, S. Impact of the <scp>COVID</Scp> -19 Pandemic Lockdown on Weight Status and Factors Associated with Weight Gain among Adults in Massachusetts. *Clin Obes* **2021**, *11* (4). https://doi.org/10.1111/cob.12453.
108. Mumena, W. Impact of COVID-19 Curfew on Eating Habits, Eating Frequency, and Weight According to Food Security Status in Saudi Arabia: A Retrospective Study. *Progress in Nutrition* **2020**, *22*, 1–9.
109. Nitu, I.; Rus, V. A.; Sipos, R. S.; Nyulas, T.; Cherhat, M. P.; Ruta, F.; Tita, C. C. N. Assessment of Eating Behavior During the COVID-19 Pandemic Period. A Pilot Study. *Journal of Interdisciplinary Medicine* **2021**, *6* (2), 67–73. https://doi.org/10.2478/jim-2021-0024.
110. Özcan, B.; Yeşİlkaya, B. Adverse Effect of Emotional Eating Developed During the COVID-19 Pandemic on Healthy Nutrition, a Vicious Circle: A Cross-Sectional Descriptive Study. *Revista Española de Nutrición Humana y Dietética* **2021**, *25*, e1144. https://doi.org/10.14306/renhyd.25.S2.1144.
111. Özden, G.; Parlar Kiliç, S. The Effect of Social Isolation during COVID-19 Pandemic on Nutrition and Exercise Behaviors of Nursing Students. *Ecol Food Nutr* **2021**, *60* (6), 663–681. https://doi.org/10.1080/03670244.2021.1875456.
112. Özen, G.; Eskici, G.; Yurdakul, H. Ö.; Koç, H. Assessment of the Impact of COVID-19 Pandemic on Emotional and Nutritional Status of University Athletes. *Physical education of students* **2021**, *25* (1), 43–50. https://doi.org/10.15561/20755279.2021.0106.
113. Özenoğlu, A.; Çevik, E.; Çolak, H.; Altıntaş, T.; Alakuş, K. Changes in Nutrition and Lifestyle Habits during the COVID-19 Pandemic in Turkey and the Effects of Healthy Eating Attitudes. *Med J Nutrition Metab* **2021**, *14* (3), 325–341. https://doi.org/10.3233/MNM-210562.
114. Pak, H.; Süsen, Y.; Denizci Nazlıgül, M.; Griffiths, M. The Mediating Effects of Fear of COVID-19 and Depression on the Association Between Intolerance of Uncertainty and Emotional Eating During the COVID-19 Pandemic in Turkey. *Int J Ment Health Addict* **2022**, *20* (3), 1882–1896. https://doi.org/10.1007/s11469-021-00489-z.
115. Palmer, K.; Bschaden, A.; Stroebele-Benschop, N. Changes in Lifestyle, Diet, and Body Weight during the First COVID 19 'Lockdown' in a Student Sample. *Appetite* **2021**, *167*, 105638. https://doi.org/10.1016/j.appet.2021.105638.
116. Pappa, S.; Barnett, J.; Berges, I.; Sakkas, N. Tired, Worried and Burned Out, but Still Resilient: A Cross-Sectional Study of Mental Health Workers in the UK during the COVID-19 Pandemic. *Int J Environ Res Public Health* **2021**, *18* (9), 4457. https://doi.org/10.3390/ijerph18094457.
117. Pertuz-Cruz, S. L.; Molina-Montes, E.; Rodríguez-Pérez, C.; Guerra-Hernández, E. J.; Cobos de Rangel, O. P.; Artacho, R.; Verardo, V.; Ruiz-Lopez, M. D.; García-Villanova, B. Exploring Dietary Behavior Changes Due to the COVID-19 Confinement in Colombia: A National and Regional Survey Study. *Front Nutr* **2021**, *8*. https://doi.org/10.3389/fnut.2021.644800.
118. Phillipou, A.; Meyer, D.; Neill, E.; Tan, E. J.; Toh, W. L.; Van Rheenen, T. E.; Rossell, S. L. Eating and Exercise Behaviors in Eating Disorders and the General Population during the <scp>COVID</Scp> -19 Pandemic in Australia: Initial Results from the <scp>COLLATE</Scp> Project. *International Journal of Eating Disorders* **2020**, *53* (7), 1158–1165. https://doi.org/10.1002/eat.23317.
119. Phillipou, A.; Tan, E. J.; Toh, W. L.; Van Rheenen, T. E.; Meyer, D.; Neill, E.; Sumner, P.; Rossell, S. L. Mental Health of Individuals with and without Eating Disorders across Six Months and

Two Waves of COVID-19. *Eat Behav* **2021**, *43*, 101564. https://doi.org/10.1016/j.eatbeh.2021.101564.
120. Pirutinsky, S.; Cherniak, A. D.; Rosmarin, D. H. COVID-19, Religious Coping, and Weight Change in the Orthodox Jewish Community. *J Relig Health* **2021**, *60* (2), 646–653. https://doi.org/10.1007/s10943-021-01196-8.
121. Pišot, S.; Milovanović, I.; Šimunič, B.; Gentile, A.; Bosnar, K.; Prot, F.; Bianco, A.; Lo Coco, G.; Bartoluci, S.; Katović, D.; Bakalár, P.; Kovalik Slančová, T.; Tlučáková, L.; Casals, C.; Feka, K.; Christogianni, A.; Drid, P. Maintaining Everyday Life Praxis in the Time of COVID-19 Pandemic Measures (ELP-COVID-19 Survey). *Eur J Public Health* **2020**, *30* (6), 1181–1186. https://doi.org/10.1093/eurpub/ckaa157.
122. Poelman, M. P.; Gillebaart, M.; Schlinkert, C.; Dijkstra, S. C.; Derksen, E.; Mensink, F.; Hermans, R. C. J.; Aardening, P.; de Ridder, D.; de Vet, E. Eating Behavior and Food Purchases during the COVID-19 Lockdown: A Cross-Sectional Study among Adults in the Netherlands. *Appetite* **2021**, *157*, 105002. https://doi.org/10.1016/j.appet.2020.105002.
123. Pompili, S.; Di Tata, D.; Bianchi, D.; Lonigro, A.; Zammuto, M.; Baiocco, R.; Longobardi, E.; Laghi, F. Food and Alcohol Disturbance among Young Adults during the COVID-19 Lockdown in Italy: Risk and Protective Factors. *Eating and Weight Disorders - Studies on Anorexia, Bulimia and Obesity* **2022**, *27* (2), 769–780. https://doi.org/10.1007/s40519-021-01220-6.
124. Pop, C.; Ciomag, V. Impact of COVID-19 Lockdown on Body Mass Index in Young Adults. *Physical education of students* **2021**, *25* (2), 98–102. https://doi.org/10.15561/20755279.2021.0204.
125. Prezotti, J. A.; Henriques, J. V. T.; Favorito, L. A.; Canalini, A. F.; Machado, M. G.; Brandão, T. B. V.; Barbosa, A. M. V.; Moromizato, J. K. M.; Anzolch, K. M. J.; Fernandes, R. de C.; Rodrigues, F. R. A.; Bellucci, C. H. S.; Silva, C. S.; Pompeo, A. C. L.; Bessa Jr., J. de; Gomes, C. M. Impact of COVID-19 on Education, Health and Lifestyle Behaviour of Brazilian Urology Residents. *International braz j urol* **2021**, *47* (4), 753–776. https://doi.org/10.1590/s1677-5538.ibju.2021.99.09.
126. Puhl, R. M.; Lessard, L. M.; Larson, N.; Eisenberg, M. E.; Neumark-Stzainer, D. Weight Stigma as a Predictor of Distress and Maladaptive Eating Behaviors During COVID-19: Longitudinal Findings From the EAT Study. *Annals of Behavioral Medicine* **2020**, *54* (10), 738–746. https://doi.org/10.1093/abm/kaaa077.
127. Queiroz, F. L. N. de; Nakano, E. Y.; Botelho, R. B. A.; Ginani, V. C.; Raposo, A.; Zandonadi, R. P. Eating Competence among Brazilian Adults: A Comparison between before and during the COVID-19 Pandemic. *Foods* **2021**, *10* (9), 2001. https://doi.org/10.3390/foods10092001.
128. Radwan, H.; Al Kitbi, M.; Hasan, H.; Al Hilali, M.; Abbas, N.; Hamadeh, R.; Saif, E. R.; Naja, F. Indirect Health Effects of COVID-19: Unhealthy Lifestyle Behaviors during the Lockdown in the United Arab Emirates. *Int J Environ Res Public Health* **2021**, *18* (4), 1964. https://doi.org/10.3390/ijerph18041964.
129. Ramalho, S. M.; Trovisqueira, A.; de Lourdes, M.; Gonçalves, S.; Ribeiro, I.; Vaz, A. R.; Machado, P. P. P.; Conceição, E. The Impact of COVID-19 Lockdown on Disordered Eating Behaviors: The Mediation Role of Psychological Distress. *Eating and Weight Disorders - Studies on Anorexia, Bulimia and Obesity* **2022**, *27* (1), 179–188. https://doi.org/10.1007/s40519-021-01128-1.
130. Reyes-Olavarría, D.; Latorre-Román, P. Á.; Guzmán-Guzmán, I. P.; Jerez-Mayorga, D.; Caamaño-Navarrete, F.; Delgado-Floody, P. Positive and Negative Changes in Food Habits, Physical Activity Patterns, and Weight Status during COVID-19 Confinement: Associated Factors in the Chilean Population. *Int J Environ Res Public Health* **2020**, *17* (15), 5431. https://doi.org/10.3390/ijerph17155431.
131. Robertson, M.; Duffy, F.; Newman, E.; Prieto Bravo, C.; Ates, H. H.; Sharpe, H. Exploring Changes in Body Image, Eating and Exercise during the COVID-19 Lockdown: A UK Survey. *Appetite* **2021**, *159*, 105062. https://doi.org/10.1016/j.appet.2020.105062.

132. Robinson, E.; Daly, M. Explaining the Rise and Fall of Psychological Distress during the COVID-19 Crisis in the United States: Longitudinal Evidence from the Understanding America Study. *Br J Health Psychol* **2021**, *26* (2), 570–587. https://doi.org/10.1111/bjhp.12493.
133. Robinson, E.; Gillespie, S.; Jones, A. Weight-related Lifestyle Behaviours and the COVID-19 Crisis: An Online Survey Study of UK Adults during Social Lockdown. *Obes Sci Pract* **2020**, *6* (6), 735–740. https://doi.org/10.1002/osp4.442.
134. Rodríguez-Pérez, C.; Molina-Montes, E.; Verardo, V.; Artacho, R.; García-Villanova, B.; Guerra-Hernández, E. J.; Ruíz-López, M. D. Changes in Dietary Behaviours during the COVID-19 Outbreak Confinement in the Spanish COVIDiet Study. *Nutrients* **2020**, *12* (6), 1730. https://doi.org/10.3390/nu12061730.
135. Rogers, A. M.; Lauren, B. N.; Woo Baidal, J. A.; Ozanne, E. M.; Hur, C. Persistent Effects of the COVID-19 Pandemic on Diet, Exercise, Risk for Food Insecurity, and Quality of Life: A Longitudinal Study among U.S. Adults. *Appetite* **2021**, *167*, 105639. https://doi.org/10.1016/j.appet.2021.105639.
136. Ruiz-Zaldibar, C.; García-Garcés, L.; Vicario-Merino, Á.; Mayoral-Gonzalo, N.; Lluesma-Vidal, M.; Ruiz-López, M.; Pérez-Manchón, D. The Impact of COVID-19 on the Lifestyles of University Students: A Spanish Online Survey. *Healthcare* **2022**, *10* (2), 309. https://doi.org/10.3390/healthcare10020309.
137. Ruiz, M. C.; Devonport, T. J.; Chen-Wilson, C.-H. (Josephine); Nicholls, W.; Cagas, J. Y.; Fernandez-Montalvo, J.; Choi, Y.; Robazza, C. A Cross-Cultural Exploratory Study of Health Behaviors and Wellbeing During COVID-19. *Front Psychol* **2021**, *11*. https://doi.org/10.3389/fpsyg.2020.608216.
138. Sadler, J. R.; Thapaliya, G.; Jansen, E.; Aghababian, A. H.; Smith, K. R.; Carnell, S. COVID-19 Stress and Food Intake: Protective and Risk Factors for Stress-Related Palatable Food Intake in U.S. Adults. *Nutrients* **2021**, *13* (3), 901. https://doi.org/10.3390/nu13030901.
139. Sánchez, E.; Lecube, A.; Bellido, D.; Monereo, S.; Malagón, M.; Tinahones, F. Leading Factors for Weight Gain during COVID-19 Lockdown in a Spanish Population: A Cross-Sectional Study. *Nutrients* **2021**, *13* (3), 894. https://doi.org/10.3390/nu13030894.
140. Sánchez-Sánchez, E.; Ramírez-Vargas, G.; Avellaneda-López, Y.; Orellana-Pecino, J. I.; García-Marín, E.; Díaz-Jimenez, J. Eating Habits and Physical Activity of the Spanish Population during the COVID-19 Pandemic Period. *Nutrients* **2020**, *12* (9), 2826. https://doi.org/10.3390/nu12092826.
141. Sánchez-Sánchez, E.; Díaz-Jimenez, J.; Rosety, I.; Alférez, M. J. M.; Díaz, A. J.; Rosety, M. A.; Ordonez, F. J.; Rosety-Rodriguez, M. Perceived Stress and Increased Food Consumption during the 'Third Wave' of the COVID-19 Pandemic in Spain. *Nutrients* **2021**, *13* (7), 2380. https://doi.org/10.3390/nu13072380.
142. Santana, J. da M.; Milagres, M. P.; Silva dos Santos, C.; Brazil, J. M.; Lima, E. R.; Pereira, M. Dietary Intake of University Students during COVID-19 Social Distancing in the Northeast of Brazil and Associated Factors. *Appetite* **2021**, *162*, 105172. https://doi.org/10.1016/j.appet.2021.105172.
143. Sarda, B.; Delamaire, C.; Serry, A.-J.; Ducrot, P. Changes in Home Cooking and Culinary Practices among the French Population during the COVID-19 Lockdown. *Appetite* **2022**, *168*, 105743. https://doi.org/10.1016/j.appet.2021.105743.
144. Saxena, R.; Parmar, N.; Kaur, P.; Allen, T. Effect of Screen-Time on Sleep Pattern and Dietary Habits among College-Going Students in COVID-19 Pandemic. *Indian J Community Health* **2021**, *33* (1), 65–74. https://doi.org/10.47203/IJCH.2021.v33i01.009.
145. Scacchi, A.; Catozzi, D.; Boietti, E.; Bert, F.; Siliquini, R. COVID-19 Lockdown and Self-Perceived Changes of Food Choice, Waste, Impulse Buying and Their Determinants in Italy: QuarantEat, a Cross-Sectional Study. *Foods* **2021**, *10* (2), 306. https://doi.org/10.3390/foods10020306.

146. Scarmozzino, F.; Visioli, F. Covid-19 and the Subsequent Lockdown Modified Dietary Habits of Almost Half the Population in an Italian Sample. *Foods* **2020**, *9* (5), 675. https://doi.org/10.3390/foods9050675.
147. Scharmer, C.; Martinez, K.; Gorrell, S.; Reilly, E. E.; Donahue, J. M.; Anderson, D. A. Eating Disorder Pathology and Compulsive Exercise during the <scp>COVID</Scp> -19 Public Health Emergency: Examining Risk Associated with <scp>COVID</Scp> -19 Anxiety and Intolerance of Uncertainty. *International Journal of Eating Disorders* **2020**, *53* (12), 2049–2054. https://doi.org/10.1002/eat.23395.
148. Schulte, E. M.; Kral, T. V. E.; Allison, K. C. A Cross-Sectional Examination of Reported Changes to Weight, Eating, and Activity Behaviors during the COVID-19 Pandemic among United States Adults with Food Addiction. *Appetite* **2022**, *168*, 105740. https://doi.org/10.1016/j.appet.2021.105740.
149. Seal, A.; Schaffner, A.; Phelan, S.; Brunner-Gaydos, H.; Tseng, M.; Keadle, S.; Alber, J.; Kiteck, I.; Hagobian, T. COVID-19 Pandemic and Stay-at-home Mandates Promote Weight Gain in US Adults. *Obesity* **2022**, *30* (1), 240–248. https://doi.org/10.1002/oby.23293.
150. Sebastião, E.; Steffens, M.; Nakamura, P. M.; Papini, C. B. Perceptions on Activity Behavior during the COVID-19 Pandemic "Second Wave" among US Adults: Results of a Short Online Survey. *Sport Sci Health* **2022**, *18* (1), 267–275. https://doi.org/10.1007/s11332-021-00813-z.
151. Serin, E.; Can Koç, M. Examination of the Eating Behaviours and Depression States of the University Students Who Stay at Home during the Coronavirus Pandemic in Terms of Different Variables. *Progress in Nutrition* **2020**, *22*, 33–43.
152. Shaun, M. M. A.; Nizum, M. W. R.; Munny, S.; Fayeza, F.; Mali, S. K.; Abid, M. T.; Hasan, A.-R. Eating Habits and Lifestyle Changes among Higher Studies Students Post-Lockdown in Bangladesh: A Web-Based Cross-Sectional Study. *Heliyon* **2021**, *7* (8), e07843. https://doi.org/10.1016/j.heliyon.2021.e07843.
153. Shibata, M.; Burkauskas, J.; Dores, A. R.; Kobayashi, K.; Yoshimura, S.; Simonato, P.; De Luca, I.; Cicconcelli, D.; Giorgetti, V.; Carvalho, I. P.; Barbosa, F.; Monteiro, C.; Murai, T.; Gómez-Martínez, M. A.; Demetrovics, Z.; Ábel, K. E.; Szabo, A.; Ventola, A. R. M.; Arroyo-Anlló, E. M.; Santos-Labrador, R. M.; Griskova-Bulanova, I.; Pranckeviciene, A.; Bersani, G.; Fujiwara, H.; Corazza, O. Exploring the Relationship Between Mental Well-Being, Exercise Routines, and the Intake of Image and Performance Enhancing Drugs During the Coronavirus Disease 2019 Pandemic: A Comparison Across Sport Disciplines. *Front Psychol* **2021**, *12*. https://doi.org/10.3389/fpsyg.2021.689058.
154. Shin, E. Pandemic Fear and Weight Gain: Effects on Overweight and Obese Adults' Purchasing Exercise Apparel Online. *Clothing and Textiles Research Journal* **2021**, *39* (3), 232–246. https://doi.org/10.1177/0887302X211004892.
155. Sidor, A.; Rzymski, P. Dietary Choices and Habits during COVID-19 Lockdown: Experience from Poland. *Nutrients* **2020**, *12* (6), 1657. https://doi.org/10.3390/nu12061657.
156. Silva, M. N.; Gregório, M. J.; Santos, R.; Marques, A.; Rodrigues, B.; Godinho, C.; Silva, C. S.; Mendes, R.; Graça, P.; Arriaga, M.; Freitas, G. Towards an In-Depth Understanding of Physical Activity and Eating Behaviours during COVID-19 Social Confinement: A Combined Approach from a Portuguese National Survey. *Nutrients* **2021**, *13* (8), 2685. https://doi.org/10.3390/nu13082685.
157. Silverman, J. R.; Wang, B. Z. Impact of School Closures, Precipitated by COVID-19, on Weight and Weight-Related Risk Factors among Schoolteachers: A Cross-Sectional Study. *Nutrients* **2021**, *13* (8), 2723. https://doi.org/10.3390/nu13082723.
158. Skotnicka, M.; Karwowska, K.; Kłobukowski, F.; Wasilewska, E.; Małgorzewicz, S. Dietary Habits before and during the COVID-19 Epidemic in Selected European Countries. *Nutrients* **2021**, *13* (5), 1690. https://doi.org/10.3390/nu13051690.

159. Smith, K. R.; Jansen, E.; Thapaliya, G.; Aghababian, A. H.; Chen, L.; Sadler, J. R.; Carnell, S. The Influence of COVID-19-Related Stress on Food Motivation. *Appetite* **2021**, *163*, 105233. https://doi.org/10.1016/j.appet.2021.105233.
160. Sobba, W.; Landry, M. J.; Cunanan, K. M.; Marcone, A.; Gardner, C. D. Changes in Ultra-Processed Food Consumption and Lifestyle Behaviors Following COVID-19 Shelter-in-Place: A Retrospective Study. *Foods* **2021**, *10* (11), 2553. https://doi.org/10.3390/foods10112553.
161. Solé, B.; Verdolini, N.; Amoretti, S.; Montejo, L.; Rosa, A. R.; Hogg, B.; Garcia-Rizo, C.; Mezquida, G.; Bernardo, M.; Martinez-Aran, A.; Vieta, E.; Torrent, C. Effects of the COVID-19 Pandemic and Lockdown in Spain: Comparison between Community Controls and Patients with a Psychiatric Disorder. Preliminary Results from the BRIS-MHC STUDY. *J Affect Disord* **2021**, *281*, 13–23. https://doi.org/10.1016/j.jad.2020.11.099.
162. Suka, M.; Yamauchi, T.; Yanagisawa, H. Changes in Health Status, Workload, and Lifestyle after Starting the COVID-19 Pandemic: A Web-Based Survey of Japanese Men and Women. *Environ Health Prev Med* **2021**, *26* (1), 37. https://doi.org/10.1186/s12199-021-00957-x.
163. Sulejmani, E.; Hyseni, A.; Xhabiri, G.; Rodríguez-Pérez, C. Relationship in Dietary Habits Variations during COVID-19 Lockdown in Kosovo: The COVIDiet Study. *Appetite* **2021**, *164*, 105244. https://doi.org/10.1016/j.appet.2021.105244.
164. Swami, V.; Horne, G.; Furnham, A. COVID-19-Related Stress and Anxiety Are Associated with Negative Body Image in Adults from the United Kingdom. *Pers Individ Dif* **2021**, *170*, 110426. https://doi.org/10.1016/j.paid.2020.110426.
165. Tabler, J.; Schmitz, R. M.; Charak, R.; Dickinson, E. Perceived Weight Gain and Eating Disorder Symptoms among LGBTQ+ Adults during the COVID-19 Pandemic: A Convergent Mixed-Method Study. *J Eat Disord* **2021**, *9* (1), 115. https://doi.org/10.1186/s40337-021-00470-0.
166. Tan, S. T.; Tan, C. X.; Tan, S. S. Trajectories of Food Choice Motives and Weight Status of Malaysian Youths during the COVID-19 Pandemic. *Nutrients* **2021**, *13* (11), 3752. https://doi.org/10.3390/nu13113752.
167. Tfifha, M.; Abbes, W.; Dhemaid, M.; Mdhaffar, K.; Abbes, M.; Zitoun, K.; Ghanmi, L. Binge Eating Disorder Experienced by Young Doctors Struggling with COVID-19. *European Psychiatry* **2021**, *64* (S1), S285–S286. https://doi.org/10.1192/j.eurpsy.2021.765.
168. Thahir, A.; Sulastri, S.; Zahra Bulantika, S.; Novita, T. Gender Differences on COVID-19 Related Anxiety Among Students. *Pakistan Journal of Psychological Research* **2021**, *36* (1), 71–83. https://doi.org/10.33824/PJPR.2021.36.1.05.
169. Trott, M.; Johnstone, J.; Pardhan, S.; Barnett, Y.; Smith, L. Changes in Body Dysmorphic Disorder, Eating Disorder, and Exercise Addiction Symptomology during the COVID-19 Pandemic: A Longitudinal Study of 319 Health Club Users. *Psychiatry Res* **2021**, *298*, 113831. https://doi.org/10.1016/j.psychres.2021.113831.
170. Turgut, M.; Soylu, Y.; Metin, S. N. Physical Activity, Night Eating, and Mood State Profiles of Athletes during the COVID-19 Pandemic. *Progress in Nutrition* **2020**, *22*, e2020019.
171. Urzeala, C.; Duclos, M.; Chris Ugbolue, U.; Bota, A.; Berthon, M.; Kulik, K.; Thivel, D.; Bagheri, R.; Gu, Y.; Baker, J. S.; Andant, N.; Pereira, B.; Rouffiac, K.; Clinchamps, M.; Dutheil, F.; Mestres, S.; Miele, C.; Navel, V.; Parreira, L.; Boirie, Y.; Bouillon-Minois, J.; Fantini, M. L.; Schmidt, J.; Tubert-Jeannin, S.; Chausse, P.; Dambrun, M.; Droit-Volet, S.; Guegan, J.; Guimond, S.; Mondillon, L.; Nugier, A.; Huguet, P.; Dewavrin, S.; Marhar, F.; Naughton, G.; Benson, A.; Lamm, C.; Drapeau, V.; Avilés Dorlhiac, R.; Bustos, B.; Zhang, H.; Dieckmann, P.; Quach, B.; Duan, Y.; Gao, G.; Huang, W. Y. J.; Lau, K. L. K.; Zhang, C.; Jiao, J.; Chen, K.; Nasir, H.; Cocco, P.; Lecca, R.; Puligheddu, M.; Figorilli, M.; Charkhabi, M.; Pfabigan, D.; Dieckmann, P.; Antunes, S.; Neto, D.; Almeida, P.; Gouveia, M. J.; Quinteiro, P.; Dubuis, B.; Lemaignen, J.; Liu, A.; Saadaoui, F. COVID-19 Lockdown Consequences on Body Mass Index and Perceived Fragility Related to Physical Activity: A Worldwide Cohort Study. *Health Expectations* **2022**, *25* (2), 522–531. https://doi.org/10.1111/hex.13282.

172. Vacca, M.; De Maria, A.; Mallia, L.; Lombardo, C. Perfectionism and Eating Behavior in the COVID-19 Pandemic. *Front Psychol* **2021**, *12*. https://doi.org/10.3389/fpsyg.2021.580943.
173. Valencia, D.; Ghani, S.; Delgadillo, M.; Madhivanan, P.; Krupp, K.; Ruiz, J.; Seixas, A.; Jean-Louis, G.; Killgore, W.; Wills, C.; Grandner, M. 202 COVID-19 Pandemic Sleep Disturbances Related To Dietary Behavior at the US-Mexico Border. *Sleep* **2021**, *44* (Supplement_2), A81–A81. https://doi.org/10.1093/sleep/zsab072.201.
174. Vidal, L.; Brunet, G.; Curutchet, M. R.; Girona, A.; Pardiñas, V.; Guerra, D.; Platero, E.; Machado, L.; González, F.; Gugliucci, V.; Ares, G. Is COVID-19 a Threat or an Opportunity for Healthy Eating? An Exploration of the Factors That Moderate the Impact of the Pandemic on Eating Habits in Uruguay. *Appetite* **2021**, *167*, 105651. https://doi.org/10.1016/j.appet.2021.105651.
175. Visser, M.; Schaap, L. A.; Wijnhoven, H. A. H. Self-Reported Impact of the COVID-19 Pandemic on Nutrition and Physical Activity Behaviour in Dutch Older Adults Living Independently. *Nutrients* **2020**, *12* (12), 3708. https://doi.org/10.3390/nu12123708.
176. Wang, R.; Ye, B.; Wang, P.; Tang, C.; Yang, Q. Coronavirus Stress and Overeating: The Role of Anxiety and COVID-19 Burnout. *J Eat Disord* **2022**, *10* (1). https://doi.org/10.1186/s40337-022-00584-z.
177. Yang, S.; Guo, B.; Ao, L.; Yang, C.; Zhang, L.; Zhou, J.; Jia, P. Obesity and Activity Patterns before and during <scp>COVID</Scp> -19 Lockdown among Youths in China. *Clin Obes* **2020**, *10* (6). https://doi.org/10.1111/cob.12416.
178. Yılmaz Akyüz, E.; Yılmaz Onal, H.; Yuksel, A. Nutritional Habits and Emotional Eating of Adults during Social Isolation Days Due to Covid-19 Pandemic. *Progress in Health Sciences* **2021**, *11* (1), 43–56. https://doi.org/10.5604/01.3001.0014.9280.
179. Yılmaz, S.; Sanlier, N.; Göbel, P.; Açıkalın, B.; Kocabas, S.; Dundar, A. The Dark Side of the Quarantine: Night Eating, Sleep Quality and the Health Locus of Control in Women. *Nutr Food Sci* **2022**, *52* (4), 627–640. https://doi.org/10.1108/NFS-08-2021-0246.
180. Yokoro, M.; Wakimoto, K.; Otaki, N.; Fukuo, K. Increased Prevalence of Breakfast Skipping in Female College Students in COVID-19. *Asia Pacific Journal of Public Health* **2021**, *33* (4), 438–440. https://doi.org/10.1177/1010539521998861.
181. Zach, S.; Fernandez-Rio, J.; Zeev, A.; Ophir, M.; Eilat-Adar, S. Physical Activity, Resilience, Emotions, Moods, and Weight Control, during the COVID-19 Global Crisis. *Isr J Health Policy Res* **2021**, *10* (1), 52. https://doi.org/10.1186/s13584-021-00473-x.
182. Zachary, Z.; Brianna, F.; Brianna, L.; Garrett, P.; Jade, W.; Alyssa, D.; Mikayla, K. Self-Quarantine and Weight Gain Related Risk Factors during the COVID-19 Pandemic. *Obes Res Clin Pract* **2020**, *14* (3), 210–216. https://doi.org/10.1016/j.orcp.2020.05.004.
183. Zhang, J.; Zhang, Y.; Huo, S.; Ma, Y.; Ke, Y.; Wang, P.; Zhao, A. Emotional Eating in Pregnant Women during the COVID-19 Pandemic and Its Association with Dietary Intake and Gestational Weight Gain. *Nutrients* **2020**, *12* (8), 2250. https://doi.org/10.3390/nu12082250.
184. Zhou, Y.; Wade, T. D. The Impact of <scp>COVID</Scp> -19 on Body-dissatisfied Female University Students. *International Journal of Eating Disorders* **2021**, *54* (7), 1283–1288. https://doi.org/10.1002/eat.23521.
185. Zhu, Q.; Li, M.; Ji, Y.; Shi, Y.; Zhou, J.; Li, Q.; Qin, R.; Zhuang, X. “Stay-at-Home” Lifestyle Effect on Weight Gain during the COVID-19 Outbreak Confinement in China. *Int J Environ Res Public Health* **2021**, *18* (4), 1813. https://doi.org/10.3390/ijerph18041813.
186. Zielińska, M.; Łuszczki, E.; Bartosiewicz, A.; Wyszyńska, J.; Dereń, K. The Prevalence of ‘“Food Addiction”’ during the COVID-19 Pandemic Measured Using the Yale Food Addiction Scale 2.0 (YFAS 2.0) among the Adult Population of Poland. *Nutrients* **2021**, *13* (11), 4115. https://doi.org/10.3390/nu13114115.
